# Interpretable Machine‐Learning and Big Data Mining to Predict the CO_2_ Separation in Polymer‐MOF Mixed Matrix Membranes

**DOI:** 10.1002/advs.202405905

**Published:** 2025-02-27

**Authors:** Hao Wan, Yue Fang, Min Hu, Shuya Guo, Zhiqiang Sui, Xiaoshan Huang, Zili Liu, Yue Zhao, Hong Liang, Yufang Wu, Hanyu Gao, Zhiwei Qiao

**Affiliations:** ^1^ Guangzhou Key Laboratory for New Energy and Green Catalysis School of Chemistry and Chemical Engineering Guangzhou University Guangzhou 510006 P. R. China; ^2^ Department of Chemical and Biological Engineering Hong Kong University of Science and Technology Clear Water Bay Hong Kong 999077 P. R. China; ^3^ State Key Laboratory of NBC Protection for Civilian Beijing 100191 P. R. China; ^4^ Joint Institute of Guangzhou University & Institute of Corrosion Science and Technology Guangzhou University Guangzhou 510006 P. R. China

**Keywords:** CO_2_ separation, machine learning, Mixed matrix membranes, molecular simulation, transfer learning

## Abstract

Mixed matrix membranes (MMMs) are renowned for their exceptional gas separation capabilities. In this work, high‐throughput computing screening and machine learning are employed to evaluate the CO_2_ separation performance of 54117 MMMs composed of 9 polymers and 6013 metal–organic frameworks (MOFs). The structure‐property relationships of MMMs are analyzed for 4 binary mixtures (CO_2_/X, X = CH_4_, N_2_, H_2_, O_2_), and the best‐performing combinations of MOFs and polymers are found, with which the MMM performance exceeded the Robeson's upper limit. Then, a stacked ensemble regression model with high accuracy (average *R^2^* = 0.96) is trained, demonstrating excellent extrapolation capability (*R^2^* = 0.95) for new MMMs containing 6FDA‐DAM. Furthermore, by utilizing Shapley Additive Explanations and data segmentation, it is identified that the pore limit diameter and largest cavity diameter in MOF features and the fractional free volume and density in polymer features are of paramount importance. Two extrapolation methods are compared and found that transfer learning is better for predicting CO_2_ separation performance in MMMs and designing new materials with large datasets. Finally, an interactive desktop software is developed to assist researchers in rapidly and accurately calculating the CO_2_ separation performance of MMMs. This work presents a novel approach for the rapid evaluation of high‐quality MMMs and the efficient calculation of gas permeation rates within membranes.

## Graphical abstract

To explore the structure‐property relationships of MMM (Mixed Matrix Membranes), the big data mining is employed and a transfer learning model is trained for predicting and selecting the optimal MMM materials. It achieves fast computation for the separation of CO_2_ in MMM.

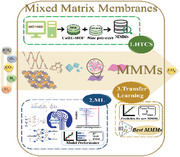

## 1 Introduction

Excessive carbon dioxide (CO_2_) emissions are the main cause of global warming, and CO_2_ emissions from coal‐fired power plants account for a significant portion of the global emissions. To reduce CO_2_ emissions, attention needs to be paid to technologies for capturing CO_2_.^[^ ^1^ ^]^ Additionally, CO_2_ separation is also of great significance in fields such as chemical engineering, energy, and environmental engineering.^[^ ^2^ ^]^ Traditional CO_2_ capture technique, such as low‐temperature separation and absorption with amine solvent,^[^ ^3^ ^]^ are usually expensive and energy‐intensive, limiting their applications. The emerging membrane separation technology is considered a potential solution due to its advantages of low energy consumption, less equipment requirements, and good mechanical properties.^[^ ^4^ ^]^ And membrane is the key of the membrane separation technology.

Based on the material types, conventional membranes can be divided into two categories: inorganic membranes and organic membranes (also known as polymer membranes). Compared to polymer membranes, inorganic membranes typically exhibit higher gas selectivity, mechanical strength, and thermal stability due to their unique structure and material properties.^[^ ^5^ ^]^ But the fabrication of defect‐free inorganic membrane for large‐scale operation is still challenging, and the brittleness, and operation cost also matter.^[^ ^6^ ^]^ In contrast, polymer membranes have the advantages of flexible, low cost, and easy preparation in large scale production.^[^ ^7^ ^]^ However, as proposed by Robeson a trade‐off phenomenon exists in pure polymer membranes: high selectivity inevitably leads to low permeability, while high permeability inevitably leads to low selectivity.^[^ ^8^ ^]^ Thus, the development of a new membrane to overcome the boundaries of the polymeric and inorganic membranes for gas separation is still an outstanding challenge. Then the mixed matrix membranes (MMMs) emerge. Recent studies^[^ ^9^ ^]^ have shown that combining polymer films with inorganic particles to form  MMMs can significantly enhance the gas permeability,^[^ ^10^ ^]^ and exhibit the same or even better gas selectivity.^[^ ^11^ ^]^ Inorganic nanomaterials that can be dispersed into polymer matrices mainly include zeolites,^[^ ^12^ ^]^ silica nanoparticles^[^ ^13^ ^]^ and carbon molecular sieves^[^ ^14^ ^]^ et al.^[^ ^15^ ^]^ Metal–organic frameworks^[^ ^16^ ^]^ (MOFs), a kind of highly porous crystalline materials formed by inorganic metals and organic linkers, are inorganic–organic hybrid materials. Studies have shown that the MMMs made by incorporating MOFs with high CO_2_ selectivity and permeability into polymers exhibited improved selectivity and permeability compared to pure polymers.^[^ ^17^ ^]^ Therefore, how to efficiently screen and design polymer‐MOFs MMMs with high CO_2_ separation performance has become an important research direction.

Due to the difficulty of traditional experimental methods to cope with large‐scale MMMs design, especially for screening optimal MOFs and polymers from the huge material database, high‐throughput computing screening (HTCS) and machine learning (ML) techniques have been used to accelerate the screening and performance evaluation of MMMs. For example, Wilmer et al. utilized HTCS to predict the performance over a million MMMs, and conducted a 12‐fold individual optimization of the techno‐economic evaluation of the three‐stage capture process for MMM membranes.^[^ ^18^ ^]^ Aydin et al.^[^ ^19^ ^]^ calculated the permeability and selectivity of 18 different gases in covalent organic frameworks/polymer MMMs using HTCS methods, indicating that 25 polymers can exceed the upper bound.^[^ ^20^ ^]^ Keskin et al.^[^ ^21^ ^]^ utilized HTCS to identify high‐performance MMM for O_2_/N_2_ separation, and found that MMMs composed of covalent organic framworks as fillers outperformed traditional filler (such as zeolites) MMMs in terms of O_2_ permeability and O_2_/N_2_ selectivity. And Ayda et al.^[^ ^22^ ^]^ incorporated MOFs into polymers then the H_2_ permeation rate of the membrane was increased by two times and the selectivity of H_2_/N_2_ was also slightly enhanced. As for the ML method, it is an excellent approach to analyzing a large amount of simulated material data. In the past several years, ML algorithms have been used to study MOFs for various adsorption‐based gas separations (such as CO_2_ capture).^[^ ^7^, ^23^ ^]^ Zhou et al.^[^ ^24^ ^]^ used different ML algorithms to predict the D_2_/H_2_ selectivity of MOF membranes at infinite dilution, 77 K, and found that the D_2_/H_2_ membrane selectivity of the best MOFs is one order of magnitude higher than those previously reported. Daglar et al.^[^ ^25^ ^]^ calculated the permeability and selectivity of a total of 5249 MOF membranes and 31494 different MOF/polymer MMMs by utilizing ML methods. Additionally, the transferability of the ML models was investigated on an unseen computationally generated hypothetical MOFs dataset. The HTCS method can quickly screen membrane materials with high CO_2_ separation performance, thereby shortening the research and development cycle and reducing the costs.^[^ ^26^ ^]^ And the ML methods can establish efficient and accurate predictive models, providing support and guidance for the design and understanding of new materials.^[^ ^27^ ^]^

In this work, we explored the relationships between the structure and properties of MMMs composed of different polymer matrices and MOFs for CO_2_ separation, providing guidance for designing high‐performance MMM. The CO_2_ permeability (*P*) and selectivity (*S*) of MOFs were computed using the HTCS method, and a dataset incorporating the trade‐off between selectivity and permeability (*TSP*) properties was established based on Maxwell's equations. Then different ML models were trained to predict the *TSP* performance of MMM. Compared to other works, this work introduced polymer feature data as innovative input for model training and the Stacking algorithm was employed to improve the predictive performance of traditional models. Finally, the generalization and extrapolation ability of the model was studied by exploring the relationship between gas properties and feature descriptors (multivariate linear extrapolation) and testing the model across different gas separations and MMMs (transfer learning). An interactive desktop software was developed based on the best‐performing model to rapidly and accurately predict the CO_2_ separation performance of MMMs.

## 2 Methods

### 2.1 Molecular Simulations

The grand canonical Monte Carlo (GCMC) and molecular dynamics (MD) simulation techniques were used to calculate the adsorption capacity (*N*) and diffusion coefficient (*D*) of 4 binary CO_2_ mixtures (CO_2_/X, X = CH_4_, N_2_, H_2_, O_2_) in MOFs at 298 K and 10 bar. Independent GCMC and MD simulations were performed for each MOF, and the Lorentz‐Berthelot rule^[^ ^28^ ^]^ was used to calculate the interactions between MOFs and adsorbate molecules. To simulate the 3D extension of the unit cell, periodic boundary conditions were adopted, and 3D directions were ensured to be greater than 24 Å. A cutoff radius of 12 Å was taken, and the interactions between MOF and gas molecules, as well as the electrostatic interactions between gas molecules, were calculated using Ewald summation.^[^ ^29^ ^]^ In each MOF, GCMC simulations were performed for 1000000 cycles, with the first 500000 cycles used for equilibrating the simulation system and the last 500000 cycles used to calculate the average swing. The MD simulation time for each MOF was 10 ns, with the final 5 ns used for production. All GCMC and MD simulations were performed using the RASPA^[^ ^30^ ^]^ software package.

### 2.2 Mixed Matrix Membranes

In this work, MMM refers to advanced composite materials composed of polymers as the matrix and MOFs as the embedded inorganic particles. For the MOF part, the Computation‐Ready, Experimental MOFs (CoRE‐MOFs) database reported by Chung et al.^[^ ^31^ ^]^ was employed. For the polymer component, based on the performance of reported polymers and their combination with MOFs, nine polymers with high, medium, and low permeability (Table S4, Supporting Information) were chosen: two polymers with intrinsic microporosity (PIM‐1,^[^ ^32^ ^]^ PIM‐7^[^ ^31^ ^]^), amorphous plasma electrolytic oxidation (a‐PEO),^[^ ^33^ ^]^ semi‐crystalline plasma electrolytic oxidation (s‐PEO), polyphenylene oxide (PPO),^[^ ^34^ ^]^ cellulose acetate (CA),^[^ ^35^ ^]^ and three polyimides (Matrimid, PI‐3, and PI‐5). 6013 CoRE‐MOFs and these nine polymers were combined in different volume fractions (0.1, 0.2, 0.3) to create 162351 different MMMs. And the performance of MMMs was calculated by the Maxwell's equations (Details are in section S3 of the Supporting Information). These MMMs were assumed to be assembled under ideal circumstances and the MOFs were uniformly distributed in polymer matrix. Then the CO_2_ separation performance of MMMs toward four binary mixtures CO_2_/X (X = CH_4_, N_2_, H_2_, O_2_) (*v*/*v* = 1/1) at 298 K and 10 bar was analyzed by Maxwell's equations (Details are in section S3 of the Supporting Information), which was also used for subsequent ML model training.

By integrating Maxwell's equations with machine learning, our hybrid approach leverages the precision of physics‐based modeling with the scalability and predictive power of data‐driven techniques, thereby enhancing the efficiency and accuracy of MMM performance prediction while mitigating the risk of secondary errors through continuous model refinement.

### 2.3 Descriptors of Mixed Matrix Membranes

To enhance the generalizability of the model and better establish the relationship between the MMM structures and performances, we constructed a set of descriptors for model training. Ideally, descriptors should be easy to obtain and calculate, have low dimensionality, and be related to the output properties. We extracted eleven different features as potential descriptors and divided them into five groups (**Table** **1**). Group A consists of three features on polymer matrices: fractional free volume (FFV), density (ρ_*poly*_), and the density ratio between MOF and polymer (Table S4, Supporting Information). Group B includes pore characteristics of MOFs: the largest cavity diameter (LCD, Å), pore limit diameter (PLD, Å), and their ratio (LCD/PLD), which were calculated using the Zeo++ package.^[^ ^36^ ^]^ Group C represents the geometric shape of MOF pores, including volumetric surface area (VSA), porosity (*φ*), density (*ρ*), and pore size distribution percentage (PSD%) between 2.5 and 3.5 Å. We calculated the VSA and *φ* using N_2_ with a diameter of 3.64 Å and He with a diameter of 2.58 Å as probes in the RASPA software package.^[^ ^30^ ^]^ PSD% is one of the structure parameters of MOFs, indicating the proportion of pores with sizes between 2.5 and 3.5 Å in the overall pores,^[^ ^37^ ^]^ the equation of the PSD% was listed in the Supporting Information. Group D are energy descriptors, representing the interaction strength between gas molecules and MOFs, such as the heat of adsorption (Q_st_) of CO_2_ gas on the MOF. We vertically arranged the eleven descriptors for the nine types of MMMs and processed the data to obtain 54000 samples for model training in ML.

**Table 1:** Descriptors that are used to construct a feature vector for ML models.

Group	Feature	Symbol
A	Polymer density (kg/m^3^)	*ρ*_poly_
	Fractional free volume (cm^3^/g)	FFV
	Density ratio	*ρ*_(MOF/poly)_
B	Largest cavity diameter (Å)	LCD
	Pore limiting diameter (Å)	PLD
	Pore size ratio	LCD/PLD
C	Density (kg/m^3^)	*ρ*
	Volumetric surface area (m^2^/cm^3^)	VSA
	Porosity	*φ*
	Pore size distribution	PSD%
D	Heat of adsorption (kJ/mol)	*Q*_st_

The features are divided into 4 groups. Groups A, B, C, and D represent the features about polymer characteristics, MOF pore size, MOF pore geometry, and MOF energy descriptors, respectively.

### 2.4 ML Algorithms

In this work, the CO_2_ separation performance of MMMs was predicted by 6 ML algorithms (Backpropagation Neural Network (BPNN), Random Forest (RF), Extreme Gradient Boosting (XGB), Extremely Randomized Trees (ET), K‐Nearest Neighbor (KNN), and Gradient Boosting Decision Trees (GBDT)) and then a stacked ensemble regression using stacking fusion prediction is further employed. Stacking Ensemble Regression^[^ ^38^ ^]^ (Stacking) was employed to learn and to integrate the predictions using the meta‐model, thereby improving the overall performance and robustness of the model. More details and the parameters of the 6 ML algorithms and the stacking algorithm are listed in section S4 of Supporting Information.

The ML models are built by training it on a dataset that consists of target attributes and preprocessed features. After preprocessing the data, we used 11 multivariate statistical features to construct the model for predicting the *P*_CO__2_ and *TSP* of MMMs. To improve the stability and accuracy of the model, *k*‐fold cross‐validation was employed.^[^ ^39^ ^]^ In this work, a value of *k* = 5 was chosen, and the cross‐validation process was repeated 5 times (repeated fivefold cross‐validation). Root Mean Square Error (RMSE), Mean Absolute Error (MAE), and the coefficient of determination (*R* ^2^) were used as evaluation metrics for the ML models. We reiterate that there is no shared MMMs between the training set and the test set during any model training. Then, Shapley Additive Explanation (SHAP)^[^ ^40^ ^]^ was combined with trained models to explain the importance of different predictive factors on the performance. This strategy is based on game theory.^[^ ^41^ ^]^ To compute SHAP values based on tree models, we utilized an algorithm called TreeExplainer.^[^ ^42^ ^]^ Compared to the default importance calculation methods of the model, SHAP technology allows for quantifying the influence of features on model output in terms of magnitude (significant or insignificant) and direction (positive or negative). The Python library for the calculation of SHAP came from the Lundberg et al’s^[^ ^42^ ^]^ work. Despite the limitation that SHAP analysis can only explain the dataset used for modeling, the broad applicability of our findings is supported by the diversity of the dataset, alignment with established chemical principles, the model robust extrapolation capabilities, and rigorous validation strategies. Consequently, our machine learning model does offer general guidance for the preparation of MMMs, with high confidence in its predictive outcomes.

## 3 Result and Discussion

### 3.1 MOF Feature Correlation and Univariate Analysis

As MOFs is an important part of MMMs, we initially focused on exploring the MOF Feature Correlation. First, the correlations between various MOF descriptors were investigated (Figure S10, Supporting Information). PLD and LCD, as well as VSA and *φ*, exhibit a strong positive correlation because they are associated with pore size and pore volume, respectively. Q_st_, a parameter that describes the strength of interaction between gas molecules and MOFs, demonstrates a negative correlation with PLD, LCD, VSA, and *φ* as pore size and pore volume decrease, the interaction becomes stronger.

**Figure 1:**
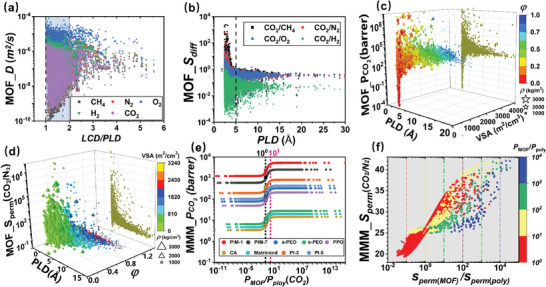
The relationships between performance indicators and different descriptors. a) CH_4_, N_2_, O_2_, H_2_, CO_2_ diffusivity of MOF and LCD/PLD. b) The gas diffusion selectivity in MOF and PLD. c) *P*_CO__2_ and PLD, VSA, *ρ*, *φ*. The size and color of the star represents the value of *ρ* and *φ*. d) *S*_perm_ (CO_2_/N_2_) and PLD, VSA, *ρ*, *φ*. The size and color of the tetrahedron represents the value of *ρ* and *VSA*. e) *P*_CO__2_ of MMM and *P*_MOF_/*P*_poly_. The squares represent the *P*_CO__2_ of different polymers. The colored dots represent the *P*_CO__2_ of different MMMs. f) *S_perm (_*_MOF)_/*S_perm(_*_poly)_, *P*_MOF_/*P*_poly_, and *S_perm_* (CO_2_/N_2_) of MMM based on the PIM‐1 polymer, and the color bar represents the *P*_MOF_/*P*_poly_.

Then we investigated the structure‐property relationships of MOF by univariate analysis. For the gas diffusion, as shown in **Figure** **1**a, the diffusion of CO_2_ in MOFs is significantly lower. The Henry's constant of CO_2_ is also an order of magnitude larger than that of other gases (Figure S11a, Supporting Information). This can be attributed to the quadrupole moment (Qua) of CO_2_ molecules,^[^ ^43^ ^]^ which leads to stronger Coulomb interactions with MOFs and will hinder diffusion. The ratio of LCD to PLD (LCD/PLD) can represent the pore morphology of MOFs, where LCD/PLD = 1 indicates a uniform pore size and LCD/PLD > 1 indicates the presence of larger cavities and narrow channels.^[^ ^44^ ^]^ When the LCD/PLD = 1 ∼ 2, the D of all five gases is high, with the maximum value observed at a ratio of 1. This indicates that the gas diffusion is benefited by the uniformity of cavity and channel sizes. When the LCD/PLD > 2, it indicates the presence of large cavities in the MOF that exceed twice the diameter of the molecules, and gas diffusion in these cavities is mainly influenced by the pore walls. Thus, D shows a slight decrease and eventually reaches a stable state. This observation aligns with previous findings.^[^ ^18^ ^]^ Figure 1b illustrates the relationship between diffusion selectivity (*S*_diff_) of CO_2_/X (X = CH_4_, N_2_, O_2_, H_2_) and PLD. When PLD < 5 Å, high *S*_diff_ can be obtained, and *S*_diff_ gradually decreases with increasing PLD. This is because when PLD is large, the interaction between gas and the pore wall is weak, resulting in poor separation performance. Additionally, when the LCD is ≈ 4–6 Å, both the Henry coefficient and adsorption selectivity values reach their peak, indicating that the adsorbate can interact maximally with the pore wall^[^ ^45^ ^]^ (Figure S11, Supporting Information).

Next, we investigated the relationship between structural descriptors and the permeability and permselectivity (*S*_perm_) of MOFs, which are two important indexes to evaluate the membrane separation performance. Permeability is determined by the adsorption and diffusion performance. Selectivity is controlled by the affinity between the gas components, the membrane surface, pore size of the membrane, and its permeability. In Figure 1c, *P*_CO__2_ shows a sharp increase with the increase of VSA, PLD, and *φ*, reaching a peak before gradually stabilizing. Due to the high correlation between PLD and LCD, a similar trend is observed between LCD and *S_perm_* (Figure S12, Supporting Information). However, the relationship between *ρ* and *P*_CO__2_ is opposite, with an increase in *ρ* leads to a decrease in *P*_CO__2_. For *S*_perm_, as PLD, LCD, VSA, and *φ* increases, it first decreases sharply and then stabilizes (Figure 1d and Figure S12a–c, Supporting Information). This can be attributed to the influence of pore size and porosity on the molecular permeation behavior.^[^ ^46^ ^]^ Smaller pore sizes and lower porosities can restrict the passage of larger molecules, thereby enhancing the permeation selectivity.

### 3.2 Performance of Mixed Matrix Membranes

To validate the accuracy of the method using Maxwell's model, the theoretical permeability for five gases(H_2_, CO_2_, O_2_, N_2_ and CH_4_)in the MMM based on MOF‐5 (at different volume fractions: 0.1, 0.2, 0.3) and the polymer Matrimid was calculated, and then compared to the experimental permeability.^[^ ^47^ ^]^ As shown in Figure S13 (Supporting Information), the calculated permeability matched well with the experimental value, demonstrating the high accuracy of the method. In the following study, the volume fraction of the MOF was set as 0.2. Then, the gas permeability in MOFs were calculated and the experimental gas permeability in nine different polymers were collected (Table S4, Supporting Information). By utilizing the Maxwell model, the gas permeation performance of 54117 MMM materials can be obtained.

Figure 1e shows that when the *P*_MOF_ < *P*_poly_, *P*_MMM_ < *P*_poly_. However, when the *P*_MOF_ > *P*_poly_, *P*_MMM_ increases. When the *P*_MOF_ ≈10 × *P*_poly_, the *P*_MMM_ reaches its maximum and then keep constant, similar to the findings of E. Drioli et al.^[^ ^48^ ^]^ This indicates that the addition of highly porous MOF can enhance the permeation performance of MMM. Therefore, to achieve high‐performance MMM, polymers with high permeability and MOF with permeability exceeds ten times that of the pure polymer should be selected. Figure 1f illustrates that when the *S*_perm(MOF)_/*S*_perm(poly)_ increases, the MMM_*S*_perm_(CO_2_/N_2_) increases until it reaches a peak and stabilizes. When the *P*_MOF_/*P*_poly_ = 1–10 (the red data points), the MMM_*S*_perm_(CO_2_/N_2_) increases rapidly but with a lower peak value. When *P*_MOF_/*P*_poly_ > 10, the MMM_*S*_perm_(CO_2_/N_2_) increases slowly but reaches a higher peak with *S*_perm(MOF)_/*S*_perm(poly)_ > 10^3^.Therefore, combinations with *P*_MOF_/*P*_poly_ > 10 and *S*_perm(MOF)_/*S*_perm(poly)_ > 10^3^ will facilitate the design of MMM with outstanding performance.

### 3.3 Machine Learning

#### 3.3.1 ML Model

**Figure 2:**
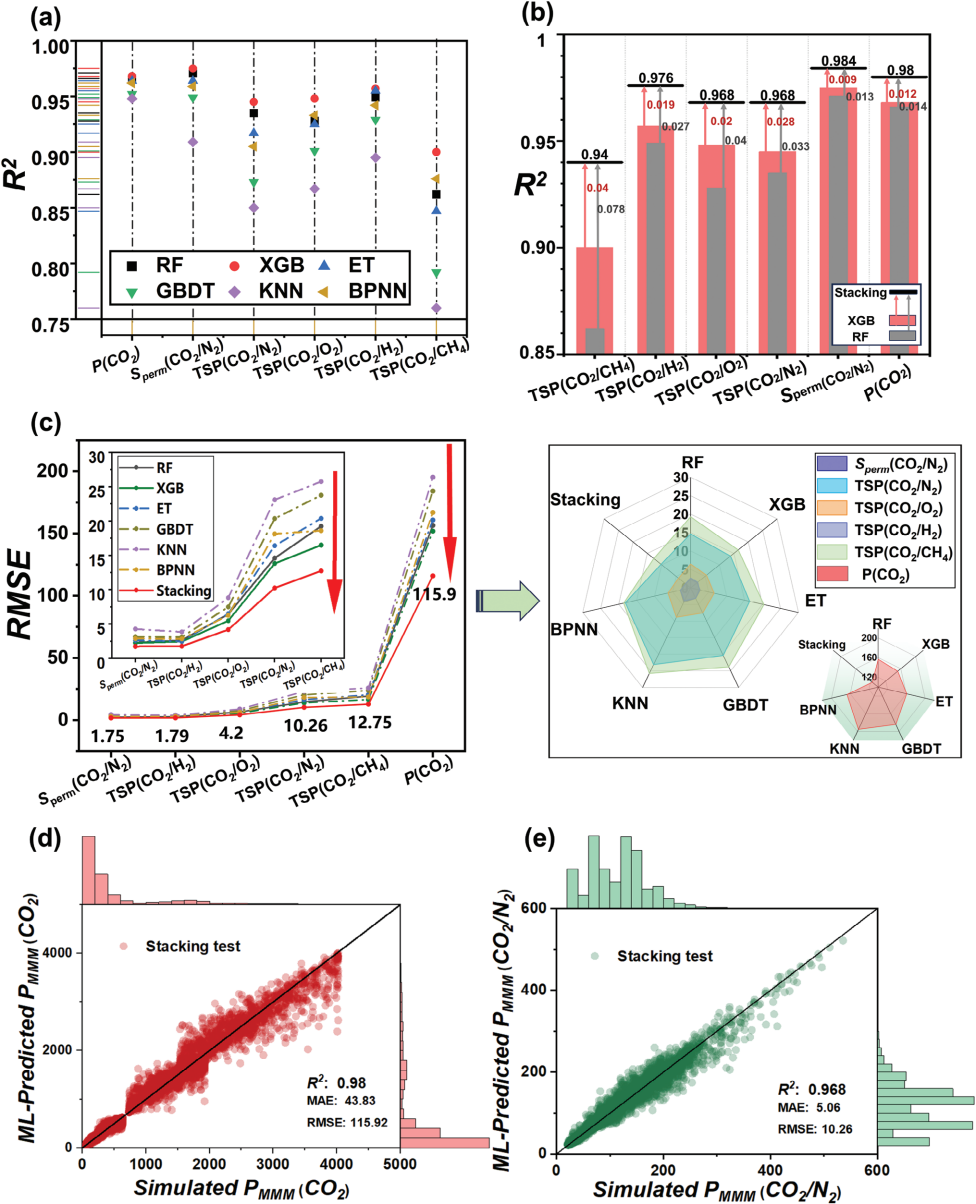
ML for CO_2_ separation performance of MMMs. a) *R^2^* of six ML algorithms for the prediction of different performance indicators. b) *R^2^* of the stacking algorithm and the base models (RF and XGB). c) RMSE of seven algorithms for predicting different performance indicators. The inset in the left graph is an enlarged image of the RMSE results for the first five performance predictions, and the right graph illustrates the RMSE distribution using radar chart.  Comparison of predicted and simulated results for d) *P_MMM_* (CO_2_) and e) *TSP(CO_2_/N_2_)* in MMM using stacking algorithm.

For the complex structures‐performance of 54117 MMMs concerning four different CO_2_ mixtures, conventional univariate analysis is insufficient for quantitative analysis. Therefore, the ML technique was employed to perform the big data analysis of MOFs and membranes performance.^[^ ^49^ ^]^ Six ML algorithms were trained for predicting the CO_2_ separation performance of MMMs. For detailed training parameters, please refer to Section S4 and Table S5 (Supporting Information). The predictive results are presented in **Figure** **2**a and Table S5 (Supporting Information). The XGB and RF model perform the best, achieving *R^2^* exceeding 0.9 for the test sets of *P*_CO__2_ and *TSP*. This can be attributed to the distinct tree structures and algorithmic characteristics of these two models. RF reduces overfitting and compensates for individual decision tree errors by combining the predictions of multiple decision trees. It also exhibits robustness against noise and outliers. XGB improves predictive performance and generalization ability through iterative training and advanced regularization techniques. Thus, both models excel in capturing the relationship between MMM descriptors and performance.

#### 3.3.2 Stacking Ensemble Regression

To further enhance the prediction accuracy and generalization ability of the models, the Stacking algorithm^[^ ^38^ ^]^ was introduced. It combines two or more models as base models utilize the predictions of the base models as inputs for the meta‐model (Figure S9, Supporting Information). We employed RF and XGB as the base models and stacked them using the training principle of XGB, obtaining a new Stacking model for predicting the performance of MMM. As shown in Figure 2b,c, the Stacking model demonstrates improved *R^2^* and lower RMSE for various performance metrics of MMM, particularly achieving a remarkable enhancement in predicting *TSP*(CO_2_/CH_4_) with *R* ^2^ of 0.94. The predicted TSP and *P*_CO__2_ obtained from the Stacking model match well with the simulated results (Figure 2d,e; Figure S20, Supporting Information), demonstrating its good prediction accuracy. And the Stacking model also exhibits remarkable robustness and strong generalization ability, which can be particularly highlighted in future applications involving transfer learning.

Additionally, the performance of the Stacking model based on different feature groups (Groups A, B, C, and D in Table 1) were studied. The results are presented in **Table** **2**. When feature group A, which represents polymer features, was used, the *R^2^* on the test set reached a maximum of 0.87. This good accuracy can be attributed to the high importance of polymer features. However, the high error rate indicates the presence of overfitting and the insufficiency of the input data samples. When the feature group B, which are pore size descriptors, was added, the *R^2^* increased significantly, indicating the importance of pore size. And the *R^2^* slightly improved with the addition of the feature groups C and D. Therefore, in the absence of other MMM data, this study suggests that using the feature group of polymer features and MOF pore descriptors (A+B) can yield relatively good prediction results.

**Table 2:** The selection of descriptors group for Stacking model.

Performance Indicators	Descriptor	Train			Test		
	Groups	*R^2^*	*MAE*	*RMSE*	*R^2^*	*MAE*	*RMSE*
*P*CO_2_	A	0.903	114.586	260.395	0.905	114.880	260.505
	A+B	0.976	48.330	129.600	0.963	62.650	158.870
	A+B+C	0.987	34.190	93.030	0.976	46.920	127.500
	A+B+C+D	0.989	32.670	88.040	0.980	43.770	115.770
*S_perm_* (CO_2_/N_2_)	A	0.880	2.499	4.877	0.883	2.446	4.675
	A+B	0.986	0.765	1.630	0.974	1.119	2.259
	A+B+C	0.990	0.614	1.344	0.981	0.909	1.899
	A+B+C+D	0.992	0.591	1.290	0.984	0.865	1.757
*TSP*(CO_2_/N_2_)	A	0.720	14.560	30.310	0.730	14.30	29.720
	A+B	0.970	4.550	9.968	0.945	6.766	13.571
	A+B+C	0.980	3.540	7.974	0.962	5.376	11.136
	A+B+C+D	0.986	3.410	7.736	0.968	5.062	10.226
*TSP*(CO_2_/O_2_)	A	0.826	5.056	10.116	0.832	5.085	9.949
	A+B	0.950	2.450	5.610	0.945	2.835	5.664
	A+B+C	0.967	1.810	4.38	0.962	2.284	4.686
	A+B+C+D	0.978	1.680	3.970	0.970	2.100	4.206
*TSP*(CO_2_/H_2_)	A	0.870	2.380	4.260	0.870	2.340	4.200
	A+B	0.968	1.010	2.220	0.963	1.220	2.300
	A+B+C	0.980	0.770	1.729	0.975	0.967	1.901
	A+B+C+D	0.992	0.725	1.667	0.977	0.922	1.806
*TSP*(CO_2_/CH_4_)	A	0.540	17.860	36.040	0.555	17.330	34.570
	A+B	0.930	6.630	14.205	0.900	8.700	16.740
	A+B+C	0.958	4.807	10.717	0.934	6.806	13.606
	A+B+C+D	0.963	4.609	10.026	0.942	6.538	12.749

### 3.4 Big Data Mining

#### 3.4.1 Feature Importance

**Figure 3:**
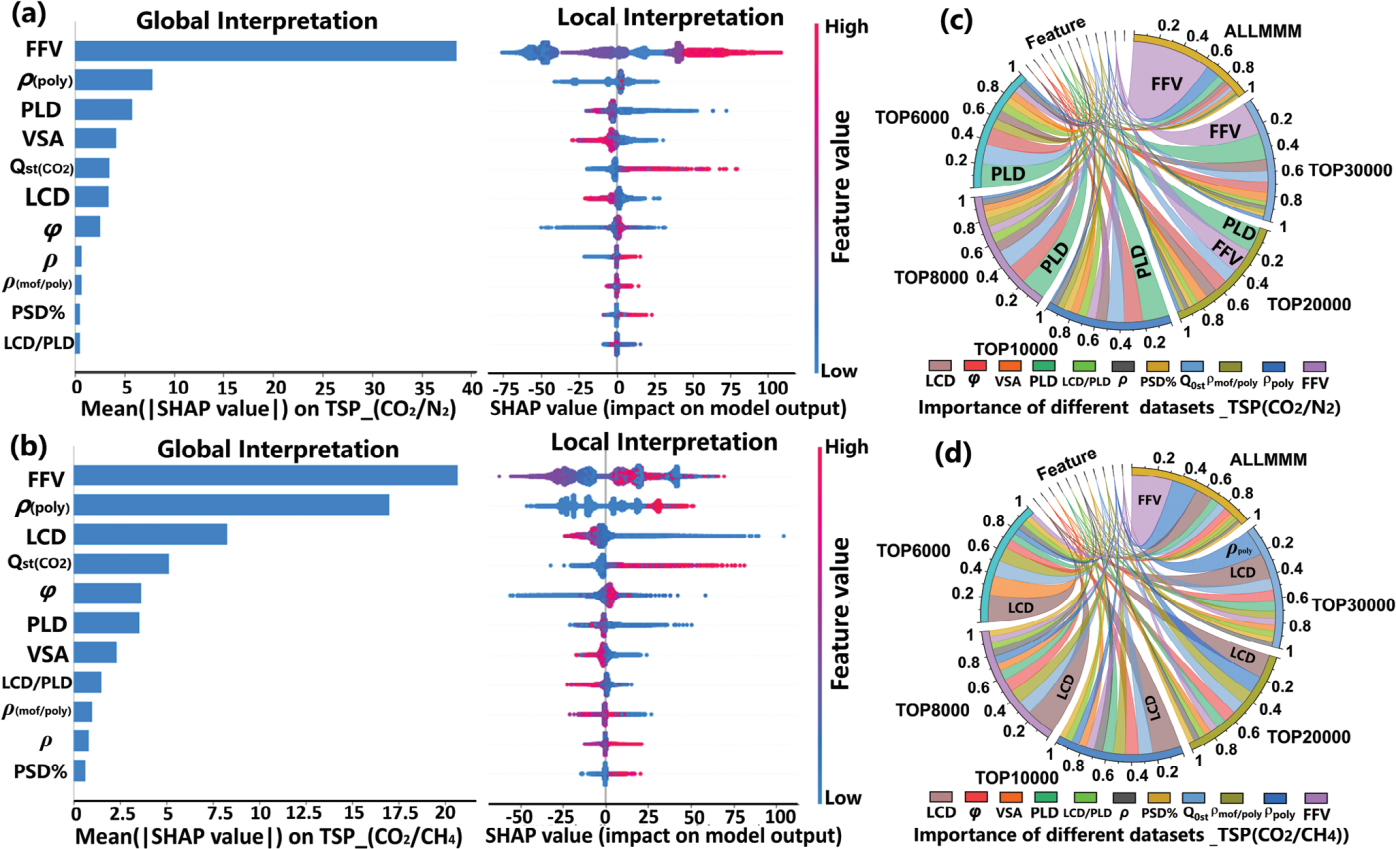
Relative importance analysis of features to MMM performance based on the RF model. The global feature importance (left, bar chart) and local explanation plot (right, bee swarm plot) for predicting a) *TSP*(CO_2_/N_2_) and b) *TSP*(CO_2_/CH_4_). Each point represents an individual data point, and multiple points located at the same x position indicate density. The color represents the feature value, with red indicating high and blue indicating low values. Positive/negative SHAP values indicate positive/negative feature impact on predictions. The relative importance of features for predicting c) *TSP*(CO_2_/N_2_) and d) *TSP*(CO_2_/CH_4_) using different TOPMMMs datasets. Different color represents different feature, and the feature importance rankings for each dataset are arranged clockwise. The features with the highest proportion of importance within that dataset are marked in the graph.

After obtaining the optimal models, we utilized the tree‐based explainer in SHAP to interpret and mine the relationship between the descriptors and the target performance. As the input for the Stacking model is the pre‐trained results of the base models, it cannot be directly used for feature analysis. When the predictive performance of two base models is similar, the training and interpretation processes of RF are generally faster compared to XGB, especially when dealing with large datasets or a high number of features. Therefore, the SHAP values for predicting *TSP*(CO_2_/N_2_) based on the RF model are presented (**Figure** **3**a,b). The bar charts on the left represent the extent of influence on the model target performance, with the average absolute SHAP values (|SHAP value|) across all points in the dataset (Tables S7 and S8, Supporting Information). The swarm plots on the right show the SHAP distributions for all MMMs. The results indicate that the descriptor FFV and *ρ*_poly_ contribute the most, consistent with the work by Lan et al.^[^ ^50^ ^]^ Similar results are obtained for the other three *TSPs* (Tables S8 and S9, Supporting Information). It is because the polymer features play a basic role in the gas permeability and selectivity of MMMs. A smaller FFV can enhance selectivity by restricting the passage of larger molecules while a larger FFV may increase permeability by providing more channels and pores for gas molecules to permeate. And the *ρ*_poly_ can influence the intermolecular interaction forces and the molecular sieving behavior.^[^ ^31^, ^51^ ^]^ For the MOF features, the importance ranking for TSP of CO_2_/X (N_2_, CH_4_, O_2_, H_2_) is as follows: PLD > LCD > VSA > Q_st_ (Figure S21a,b, Supporting Information). It is worth noting that the top two features (PLD and LCD) are related to pore size, indicating that the suitable pore size of MOFs is crucial for membrane separation performance.^[^ ^52^ ^]^

To further explore the common features of outstanding MMM materials, we rank the performance of MMMs based on the TSP value for the four CO_2_/X systems and divided the MMM database into six datasets: TOP 6000, TOP 8000, TOP 10000, TOP 20000, TOP 30000, and ALL MMMs (Table S11, Supporting Information). The relative importance of features to the MMM performance in different datasets was evaluated, as shown in Figure 3c,d and Figure S21c,d (Supporting Information). The feature importance of the ALL MMM dataset aligns with the global SHAP value explanations. And the importance of polymer features shows a decreasing trend from the ALL MMM dataset to the TOP 6000 dataset. This is attributed to the reduced diversity of polymer types in outstanding MMMs, such as there were only three polymer species in TOP 6000 for CO_2_/N_2_ performance, resulting in a narrower range of features and a decrease in their importance. The importance of MOF features, particularly PLD and LCD, exhibits an increasing trend. For CO_2_/H_2_, the importance of PLD increases from 2.0% to 31.8%. And in the TOP 6000 dataset, PLD and LCD are more important than FFV and *ρ*_poly_ for the *TSP* performance of the MMM (Tables S11–S14, Supporting Information). This indicates that when the performance of MMMs reaches a certain level, incorporating or replacing MOFs with better performance and more suitable pore characteristics is an effective approach to further enhance MMM separation performance, rather than replacing the polymer.

#### 3.4.2 Top‐Performing MMMs and MOFs for CO2 Separation

**Figure 4:**
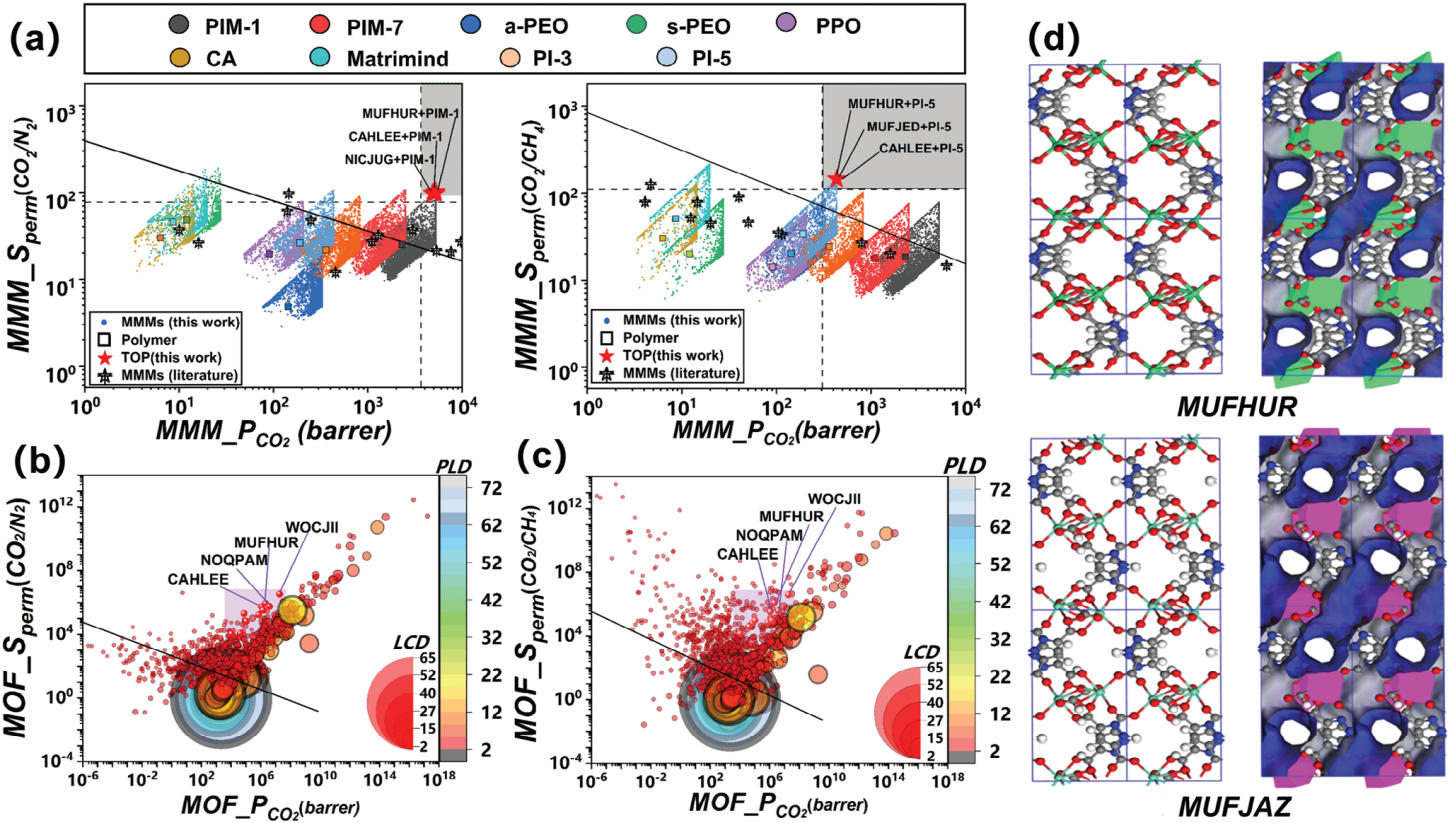
Optimal MMMs and MOFs for CO_2_ separation. a) Relationship between *S*_perm_ (CO_2_/N_2_) or *S*_perm_ (CO_2_/CH_4_) and *P*_CO__2_ in MMM. The black line represents the Robeson upper bound. The properties of neat polymer are marked with square symbols, while the predicted properties of MMMs derived from the polymer are marked with circular symbols of the same color. Red stars represent the TOP 10 MMMs for CO_2_ separation, and black stars with black borders represent the reported performance of MMMs in the literature. Relationship between b) *S*_perm_ (CO_2_/N_2_) or c) *S*_perm_ (CO_2_/CH_4_) and *P*_CO__2_ in MOF. The black line represents the Robeson upper bound. The purple region indicates the distribution of the TOP 10 MOFs. d) Atomic structures and pore simulation structures of the top MOFs MUFHUR and MUFJAZ applicable to the four gas separation systems.

**Figure** **4**a shows that a number of MMMs formed by high‐permeability polymers and MOFs can surpass the Robeson limit,^[^ ^48^ ^]^ and the TOP 10 MMMs were identified based on the TSP value (the red stars). They exhibit the *P*_CO__2_ and *S_perm_* ≈100 times and 5 times higher than the Robeson limit.^[^ ^48^ ^]^ For CO_2_/N_2_ separation, MMMs combining PIM‐1 with MOFs exhibit significantly higher performance, similar to the findings of Chen et al.^[^ ^53^ ^]^ It can be attributed to the high porosity and the aromatic ether linkages of PIM‐1^[^ ^54^ ^]^ which exhibit favorable interactions with CO_2_, enhancing the CO_2_/N_2_ separation performance. MMMs composed of PI‐5 and MOFs are effective in separating CO_2_/CH_4_. The aromatic structure in polyimides interacts with the organic ligands of the MOF, which can improve the compatibility between PI‐5 and MOFs and minimize undesired interface voids,^[^ ^55^ ^]^ thus selectivity and permeability for CO_2_/CH_4_ separation are enhanced. And MMMs combining a‐PEO with MOFs are suitable for CO_2_/O_2_ and CO_2_/H_2_ separation (Figure S22, Supporting Information) due to the inherent dipole‐quadrupole interactions between the epoxy ethylene oxide units in a‐PEO and CO_2_ molecules.^[^ ^56^, ^57^ ^]^ The performance of the top MMMs identified in this study exceeds the values reported in the literature (Tables S16 and S18, Supporting Information). The polymers used in these MMMs are all well‐established and synthesized materials, further demonstrating the potential application of these MMMs in CO_2_ capture and separation.

Additionally, based on the performance of MMMs, the TOP 10 MOFs were selected (Figure 4b,c; Table S19, Supporting Information). The purple region represents the MOFs with both the CO_2_ permeability and selectivity exceeding the Robeson upper bound. The structural diagrams of the top 10 MOFs (Figure 4d; Figure S24, Supporting Information) show that all of them possess dense and regular pore structures. The dense pore structure is advantageous for enhancing gas permeation per unit area, while the regular and uniform pore structure (LCD/PLD = 1∼2) contributes to improved gas separation performance by reducing non‐selective permeation behavior. Similar findings have been reported in previous studies.^[^ ^37^ ^]^

### 3.5 Application (Prediction and Extrapolation)

To study its performance for predicting the CO_2_ permeation separation performance of other MMM materials beyond the scope of this work, the model was tested by two extrapolation methods: multivariate regression extrapolation^[^ ^58^ ^]^ and transfer learning of ML models.^[^ ^59^ ^]^

#### 3.5.1 Multivariate Regression Extrapolation

**Figure 5:**
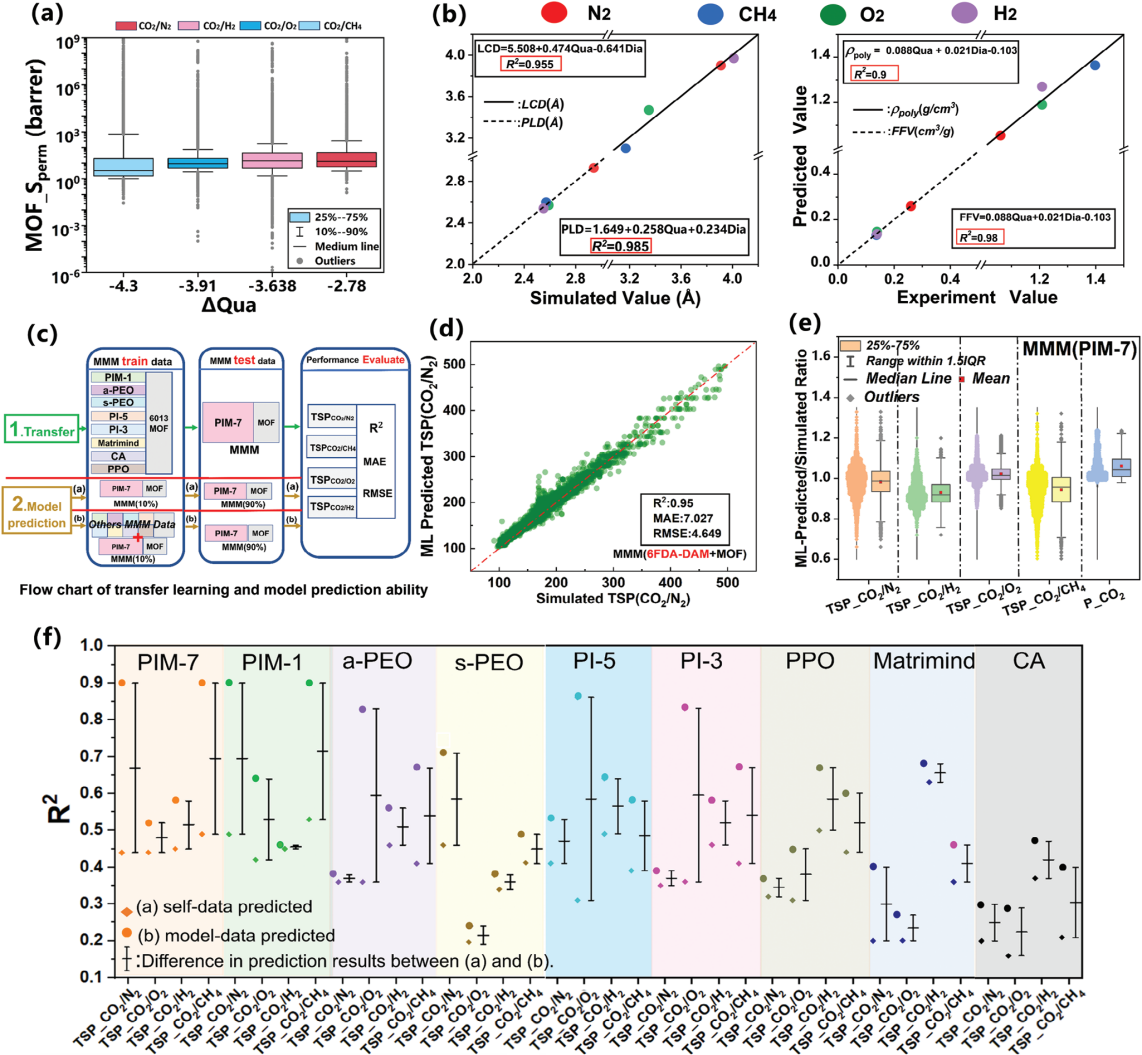
a) The relationship between *S_perm_* and *∆Qua* of different gas mixtures in 6013 CoRE‐MOFs. b) Left: Comparison of the predicted PLD and LCD by the multivariate regression model and the simulated PLD and LCD. Right: Comparison of the predicted FFV and *ρ*_poly_ by the multivariate regression model and the experimental FFV and *ρ*_poly_. c) Diagram illustrating the data partition for transfer learning and model transfer learning capability evaluation. d) Prediction results of transfer learning for MMM (6FDA‐DAM) using the stacking model. e) The ratio of transfer prediction results of various performance metrics for MMM (PIM‐7) using the stacking model to the simulated results. The left side is the distribution of ratio data and the right side is the boxplot (red squares represent the mean values). f) Based on the a)self‐data, and b)model‐data, training strategies, the evaluation results of the transfer learning ability of nine different polymers in MMM. Left: the R^2^ values for different training strategies, Right: the difference in comparison between the two results. Different colors represent different MMMs with polymer, and the shape of the dots represents different training strategies.

Based on the previous univariate analysis, gas properties also impact the performance of MOFs. Therefore, the relationship between the difference in gas quadrupole moment (∆Qua), the difference in gas kinetic diameter (∆Dia), the difference in gas polarizability (∆Pol), *S_diff_*_(MOF),_ and *S_perm_*_(MOF)_ was investigated. From **Figure** **5**a, as ∆Qua increase, both the median and mean values of *S_perm (MOF)_* increase. ∆Dia, ∆Pol, and *S*_diff(MOF)_ exhibit similar trends (Figure S25, Supporting Information), as mentioned in previous study.^[^ ^60^ ^]^ For the use of a simple formula for predicting and extrapolating the feature performances of new materials, we gradually incorporated parameters such as Qua and Dia into the linear regression model to enhance its predictive accuracy. The multivariate regression models for predicting PLD, LCD, FFV, and polymer density (*ρ*_poly_) achieved an R^2^ value of above 0.9, as depicted in Figure 5b. Furthermore, the model was applied to the CO_2_/C_2_H_2_ system beyond the original dataset, the optimal PLD (3.23 Å) was predicted, which aligns with the experimental results reported (3.2 Å) by Chen et al.^[^ ^61^ ^]^ However, the predictions for other feature variables (*ρ*, *φ*) (Figures S27–S31, Supporting Information) were unsatisfactory, with R^2^ values below 0.6. Therefore, this work further employs transfer learning methods from the field of data mining to achieve directed prediction and rational design of the optimal MMMs for various CO_2_ binary separation systems.

#### 3.5.2 Transfer Learning

The most useful ML models can learn from existing materials data, and generalize to new materials to assist the design and discovery of new materials. First, our trained Stacking model was utilized to predict the performance of a novel type of MMMs composed of the polymer 6FDA‐DAM and MOFs. The ML predictions demonstrated an *R^2^* value of 0.95 when compared to the results obtained from Maxwell calculations, as shown in Figure 5d. This validates the feasibility of the extrapolation strategy and highlights the potential of utilizing big data mining techniques for investigating the CO_2_ separation performance of MMMs. Researchers can obtain the CO_2_ separation performance of MMM materials individually or in bulk by simply inputting the parameters into our interactive desktop application developed based on the Stacking model (refer to Figure S32, Supporting Information, accessible from the GitHub repository at https://github.com/haowanae/Pred_MMM_CO2/tree/master.

In order to gain a deeper understanding of the Stacking model generalization capability for different CO_2_ separation performance of MMMs, we employed transfer learning using existing datasets. We split our dataset by the types of polymer matrix (Transfer section, Figure 5c) and hold one type out as an “unseen” polymer material for the model. Figure S33a (Supporting Information) demonstrates the transfer learning targeting 6000 MMM(PIM‐7) composed of the polymer PIM‐7 and MOF. The model achieved an *R^2^* value of 0.9 for predicting the TSP(CO_2_/N_2_) of MMM (PIM‐7), and the *R^2^* for other systems also exceeded 0.87 (Table S21, Supporting Information). The distribution of the ratio data between predicted and simulated values (Figure 5e) reveals that the mean ratios fall within the range of 0.9–1.1, validating the feasibility of the model transfer learning strategy. This showcases the predictive capability of the Stacking model for novel MMMs based on polymer matrices. However, the Stacking model exhibited poor performance in predicting the TSP(CO_2_/N_2_) of novel MMMs formed with s‐PEO and a‐PEO polymers, with an accuracy of only ≈ 0.48 in the test set. Despite some instances of poor generalization, we believe that using data from known types of polymer materials remains beneficial for model development of new polymers.

Then we select one type of MMM as the “new” material for testing and compared two training strategies (Model prediction, Figure 5c): (a) Only using 10% of the new material data for training (self‐data) and the rest for testing;(b) Using 10% of the data of new material plus all data from other types of polymer MMMs as the training set (model‐data), and the remaining 90% of the new material as the test set. The results are shown in Table S22 (Supporting Information). The difference in prediction results between (a)self‐data and (b)model‐data training strategies, as shown in Figure 5f and Table S22 (Supporting Information), represents the degree of improvement. By comparing the prediction results of the two datasets, we found that the ability of the stacking model to predict new materials was significantly improved using model‐data. This validated our hypothesis and demonstrated the benefit for model generalizability with information from related materials. This provides a better understanding and utilization of multicomponent mixed materials. It also offers valuable guidance and a solid basis for further research and development of MMMs.

In summary, when comparing the extrapolation of formulas to transfer learning, it is evident that transfer learning is more suitable for designing exceptional MMM materials. Formula extrapolation is limited in its ability to extrapolate all features and relies on accurate understanding of data patterns and reasonable assumptions. Based on linear or simple functional forms, its effectiveness is hindered in capturing complex nonlinear relationships. On the other hand, transfer learning predictions can adapt to complex nonlinear relationships by learning the features and patterns of the data. This enables the model to share knowledge and transfer between different but related tasks, reducing the data requirements and workload in model training. We explored the generalization of stacking models across different types of polymers and found that most novel materials exhibited high predictive performance. Even in cases of lower generalization capability, including data from existing polymer materials still contributed to the development of models for new polymers using limited data.

#### 3.5.3 Conclusion

In this work, we have integrated molecular simulation with ML to screen the optimal MMMs for the CO_2_ separation and conducted the transfer evaluation of new materials. The Stacking model incorporating the outstanding RF and XGB algorithms, demonstrated the excellent prediction for the performance of MMMs. Then, the RF model combined with SHAP interpretation was used to uncover the importance of various features, indicating that polymer characteristics and the pore size descriptors (PLD and LCD of MOFs) were the main factors influencing the separation performance. Through the high‐throughput screening, optimal MMMs were identified for the separation of CO_2_ from binary mixtures. Additionally, a design strategy for MMMs was established based on interpretable ML, after the comparison of two extrapolation methods. The multivariate linear regression model exhibited the high predictive performance only for certain features such as PLD and LCD, thus necessitating the use of transfer learning methods to capture and predict the target performance. Given the strong generalization capability demonstrated in the transfer extrapolation tests for different MMMs and gas mixtures, the trained Stacking model was employed to predict and infer CO_2_/N_2_ for new MMMs. The consistent results with experimental calculations demonstrated the validity of this extrapolation method. Finally, an interactive desktop software was developed using the trained Stacking model to facilitate the rapid assessment of CO_2_ separation performance in MMM materials. Drawing on datasets from the field of materials science, this work showcases the remarkable potential of interpretable ML and big data mining in uncovering novel and surprising discoveries.

## Conflict of Interest

The authors declare no conflict of interest.

## Supporting information



Supporting Information

## Data Availability

The data and software supporting the findings of this study can be accessed on Github at https://github.com/haowanae/Pred_MMM_CO2.
