# Supplementary material for: Interpretable Machine‐Learning and Big Data Mining to Predict the CO2 Separation in Polymer‐MOF Mixed Matrix Membranes
Source: Adv Sci (Weinh). 2025 Feb 27;12(16):2405905. doi: 10.1002/advs.202405905 (PMC12021122; doi:10.1002/advs.202405905)
Supplement: Supplementary file 1 — Supporting Information [file ADVS-12-2405905-s001.docx]

**Supplementary Materials**

**Interpretable Machine-Learning and Big Data Mining to Predict the CO_2_ Separation in Polymer-MOF Mixed Matrix Membranes**

*Hao Wan, Yue Fang, Min Hu, Shuya Guo,Zhiqiang Sui**, Xiaoshan Huang, Zili Liu,*

*Yue* Zhao, Hong Liang, Yufang Wu*, Hanyu Gao* , Zhiwei Qiao*

**Table of Contents**

Section S1. Adsorbate Force Field Parameters……………………………………… .S2

Section S2. Characteristics of gas molecules………………………………………….S4

Section S3. The detailed computational methods for MMMs**.**………………………...S5

Section S4. Details of Model Training………………………………………………...S8

Section S5. Univariate analysis of structure-performance relationship equation…….. S16

Section S6. Computational Validation………………………………………………...S19

Section S7. Evaluation of machine learning……………………………………...…... S20

Section S8. Analysis of the relative importance of features………………………...... S27

Section S9. Top-performance MMMs and MOFs……………………………………. S31

Section S10. Relationships between MMM features and properties of gases …….……S37

Section S11. Transfer learning ……………………………………………………….. S40

References……………………………………………………………………… …….. S43

**Section S1. Adsorbate Force Field Parameters**

The atomic structure of MOFs is described by Lennard-Jones (LJ) parameters and electrostatic potentials

$\sum4\varepsilon_{ij}\left[ \left( \frac{\sigma_{ij}}{r_{ij}} \right)^{12}-\left( \frac{\sigma_{ij}}{r_{ij}} \right)^{6} \right]+\sum\frac{q_{i}q_{j}}{4\pi\varepsilon_{0}r_{ij}}$ (1)

where ε_ij_ represents the well depth, s_ij_ represents the collision diameter, q_i_ and q_j_ are the atomic charges carried by atoms i and j, and r_ij_ is the distance between atoms i and j. ε_0_ = 8.8542 × 10^−12^ C^2^ N^−1^ represents the vacuum permittivity. The LJ potential energy parameters for all MOFs were derived from the Universal Force Field (UFF) **^[1]^** and details are shown in Table S1. Numerous simulation studies have demonstrated the accuracy of the UFF in predicting MOF adsorption and diffusion performance **^[2][3]^.**. The atomic charges of MOFs are obtained using the MOF electrostatic-potential-optimized charge scheme (MEPO-Qeq) **^[4]^**.

**Table S1.** Lennard-Jones parameters of atoms in MOFs **^[5]^.**

| **Atom** | ***ε*/*k*_B_ [K]** | ***σ* [Å]** | **Atom** | ***ε*/*k*_B_ [K]** | ***σ* [Å]** | **Atom** | ***ε*/*k*_B_ [K]** | ***σ* [Å]** |
| --- | --- | --- | --- | --- | --- | --- | --- | --- |
| Ac | 16.6 | 3.1 | Ge | 190.69 | 3.81 | Po | 163.52 | 4.2 |
| Ag | 18.11 | 2.8 | Gd | 4.53 | 3 | Pr | 5.03 | 3.21 |
| Al | 254.09 | 4.01 | H | 22.14 | 2.57 | Pt | 40.25 | 2.45 |
| Am | 7.04 | 3.01 | Hf | 36.23 | 2.8 | Pu | 8.05 | 3.05 |
| Ar | 93.08 | 3.45 | Hg | 193.71 | 2.41 | Ra | 203.27 | 3.28 |
| As | 155.47 | 3.77 | Ho | 3.52 | 3.04 | Rb | 20.13 | 3.67 |
| At | 142.89 | 4.23 | I | 170.57 | 4.01 | Re | 33.21 | 2.63 |
| Au | 19.62 | 2.93 | In | 301.39 | 3.98 | Rh | 26.67 | 2.61 |
| B | 90.57 | 3.64 | Ir | 36.73 | 2.53 | Rn | 124.78 | 4.25 |
| Ba | 183.15 | 3.3 | K | 17.61 | 3.4 | Ru | 28.18 | 2.64 |
| Be | 42.77 | 2.45 | Kr | 110.69 | 3.69 | S | 137.86 | 3.59 |
| Bi | 260.63 | 3.89 | La | 8.55 | 3.14 | Sb | 225.91 | 3.94 |
| Bk | 6.54 | 2.97 | Li | 12.58 | 2.18 | Sc | 9.56 | 2.94 |
| Br | 126.29 | 3.73 | Lu | 20.63 | 3.24 | Se | 146.42 | 3.75 |
| C | 52.83 | 3.43 | Lr | 5.53 | 2.88 | Si | 202.27 | 3.83 |
| Ca | 119.75 | 3.03 | Md | 5.53 | 2.92 | Sm | 4.03 | 3.14 |
| Cd | 114.72 | 2.54 | Mg | 55.85 | 2.69 | Sn | 285.28 | 3.91 |
| Ce | 6.54 | 3.17 | Mn | 6.54 | 2.64 | Sr | 118.24 | 3.24 |
| Cf | 6.54 | 2.95 | Mo | 28.18 | 2.72 | Ta | 40.75 | 2.82 |
| Cl | 114.21 | 3.52 | N | 34.72 | 3.26 | Tb | 3.52 | 3.07 |
| Cm | 6.54 | 2.96 | Na | 15.09 | 2.66 | Tc | 24.15 | 2.67 |
| Co | 7.04 | 2.56 | Ne | 21.13 | 2.66 | Te | 200.25 | 3.98 |
| Cr | 7.55 | 2.69 | Nb | 29.69 | 2.82 | Th | 13.08 | 3.03 |
| Cu | 2.52 | 3.11 | Nd | 5.03 | 3.18 | Ti | 8.55 | 2.83 |
| Cs | 22.64 | 4.02 | No | 5.53 | 2.89 | TI | 342.14 | 3.87 |
| Dy | 3.52 | 3.05 | Ni | 7.55 | 2.52 | Tm | 3.02 | 3.01 |
| Eu | 4.03 | 3.11 | Np | 9.56 | 3.05 | U | 11.07 | 3.02 |
| Er | 3.52 | 3.02 | O | 30.19 | 3.12 | V | 8.05 | 2.8 |
| Es | 6.04 | 2.94 | Os | 18.62 | 2.78 | W | 33.71 | 2.73 |
| F | 25.16 | 3 | P | 153.46 | 3. 69 | Xe | 167.04 | 3.92 |
| Fe | 6.54 | 2.59 | Pa | 11.07 | 3.05 | Y | 36.23 | 2.98 |
| Fm | 6.04 | 2.93 | Pb | 333.59 | 3.83 | Yb | 114.72 | 2.99 |
| Fr | 25.16 | 4.37 | Pd | 24.15 | 2.58 | Zn | 62.39 | 2.46 |
| Ga | 208.81 | 3.9 | Pm | 4.53 | 3.16 | Zr | 34.72 | 2.78 |

**Section S2. Characteristics of gas molecules**


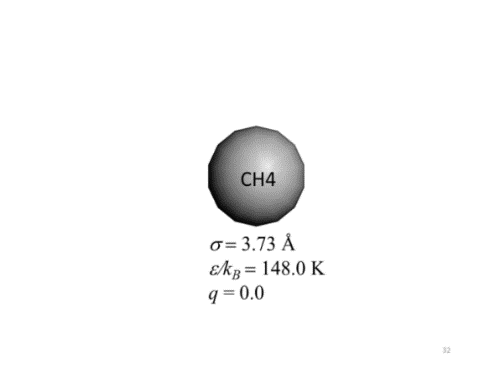


CH_4_


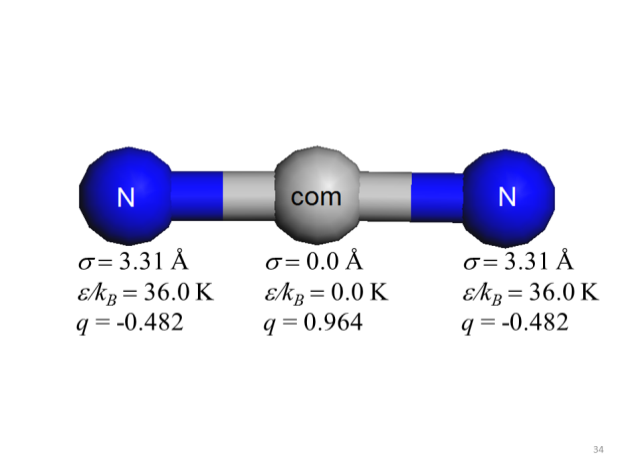


N_2_


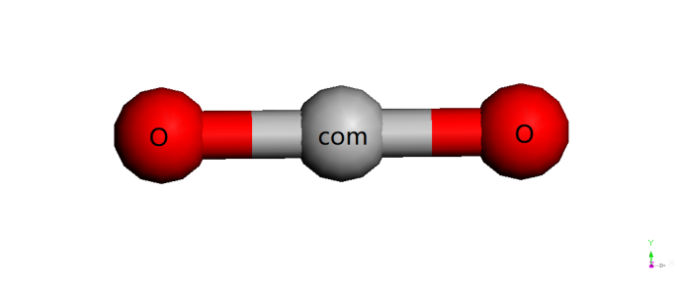


O_2_


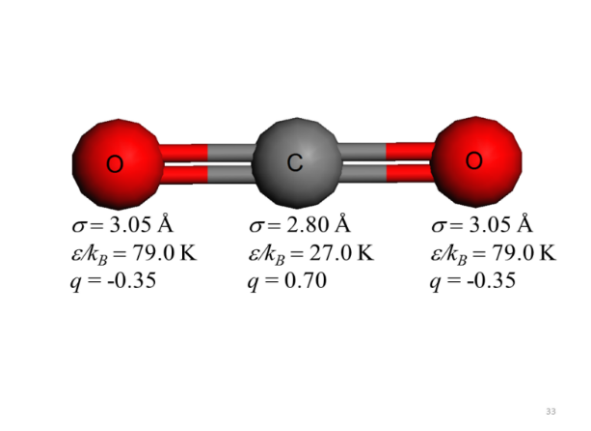


CO_2_


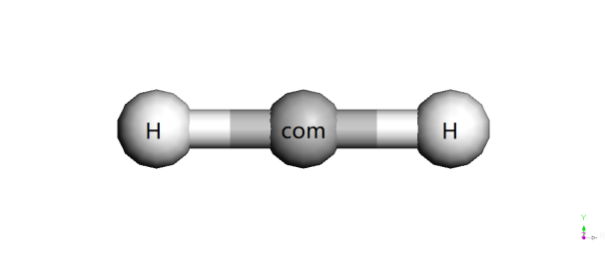


H_2_

**Figure S1.** Models of CH_4_, N_2_, O_2_, H_2_, and CO_2_.

For the 5 gas components (CH_4_, N_2_, O_2_, CO_2_ and H_2_), the force field parameters were obtained from the transferable potentials for phase equilibria (TraPPE) force field**^[6]^**, as listed in Table S2. CH_4_ is a united-atom model, N_2_ is a three-site model with a N-N bond length of 1.10 Å, O_2_ is a three-site molecule, and the C-O bond length for CO_2_ is 1.16 Å with an ∠OCO angle of 180°

**Table S2****.** Lennard–Jones parameters and charges of adsorbates **^[7, 8]^**

| Atom | *ε*/*k*_B_ [K] | *σ* [Å] | Charge (*e*) | Atom | *ε*/*k*_B_ [K] | *σ* [Å] | Charge (*e*) |
| --- | --- | --- | --- | --- | --- | --- | --- |
| C_CO_2_ | 27 | 2.8 | 0.7 | O_O_2_ | 49 | 3.02 | −0.113 |
| O_CO_2_ | 79 | 3.05 | −0.35 | com _O_2_ | 0 | 0 | 0.226 |
| CH_4_ | 148 | 3.73 | 0 | H_H_2_ | 0 | 0 | 0.468 |
| N_N_2_ | 36 | 3.31 | −0.482 | com_H_2_ | 36.7 | 2.96 | -0.936 |
| com_N_2_ | 0 | 0 | 0.964 |  |  |  |  |

**Table S3.** Physical properties of gas molecules.

| Gas | Kinetic diameter | Quadruple moment | Polarizability | Ref |
| --- | --- | --- | --- | --- |
|  | [Å] | [×10^26^/esu cm^2^ ] | [×10^25^/cm^3^ ] |  |
| CH_4_ | 3.76 | 0 | 25.93 | **[9]** |
| N_2_ | 3.64 | 1.52 | 17.403 |  |
| O_2_ | 3.47 | 0.39 | 15.812 |  |
| CO_2_ | 3.3 | 4.3 | 29.11 |  |
| H_2_ | 2.89 | 0.662 | 8.042 |  |
| C_2_H_2_ | 3.3 | 3 | — | **[10]** |

**Section S3.** **The detailed computational methods for MMMs.**

In this work, MMM refers to advanced composite materials composed of polymers as the matrix and MOFs as the embedded inorganic particles. For the MOF part, the Computation-Ready, Experimental MOFs (CoRE-MOFs) database reported by Chung et al. was employed **^[11] [12]^**. And for the polymer component, based on the performance of reported polymers and their combination with MOFs, nine polymers with high, medium, and low permeability were chosen: two polymers with intrinsic microporosity (PIM-1**^[13]^**, PIM-7**^[14]^**), amorphous plasma electrolytic oxidation (a-PEO)**^[15]^**, semi-crystalline plasma electrolytic oxidation (s-PEO), polyphenylene oxide (PPO) **^[16]^**, cellulose acetate (CA) **^[17]^**, and three polyimides (Matrimid, PI-3, and PI-5). The permeability of various gases for nine different polymers are listed in Table S4. We combined these nine polymers and 6013 MOFs in different volume fractions (0.1, 0.2, 0.3) to create 162,351 different MMMs.

In order to predict the permeability of composite materials, Maxwell’s equations were introduced in this study. These equations were originally derived by Maxwell **^[18]^** in 1987 to predict the dielectric properties of composite materials. Later, Bouma et al **^[19]^**. simplified the Maxwell’s equations into a formula, commonly known as the "Maxwell model", under the assumption that the filling particles were spherical. The formula is as follows:

$P_{\mathrm{MMM}}=P_{\mathrm{poly}}\frac{P_{\mathrm{MOF}}\left( 1+2\emptyset_{\mathrm{MOF}} \right)+P_{\mathrm{poly}}（2-2\emptyset_{\mathrm{MOF}}）}{P_{\mathrm{MOF}}\left( 1-\emptyset_{\mathrm{MOF}} \right)+P_{\mathrm{poly}}（2+2\emptyset_{\mathrm{MOF}}）}$ (2)

where *P*_MMM_ is the permeability of the MMM, *P*_poly_ is the permeability of the polymer (matrix), *P*_MOF_ is the permeability of the MOF (filler), and $\emptyset_{\mathrm{MOF}}$ is the volume fraction of the MOF filler particles. Since the Maxwell’s equations assume that the flux pattern around the matrix particles is not affected by other filler particles, the distribution parameters of the fillers on the matrix is not taken into account, and the equations are only applicable to composite materials with low filler content. Despite these simplifying assumptions, this model has been shown to be quite effective in a number of previous works **^[18] [20]^**. Then, three low $\emptyset_{\mathrm{MOF}}$ values (0.1, 0.2, 0.3) were employed to explore the relationship between the $\emptyset_{\mathrm{MOF}}$ and the P_MMM_. Using the predicted permeability, we calculated the ideal selectivity using formula (3). Additionally, to bridge the gap between theoretical materials and actual processes, we assumed that each MMM was a 1 μm thick selective layer and integrated it into the porous carrier, that is, using a part of the separation membrane component as the predicted permeability result for each MMM.

The Henry's law constant of MOF can be used to estimate the gas adsorption performance at low pressures, but it may lead to over-prediction of adsorption at high pressures. In that case, the solution diffusion mechanism **^[21]^** can be used. This mechanism expresses the permeability P_i_ of substance i as the product of its solubility S_i_ and diffusion rate D_i_. The permeation selectivity of substance i to substance j ($S_{\mathrm{perm}{(i}/j)}$) is calculated by the following formula:

$S_{\mathrm{perm}{(i}/j)}=\frac{P_{i}}{P_{j}}=S_{sol{(i}/{j)}}\times S_{diff{(i}/{j)}}$ (3)

where $S_{sol{(i}/{j)}}$ is the solubility selectivity, and the diffusion selectivity (*S_diff_*) can be calculated by

$S_{diff}=D_{i}/D_{j}$ (4)

Through the above formula, the CO_2_/X (X= CH_4_, N_2_, O_2_, H_2_) selectivity for each MOF and MMM can be calculated. In this work, the adsorption selectivity (*S_ads_*) in the adsorption-separation process is also taken into account, and the calculation formula is as follows:

$S_{ads}=N_{i}/N_{j}$(5)

The selectivity and permeability of membranes are the main factors affecting the efficiency of membrane separation technology. However, the common trade-off issue is that the selectivity decreases with the increase of permeability. To address the problem, the TSN weighting method introduced by Shah et al **^[22]^,** was employed to assess the performance of membrane for gas separation. Considering the different orders of magnitude between P and S, we have defined TSP as an evaluation metric.

TSP = (*S*_perm i/j_)·ln *P_i_* (6)

*P*_i_ is the permeability of the separated gas, *S*_perm,_ *_i/j_*, *S*_perm,_ *_i/k_*, …, *S*_perm,_ *_i/n_* are the selectivity of different binary mixtures. In this work, *P_i_* is the CO_2_ permeability of MOFMs, *S*_perm,_*_i/j_*, *S*_perm,_*_i/k_*, …, *S*_perm,_*_i/n_* is the selectivity of five binary mixtures (CO_2_/CH_4_, CO_2_/ H_2_, CO_2_/O_2_, CO_2_/N_2_).

**Table S4.** Permeability performance of original polymer membranes

| Name | *P*H_2_  (barrer) | *P*O_2_ (barrer) | *P*N_2_ (barrer) | *P*CO_2_ (barrer) | *P*CH_4_ (barrer) | *ρ*_poly_ (g/cm^3^) | FFV (cm^3^/g) | Ref. |
| --- | --- | --- | --- | --- | --- | --- | --- | --- |
| PIM-1 | 1300 | 370 | 92 | 2300 | 125 | 1.06 | 0.26 | **[23]**  **[24]** |
| PIM-7 | 860 | 190 | 42 | 1100 | 62 | 1.086 | 0.24 |  |
| a-PEO | 21 | 8.1 | 30 | 143 | 7.1 | 1.209 | 0.139 | **[25]** |
| s-PEO | 1.8 | 0.68 | 0.25 | 12 | 0.6 | 1.124 | 0.188 |  |
| PPO | 113 | 19.1 | 4.65 | 90.68 | 6.43 | 1.057 | 0.181 | **[26]** |
| CA | 2.63 | 0.59 | 0.21 | 6.3 | 0.21 | 1.253 | 0.1614 | **[27]** |
| Matrimid | 22.8 | 1.63 | 0.19 | 8.62 | 0.17 | 1.24 | 0.17 | **[28]** |
| PI-3 | 350 | 67 | 16.5 | 360 | 15 | 1.4423 | 0.132 | **[29]** |
| PI-5 | 210 | 32 | 7.3 | 190 | 5.6 | 1.3982 | 0.137 |  |
| 6FDA-DAM | — | — | 61.3 ± 9.2 | 1245 ± 98 | — | 1.259 | 0.238 | **[30] [31]** |

1 barrer = 3.348 × 10^–16^ mol m (m^2^ s Pa)^-1^ = 10^−10^ cm^3^ (STP) cm (cm^2^·s·cmHg) ^-1^

**^Section S4.^** **^Details of Model Training^**

**Calculation formula of *R^2^*, MAE, RMSE**

Mean Absolute Error (MAE):

$\text{MAE}=\frac{1}{N}\sum_{i=1}^{n} \left| y_{\text{sim}}-y_{\text{pre}} \right|$ (7)

Root Mean Squared Error (RMSE):

$\text{RMSE}=\sqrt{\frac{1}{N}\sum_{i=1}^{n} \left( y_{\text{sim}}-y_{\text{pre}} \right)^{2}}$ (8)

MAE and RMSE are important indicators to measure the quality of machine learning models. MAE is the mean of absolute error, which can evaluate the degree of data change. The smaller the MAE value, the higher the accuracy of the prediction model. RMSE is the arithmetic square root of the mean square error, which can better reflect the actual situation of the predicted value error.

Coefficient of determination(*R*^2^):

$R^{2}=1-\frac{\sum_{i=1}^{n} \left( Y_{i}-Y_{pre} \right)^{2}}{\left( \sum_{i=1}^{n} \left( Y_{i}-\bar{Y} \right)^{2} \right)}$ (9)

In this work, the performance of each ML algorithm was evaluated by calculating the R^2^ values and the RMSE. The various error values for each algorithm were calculated using eq. (8). The R^2^ value was calculated using eq. (9), where n, *Y*_i_, *Y*_pre_, and $\bar{Y}$are the number of MOFs, the simulated *P*CO_2_ and *S*_perm,_ *_i/j_* of gas molecule, the predicted *P*CO_2_ and *S*_perm,_ *_i/j_* of gas molecule, average *P*CO_2_ and *S*_perm,_ *_i/j_* of gas molecule (*P*CO_2_ and *S*_perm_ of ideal binary gas), respectively.

**Overview of machine learning algorithms**

***k* times repeated *k*-fold cross-validation**

*k* times repeated *k*-fold cross-validation**^[32]^** is commonly used to evaluate the predicted performance on different predicted models. All of the data were randomly divided into *k*, where *k* = 5, in which one set was the test set, and the remaining four were training sets. The average of the root means square error (RMSE) and the linear correlation coefficient (*R^2^*) are regarded as an estimated index. This process is repeated five times.


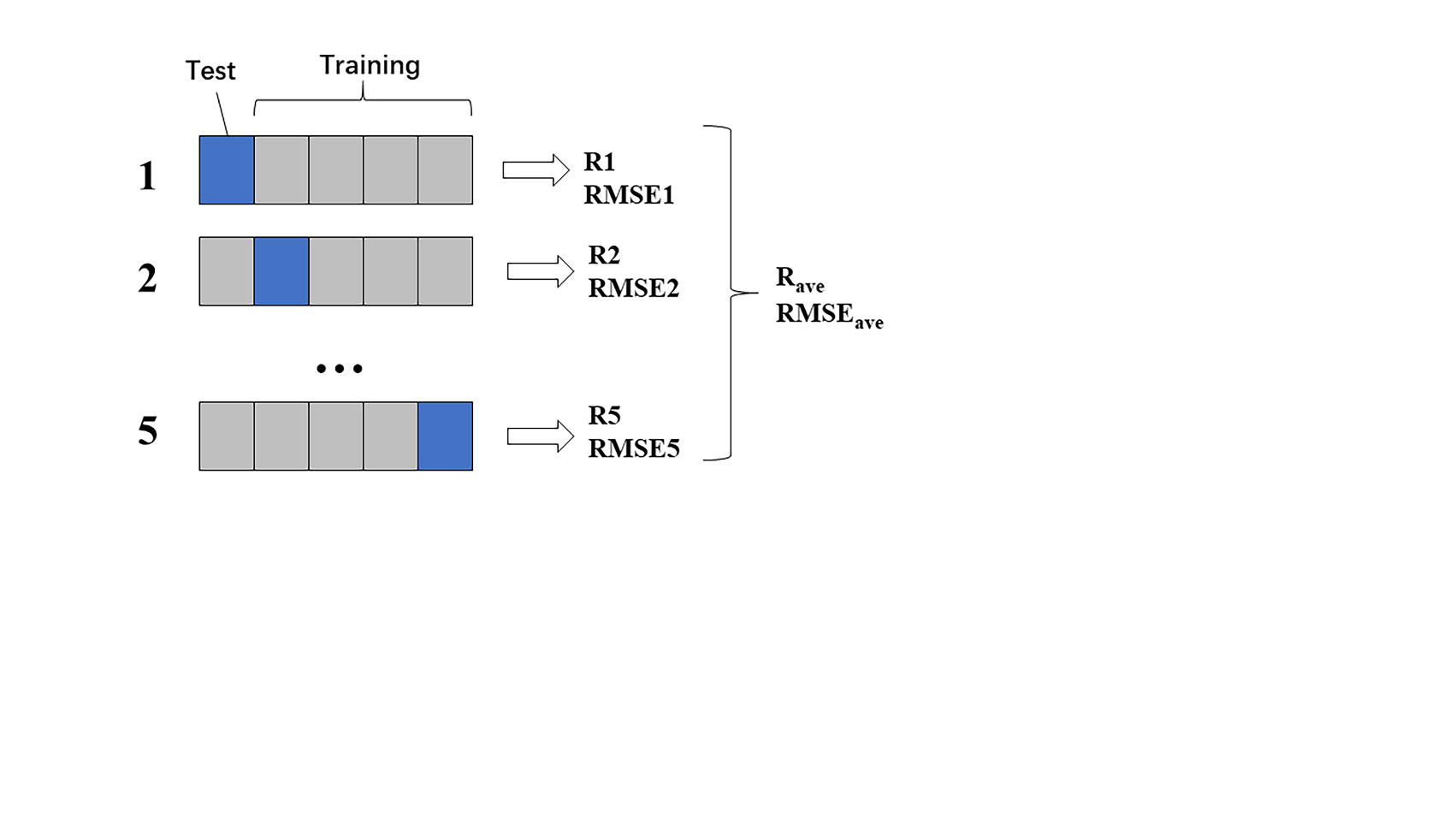


**Figure S2.** k times repeated k-fold cross-validation

**Back-propagation neural network (BPNN)**

BPNN is a type of multilayer feedforward neural network characterized by signal forward and error back-propagation. During signal forward, the input signal is processed step by step from the input layer through the hidden layer until the output layer. Each layer's neuron state affects only the next layer's neuron state. If the output layer does not match the expected result, the network adjusts its weights and benchmarks through back-propagation based on the prediction error, gradually approaching the desired output. **^[33]^** The essence of neural network learning is that the output error passes through reversely from the hidden layer to the input layer, with the error spreading to all units to adjust the weights dynamically. The BPNN topology structure is shown in the following:


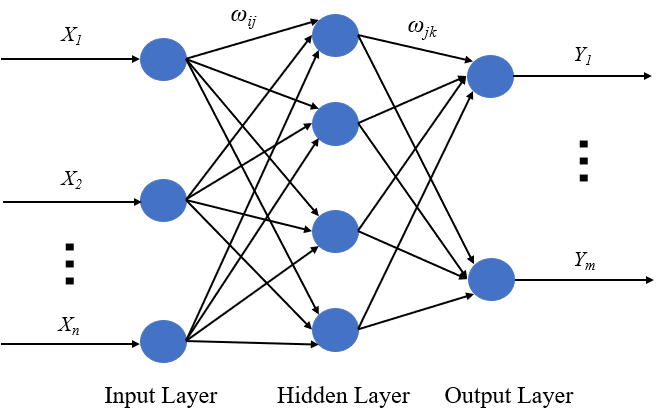


**Figure S3.** BPNN Algorithm Schematic

In the figure, *X_1_*, *X_2_*, …, *X_n_* are the input values, *Y_1_*, *Y_2_*, ..., and *Y_m_* are the predictive values in the BPNN, and *ω_ij_* and *ω_jk_* are the weights in the BPNN. The BPNN is a nonlinear function; the network input values and predicted values are the function of the independent variable and dependent variable, respectively. When inputting a node number as *n*, the output node number is *m*, and a functional mapping relationship is expressed from the dependent variables of *n* to the independent variables of *m* by the BPNN. When we predict different data by the ways of BPNN, first, the data are trained with associative memory and the ability to predict by the network. When the network output error was reduced to an acceptable level or the pre-set amount of learning, it is terminated. Finally, the trained network is used to classify new data, fitting, and predicting. BPNN is a model based on neural networks. In this case, it had 5 hidden layers, with each hidden layer containing 190 nodes. The maximum number of iterations was set to 200. To prevent overfitting, the penalty coefficient for L2 regularization was set as 0.0001.

**Random Forest (RF)**

**
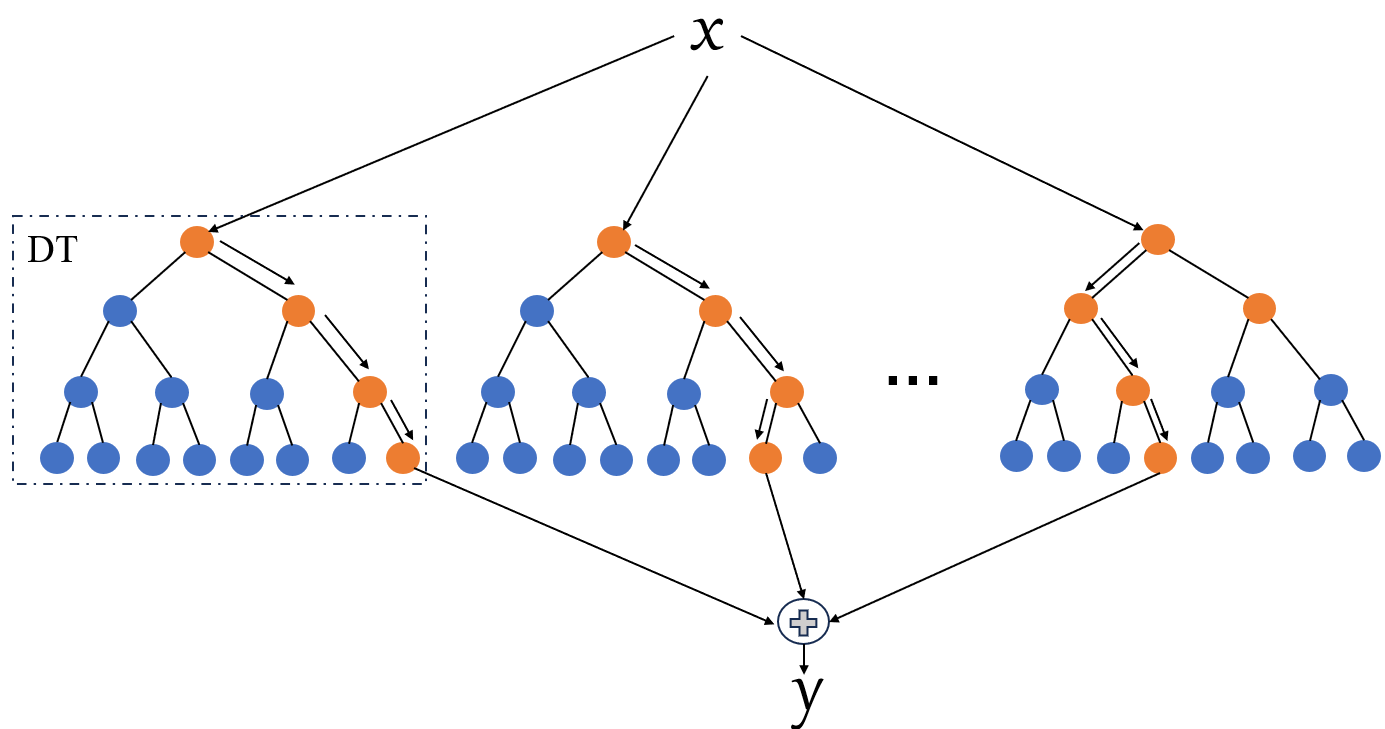
**

**Figure S4.** RF Algorithm Schematic

The general structure of RF and BAT is very similar. RF is also used to conduct *n* random sampling, plant trees and obtain the average. However, RF modified the part of Greedy Search in the DT: the BAT will consider all of the features for each node for split and then select the optimal features as a node; To increase the randomness of RF, only *k* features are used to make the selection for each node for a split, thus reducing the relevance between trees. **^[34]^** In RF, a random split of the dataset is used to construct the decision trees. The number of trees was 125, and the maximum depth of the trees was 25. The criterion used was set as “squared error”.

**Extra Trees**

The general structure of RF and ET is very similar. Extra Trees algorithm (also known as Extremely Randomized Trees) is an ensemble method based on decision trees that uses randomized strategies to construct multiple decision tree models and combines them to achieve better predictive performance. When constructing each decision tree, Extra Trees algorithm employs two randomization strategies: randomly selecting a subset of features and using these features to construct the decision rules for each node, and randomly selecting the splitting point for each node. These randomization strategies allow Extra Trees algorithm to more thoroughly explore the feature space, reduce model variance, and improve model generalization ability. During prediction using Extra Trees algorithm, each decision tree predicts the sample and the results are then aggregated using either voting or averaging. This aggregation further reduces model variance and improves predictive accuracy. In summary, Extra Trees algorithm is an ensemble method based on decision trees that uses randomized strategies to construct multiple decision tree models and combines them to achieve better predictive performance. It performs well in solving both classification and regression problems. ET is an ensemble model built based on decision trees and introduce more randomness during the construction of decision trees.


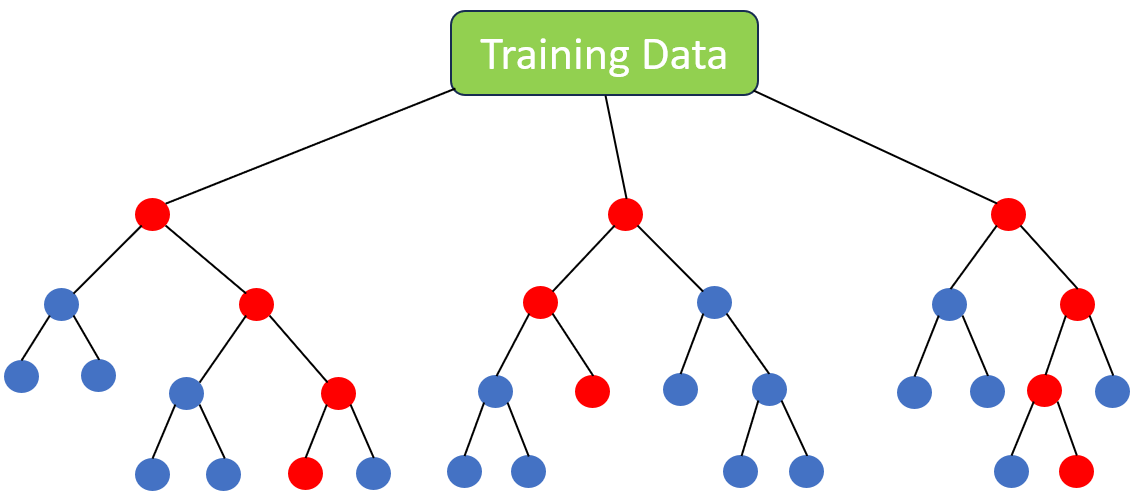


**Figure S5.** ET Algorithm Schematic

**Extreme gradient boosting**

Extreme gradient boosting (XGB)**^[35]^** is a machine learning system designed for speed and efficiency. It is essentially a gradient boosting decision tree algorithm that uses an ensemble approach to improve performance. The XGB algorithm consists of three main parts: the ensemble algorithm, the weak learner, and other application-specific processes. During training, XGB adds new trees to the ensemble while applying gradient descent to minimize the loss. This process involves learning a new function to fit the residuals predicted in the previous iteration until a satisfactory effect is achieved. Once k trees have been trained, the algorithm predicts scores for new samples by mapping each sample to a corresponding leaf node and computing a score for each leaf node. The final prediction score is the sum of scores predicted by each tree.

In addition to the standard operations of the gradient boosting tree algorithm, XGB incorporates regular terms into the objective function based on the original loss function. It also uses the second-order Taylor approximation to optimize the objective function, resulting in significant improvements in performance. XGB is developed based on the GBDT. In XGB, the maximum number of trees was set to 100, with a maximum tree depth of 12 and a learning rate of 0.1. To prevent overfitting, the weight for L1 regularization was 0.8, while the L2 regularization term was 1.0.

^
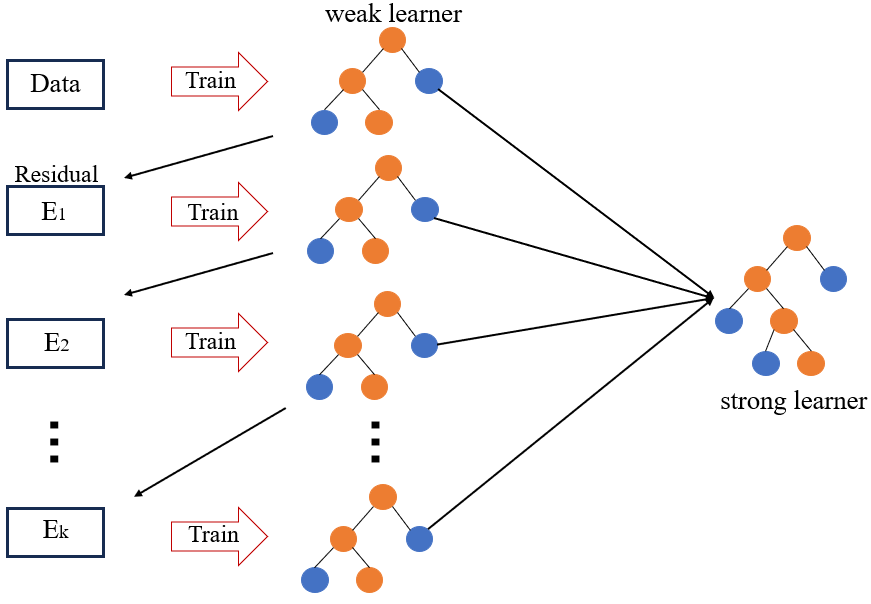
^

**Figure S6.** XGB Algorithm Schematic

**Gradient boosting decision tree**

Gradient boosting decision tree (GBDT) **^[36]^** is an ensemble method that aims to provide a more accurate estimate of the response variable by consecutively fitting new models. The GBDT algorithm consists of three key elements: the loss function to be optimized, the weak learner to make predictions, and the additive model to add weak learners to minimize the loss function.

In GBDT, the decision tree is used as the basic learner, and the loss function chosen is the 'deviance' loss. The learning process involves fitting a sequence of decision trees, with each new tree being trained to fit the conclusions and residuals of all previous trees. The output of the current decision tree is added to the ensemble of previous trees to minimize the loss function. The procedure results in a continuous error fitting process that improves the accuracy of the response variable estimate.

Overall, GBDT is an effective machine learning algorithm that combines decision trees and gradient boosting to achieve high accuracy in predicting the response variable. The GBDT algorithm consists of multiple decision trees and involves training an initial decision tree model using a training dataset. The predictive performance is improved through iterative training. In GBDT, the number of trees was 100, the maximum depth of the decision trees was 5, and the learning rate was 0.1.

^
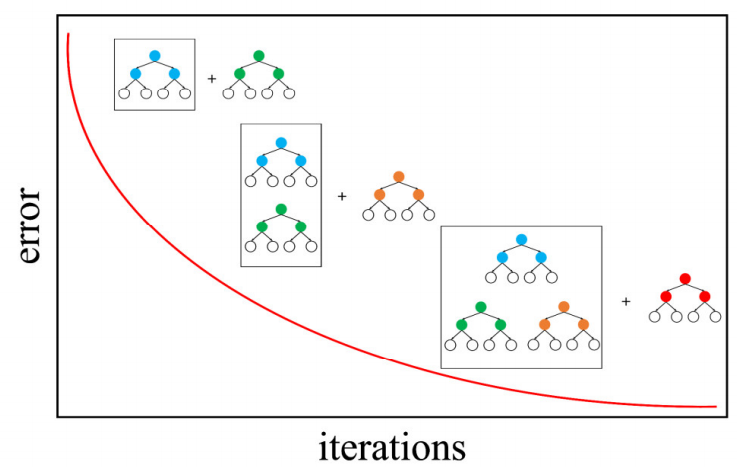
^

**Figure S7.** GBDT Algorithm Schematic

**K-Nearest Neighbor**

k-nearest neighbors (KNN) **^[37]^** is a simple algorithm classifying objects based on a metric distance between testing features and each feature in the feature space. The classification results of objects samples belong to the classification of most of the k training set samples that are closest to the object sample. KNN is a fundamental non-parametric regression algorithm that makes predictions based on the distances between samples.

^
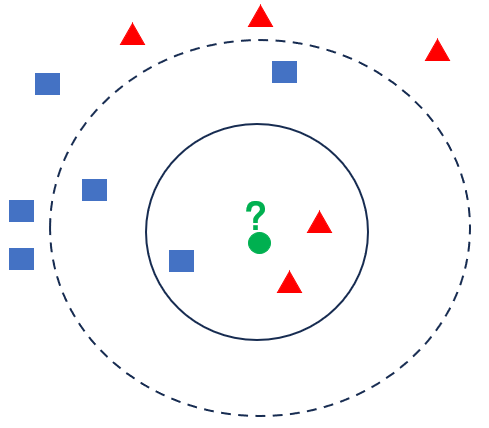
^

**Figure S8.** KNN Algorithm Schematic

**Stacking Ensemble Regression**

Stacking Ensemble Regression**^[38]^** i is a model fusion method that improves model accuracy and robustness by using the output of multiple basic regressors as a new training set for a meta-regressor. In Stacking Ensemble Regression, multiple different basic regressors are first used to predict the same set of training data, resulting in a new set of predictions. These predictions are then used as new features and input to the meta-regressor, which is typically a simple linear model such as ridge regression or Lasso regression. Stacking Ensemble Regression combines the output of multiple basic regressors and optimizes them with the meta-regressor to improve prediction accuracy and robustness. Since different basic regressors may have different biases and variances, Stacking Ensemble Regression reduces model variance by combining the predictions of different models, thereby improving model robustness and generalization ability.


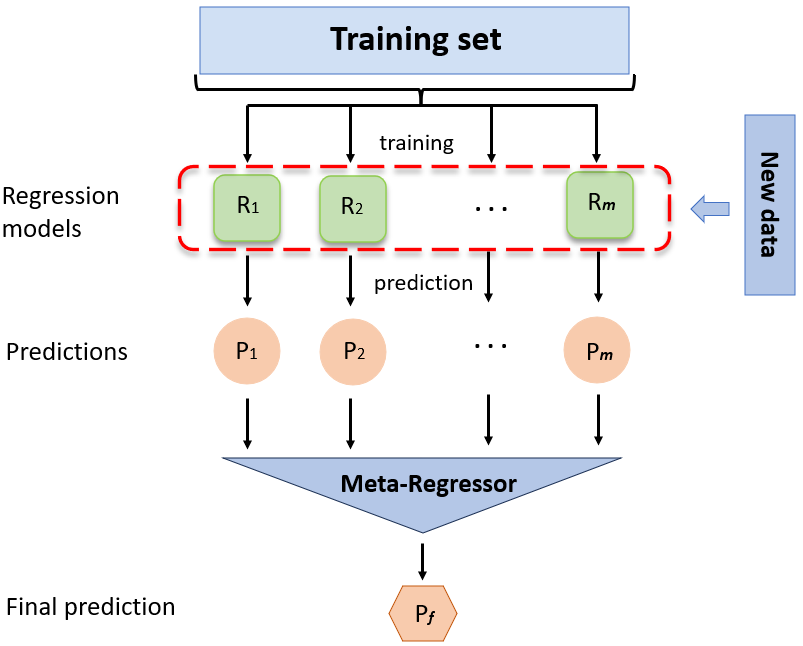


**Figure S9.** Stacking Ensemble Regression Algorithm Schematic

In summary, Stacking Ensemble Regression is an effective model fusion method that can improve prediction accuracy and robustness in regression problems. In this work, the performance of the RF and XGB regression algorithms was selected for the base regression. The predictions from these two regression algorithms were further combined using the Stacking technique, where the fusion algorithm logic was based on XGB. The Stacking model was ultimately employed to predict the CO_2_ separation performance. The original code for stacking can be found at https://scikit-learn.org/stable/modules/generated/sklearn.ensemble.StackingRegressor.html. And the modified code utilized by the authors, along with the detailed parameter settings, can be accessed in the "Code" folder on GitHub at https://github.com/haowanae/Pred_MMM_CO2/tree/master.

Table S5 outlines the training hyperparameters for different machine learning models, which are crucial for optimizing their performance on the specified datasets. Below is a brief overview of the training details for each algorithm:Random Forest (RF): The model is configured with 125 estimators and a maximum depth of 25, striking a balance between complexity and generalization. The random state is set to 90 to ensure reproducibility, and the criterion for splitting is 'squared_error' to minimize prediction error.Gradient Boosting Decision Tree (GBDT): The GBDT model employs a learning rate of 0.1 for gradual improvements in predictions. It uses 400 estimators for one dataset and 500 for another, with maximum depths of 10 and 12, respectively. The loss function is set to 'squared_error', and a subsample rate of 1 is applied. The alpha parameter is fixed at 0.5, ensuring robust regularization. XGBoost: In this model, 85 and 120 estimators are used for the respective datasets, with maximum depths of 12 and 14. The minimum child weight is approximately set to 2, and a subsample ratio of 0.8 is applied to enhance robustness. The regularization parameters (reg_alpha and reg_lambda) are both set to 0.8 and 1, respectively, while the learning rate is finely tuned at 0.2823.Extra Trees (ET): The Extra Trees model consists of 150 estimators, with a maximum feature setting of 0.99 to increase tree diversity. The minimum samples per leaf is set to 3, and bootstrap sampling is disabled to ensure that each tree is built using the entire dataset. The minimum samples split parameter is set to 2. Backpropagation Neural Network (BPNN): This model has a hidden layer size of 190 and uses the ReLU activation function to introduce non-linearity. The Adam optimizer is selected for efficient training, with an alpha value of 0.001 and a maximum iteration count of 200 to control overfitting. K-Nearest Neighbors (KNN): The KNN algorithm is configured with 8 neighbors and a leaf size of 40 to balance memory usage and computational speed.

**Table S5.** Hyperparameters set in machine learning methods.

| Model | Hyperparameter | Value (On *P*) | Value (On CO2/X） |
| --- | --- | --- | --- |
| RF | n_estimators | 125 | 125 |
|  | max_depth | 25 | 25 |
|  | random_state | 90 | 90 |
|  | criterion | 'squared_error' | 'squared_error' |
| GBDT | learning_rate | 0.1 | 0.1 |
|  | loss | 'squared_error' | 'squared_error' |
|  | n_estimators | 400 | 500 |
|  | subsample | 1 | 1 |
|  | max_depth | 10 | 12 |
|  | alpha | 0.5 | 0.5 |
|  | verbose | 0 | 0 |
|  | max_leaf_nodes | None | None |
|  | warm_start | False | FALSE |
| XGBoost | n_estimators | 85 | 120 |
|  | max_depth | 12 | 14 |
|  | min_child_weight | 2.136 | 2 |
|  | subsample=0.8 | 0.8 | 0.8 |
|  | gamma | 0.336 | 0 |
|  | colsample_bytree | 0.8 | 0.8 |
|  | nthread | None | None |
|  | reg_alpha | 0.8 | 0.8 |
|  | reg_lambda | 1 | 1 |
|  | learning_rate | 0.2823 | 0.2823 |
|  | n_jobs | -1 | -1 |
| ET | n_estimators | 150 | 150 |
|  | max_features | 0.99 | 0.99 |
|  | min_samples_leaf | 3 | 3 |
|  | bootstrap | False | FALSE |
|  | min_samples_split | 2 | 2 |
| BPNN | hidden_layer_sizes | 190 | 190 |
|  | activation | relu | relu |
|  | solver | adam | adam |
|  | alpha | 0.001 | 0.001 |
|  | max_iter | 200 | 200 |
| KNN | n_neighbors | 8 | 8 |
|  | leaf_size | 40 | 40 |

**Section S5.** **Univariate analysis of structure-performance relationship.**

First, the relationship between the features of MOFs and their gas performance was analyzed. Subsequently, the direct relationship between the CO_2_ separation performance of MOFs and their features was investigated.

**Pearson correlation coefficient:**

The Pearson product-moment correlation coefficient (PPMCC or PCCs), which varies in value from -1 to 1, is used to calculate the correlation between the two variables, X and Y. A Pearson product-moment correlation coefficient between two variables is defined as the quotient of covariance and standard deviation between two variables:

$r=\frac{\sum_{i=1}^{n} (X_{i}-\bar{X})(Y_{i}-\bar{Y})}{\sqrt{\sum_{i=1}^{n} {(Y_{i}-\bar{Y})}^{2}}}$ (10)


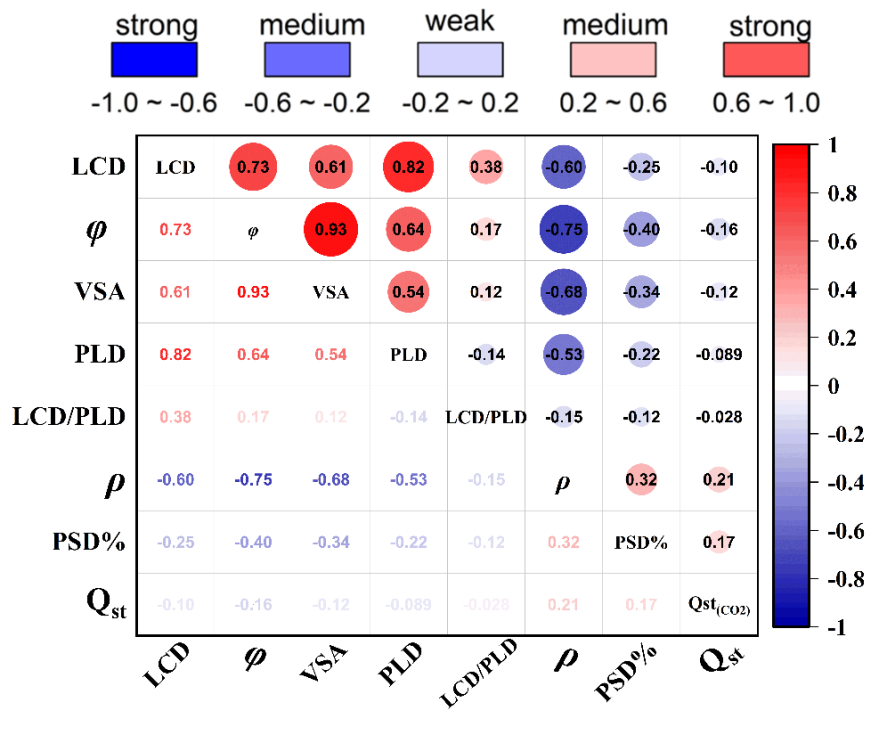


**Figure S10.** Pearson correlation coefficient heatmap of MOF feature descriptors.

**
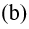

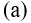
**^
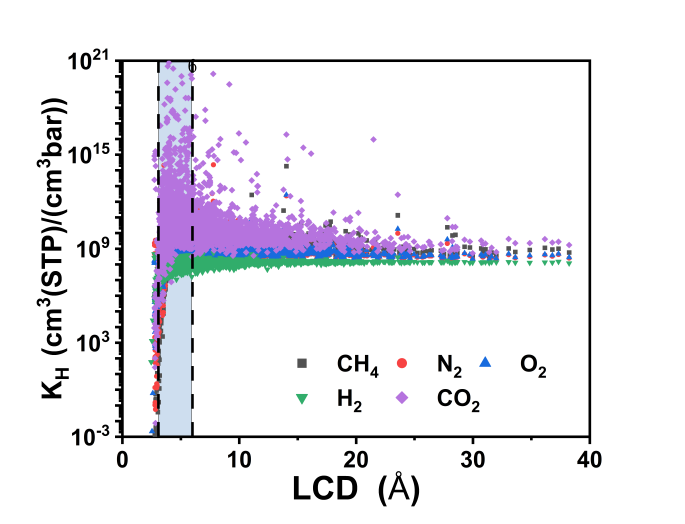

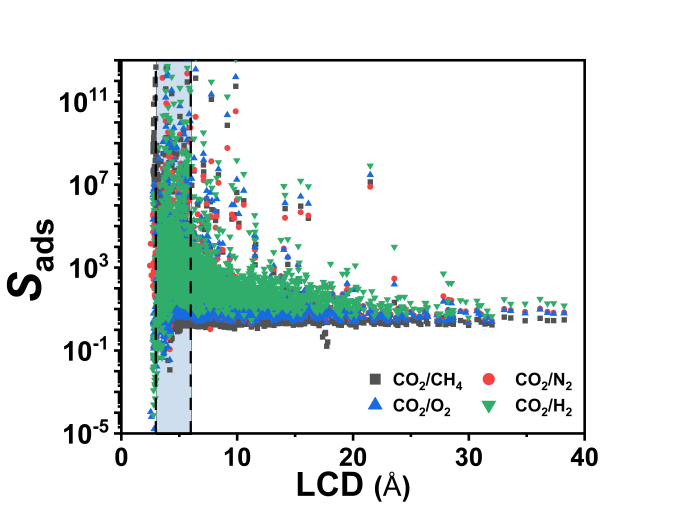
^

**Figure S11.** The relationship between (a) the Henry coefficient of CH_4_, N_2_, O_2_, H_2_, CO_2_ and the LCD, and (b) the *S_ads_* and the LCD relationship.

**
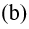

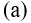

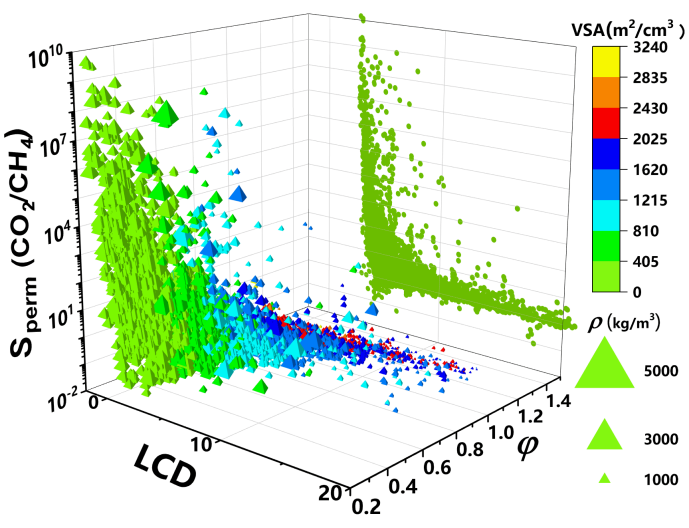

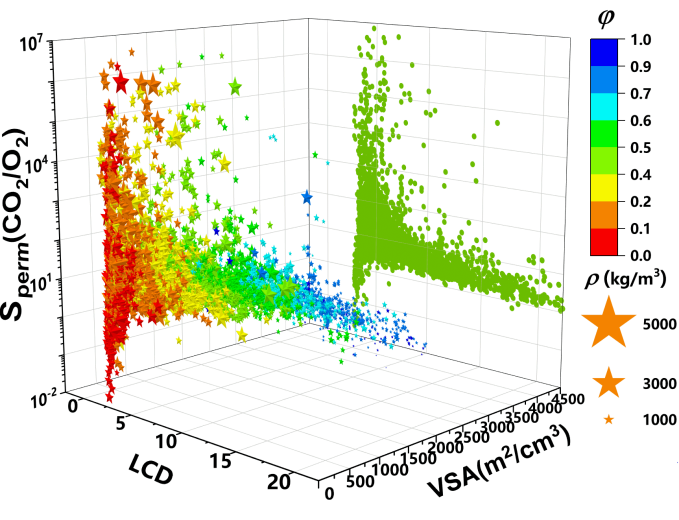
**

**
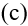

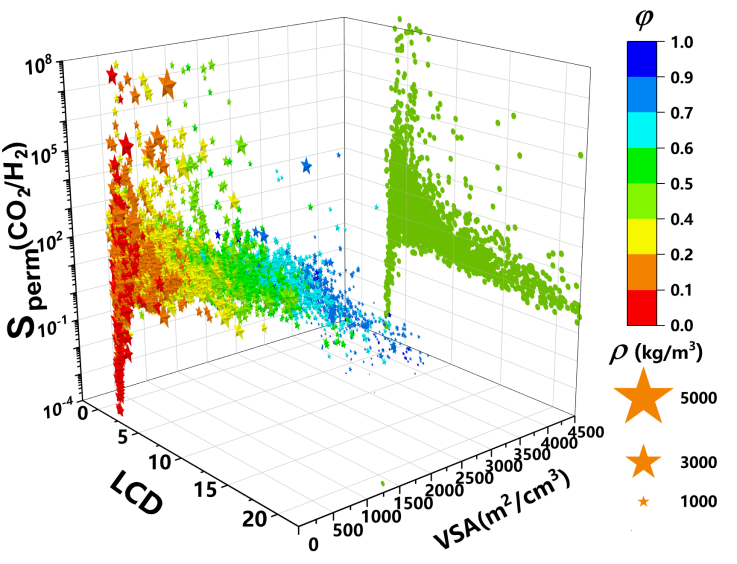
**

**Figure S12.** The relationship between (a) *S*_perm_ (CO_2_/CH_4_), (b) *S*_perm_ (CO_2_/O_2_), (c) *S*_perm_ (CO_2_/H_2_) and PLD, VSA, *ρ*, and *φ*. The size of the symbol represents *ρ*, and the color of the symbol represents VSA or *φ*.

**Section S6. Computational Validation**


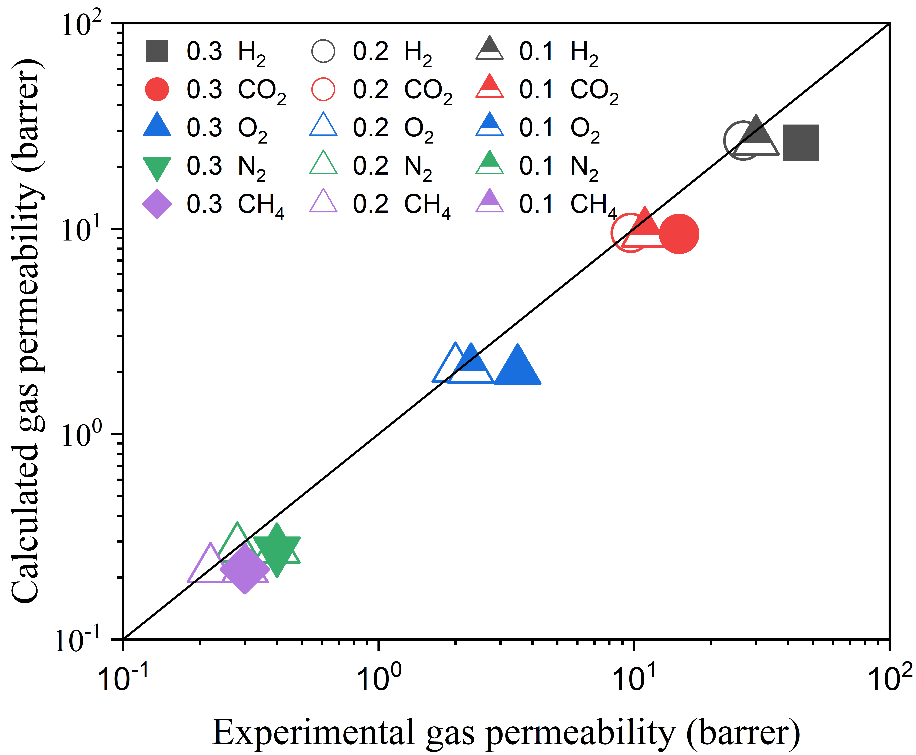


**Figure S13.** Comparison between the gas permeation of MMM predicted using Maxwell equation and the experimentally measured gas permeation.

Figure S13 presents a comparative plot of the theoretical permeation values calculated for MOF-5 (at different volume fractions: 0.1, 0.2, 0.3) with the polymer Matrimid® for five gases (H_2_, CO_2_, O_2_, N_2_, CH_4_) against the actual permeation values obtained from literature sources. It can be observed that the calculated permeation values of the MMM membrane align well with the experimental values, indicating the relative accuracy of the Maxwell model.

The calculation method in this work has been used and compared with experiments in a series of previous studies,^[39-46]^ which has been demonstrated as accuracy. The simulated and experimental permeability are compared, as shown in following Figure S14a.^[47-51]^ Besides, the *S_perm_* in our work and previous studies (simulations) are also compared, as shown in following Figure S14b^.[40, 52-54]^


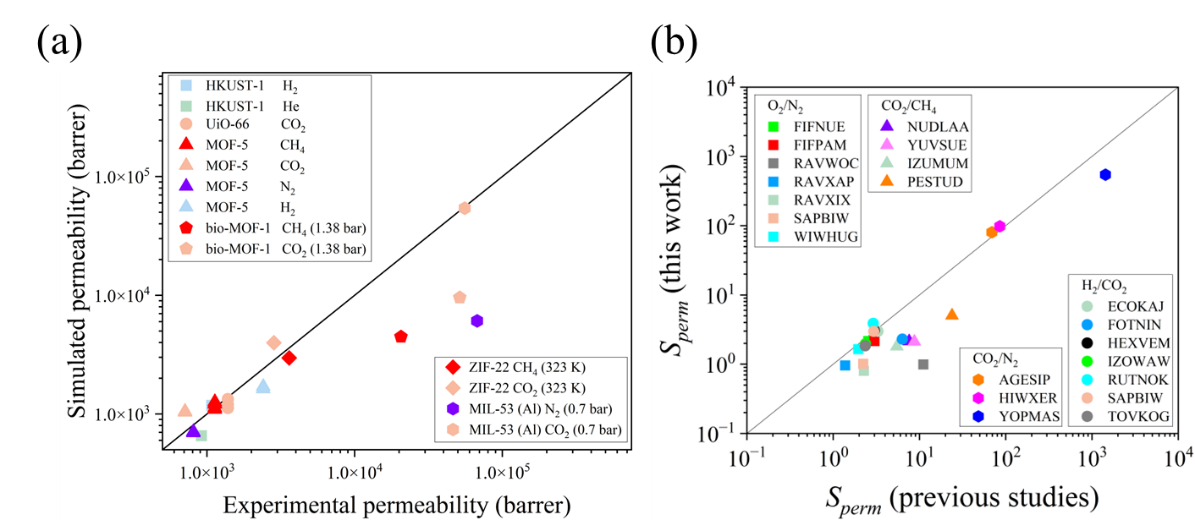


**Figure S14.** Comparison of the permeance performance of MOF membranes in our work and those in previous studies.

**Section S7.** **Evaluation of machine learning**

**Table S6.** The prediction and testing set results of six ML algorithms for MMM performance

| Performance indicator | ML Performance | RF | XGB | ET | GBDT | KNN | BPNN | Stacking |
| --- | --- | --- | --- | --- | --- | --- | --- | --- |
| *P_CO2_* | *R^2^* | 0.966 | 0.968 | 0.964 | 0.952 | 0.948 | 0.962 | 0.980 |
|  | MAE | 57.740 | 54.460 | 59.070 | 81.540 | 73.330 | 66.110 | 43.800 |
|  | RMSE | 156.530 | 151.950 | 160.870 | 184.210 | 195.310 | 166.930 | 115.900 |
| *S*_perm_ (CO_2_/N_2_) | *R^2^* | 0.971 | 0.975 | 0.964 | 0.949 | 0.909 | 0.959 | 0.984 |
|  | MAE | 1.054 | 0.945 | 1.141 | 1.610 | 1.981 | 1.334 | 0.860 |
|  | RMSE | 2.319 | 2.258 | 2.660 | 3.152 | 4.293 | 2.893 | 1.750 |
| TSP(CO_2_/N_2_) | *R^2^* | 0.935 | 0.945 | 0.917 | 0.873 | 0.850 | 0.905 | 0.968 |
|  | MAE | 6.449 | 5.978 | 7.119 | 10.370 | 10.685 | 8.499 | 5.060 |
|  | RMSE | 14.622 | 13.827 | 16.418 | 20.370 | 23.115 | 18.150 | 10.260 |
| TSP(CO_2_/O_2_) | *R^2^* | 0.928 | 0.948 | 0.925 | 0.901 | 0.867 | 0.933 | 0.968 |
|  | MAE | 2.678 | 2.247 | 2.781 | 3.833 | 4.342 | 2.848 | 2.100 |
|  | RMSE | 6.450 | 5.494 | 6.530 | 7.512 | 8.848 | 6.289 | 4.200 |
| TSP(CO_2_/H_2_) | *R^2^* | 0.949 | 0.957 | 0.955 | 0.929 | 0.895 | 0.942 | 0.976 |
|  | MAE | 1.129 | 1.016 | 1.111 | 1.623 | 1.915 | 0.942 | 0.910 |
|  | RMSE | 2.652 | 2.477 | 2.492 | 3.125 | 3.864 | 2.864 | 1.790 |
| TSP(CO_2_/CH_4_) | *R^2^* | 0.862 | 0.900 | 0.847 | 0.792 | 0.760 | 0.876 | 0.940 |
|  | MAE | 8.289 | 7.034 | 8.989 | 12.520 | 11.920 | 8.784 | 6.470 |
|  | RMSE | 19.252 | 16.510 | 20.390 | 23.780 | 25.765 | 18.610 | 12.750 |


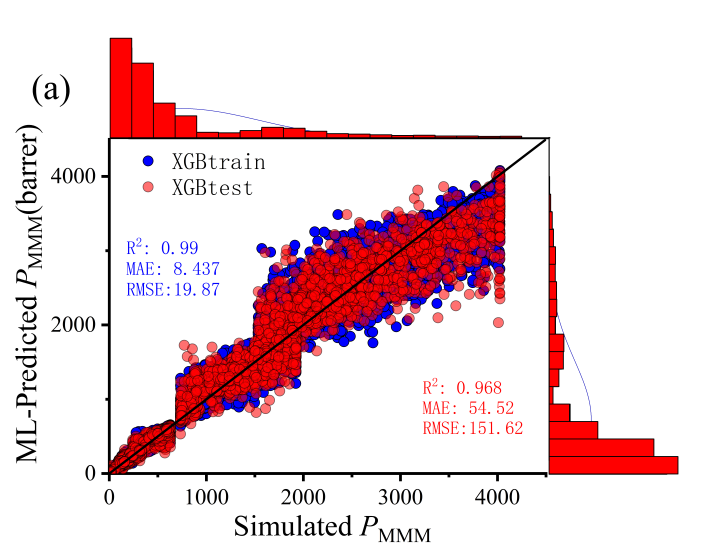
^
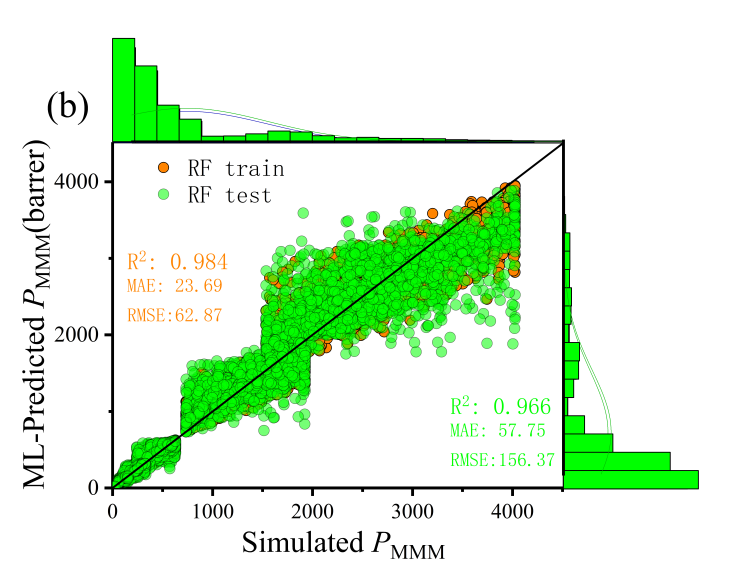
^

^
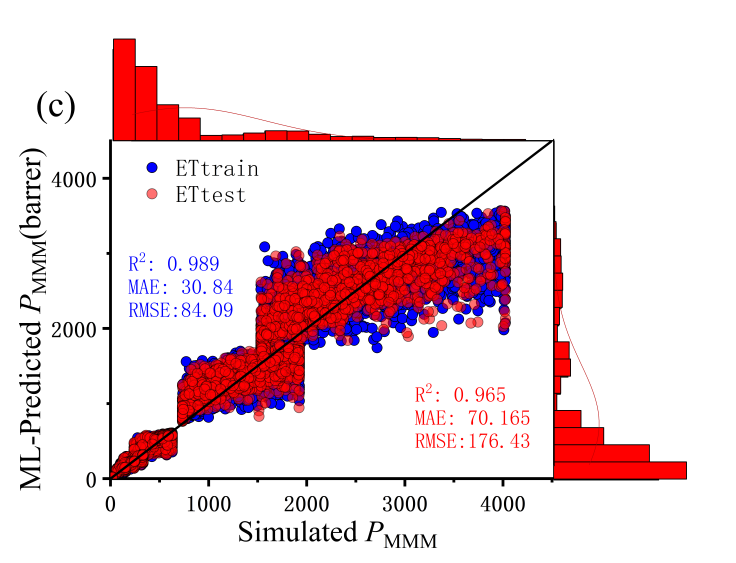

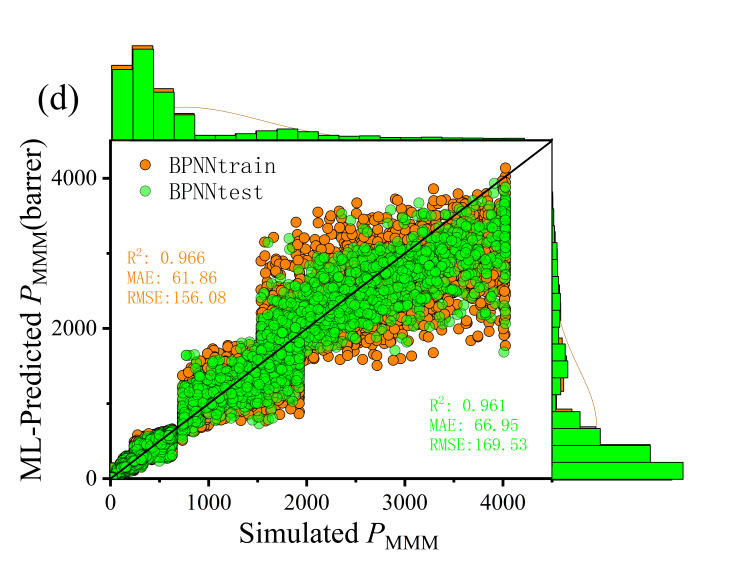

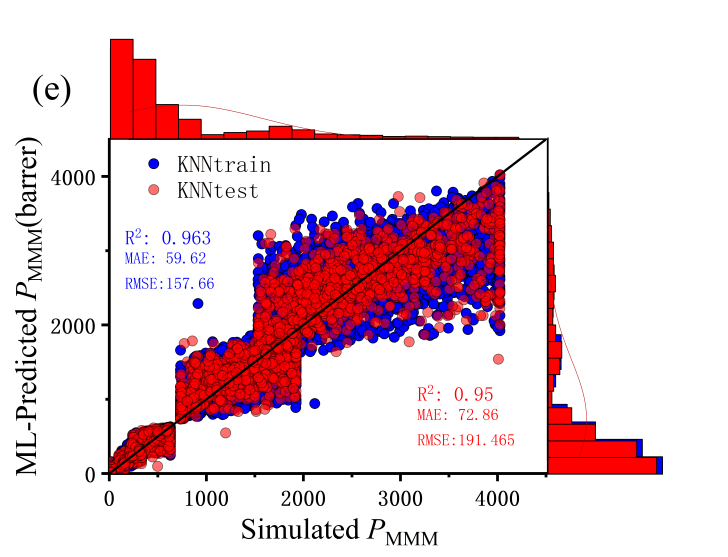

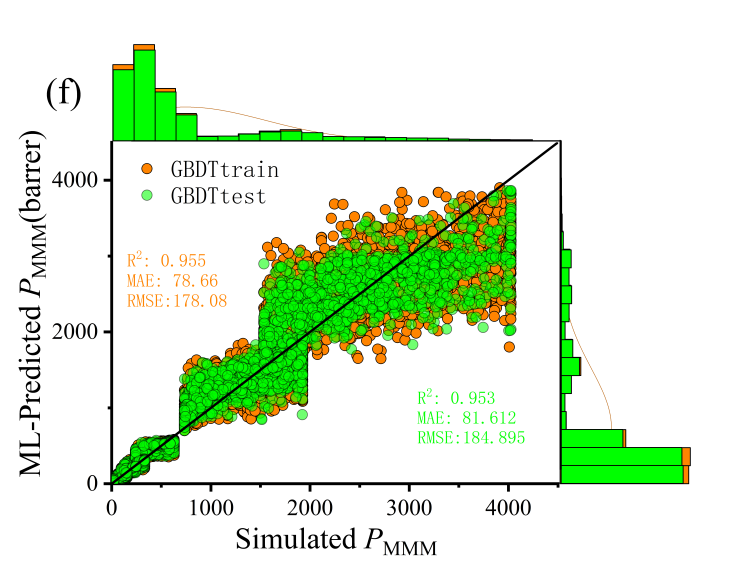
^

**Figure S15.** The prediction results of six ML algorithms for MMM's ***P*CO_2_**. (a) XGB, (b) RF, (c) ET, (d) BPNN, (e) GBDT, and (f) KNN. The color of the dots represents different dataset. Blue (orange) symbols represent training data, and red (green) symbols represent testing data.


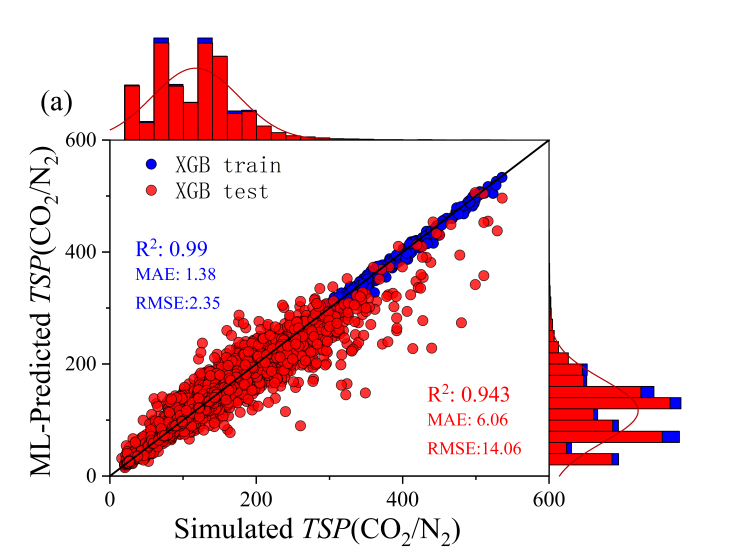

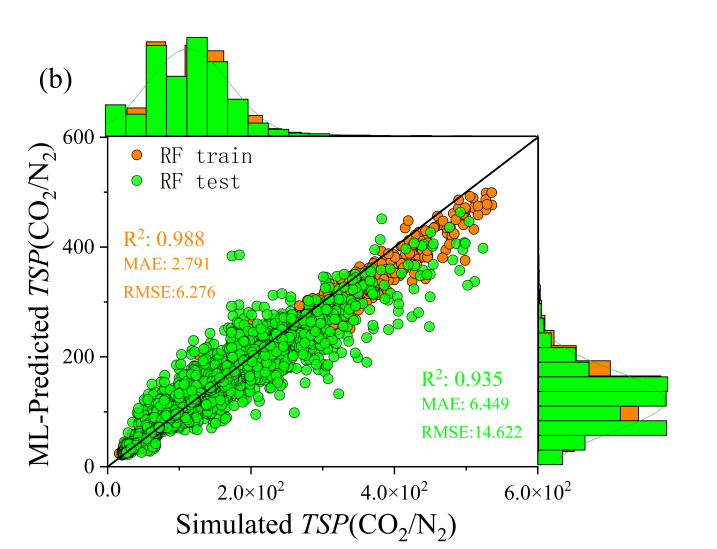

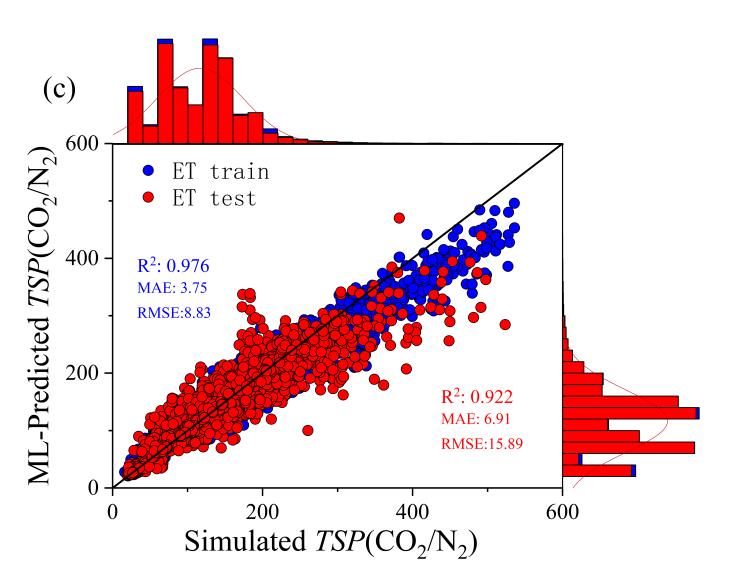

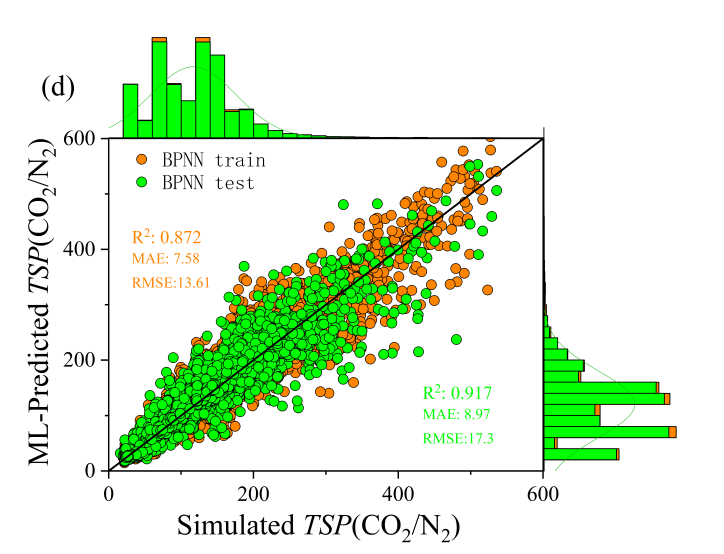

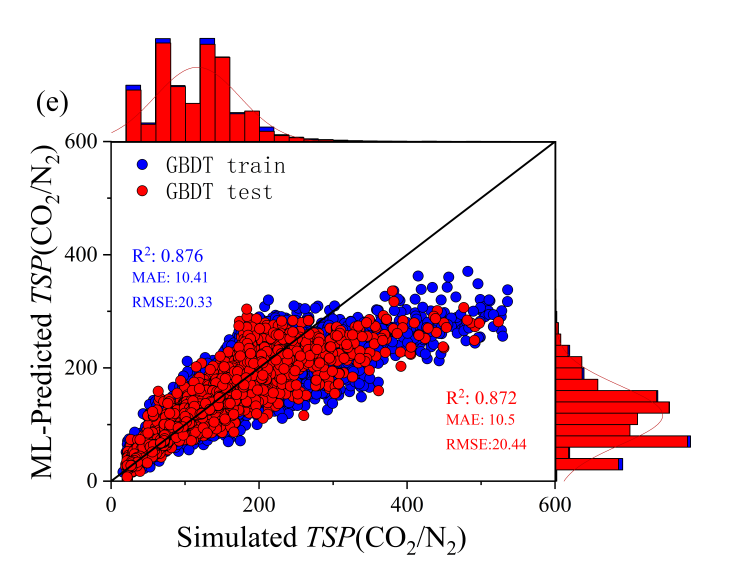

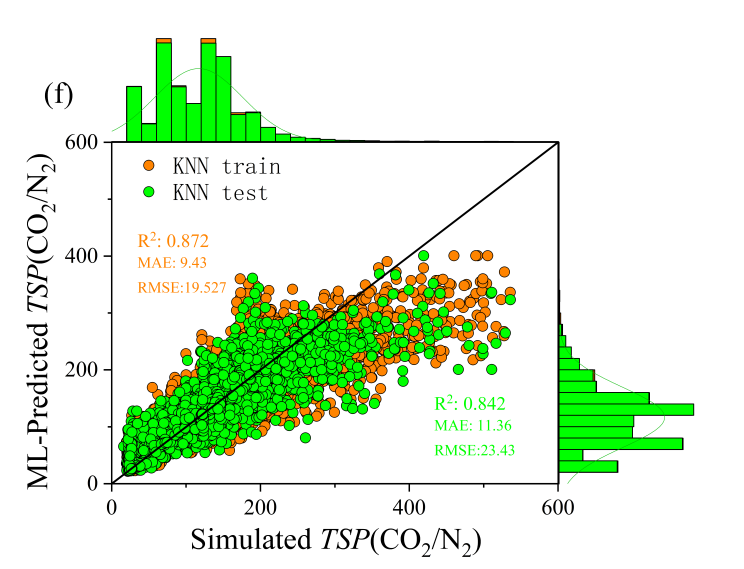


**Figure S16.** The prediction results of six ML algorithms for MMM's ***TSP*(CO_2_/N_2_)**. (a) XGB, (b) RF, (c) ET, (d) BPNN, (e) GBDT, and (f) KNN. The color of the dots represents different dataset. Blue (orange) symbols represent training data, and red (green) symbols represent testing data.

**
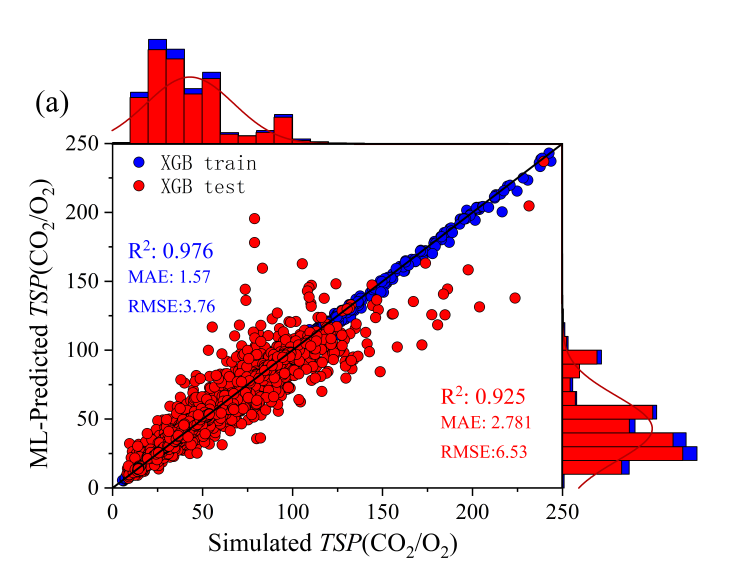

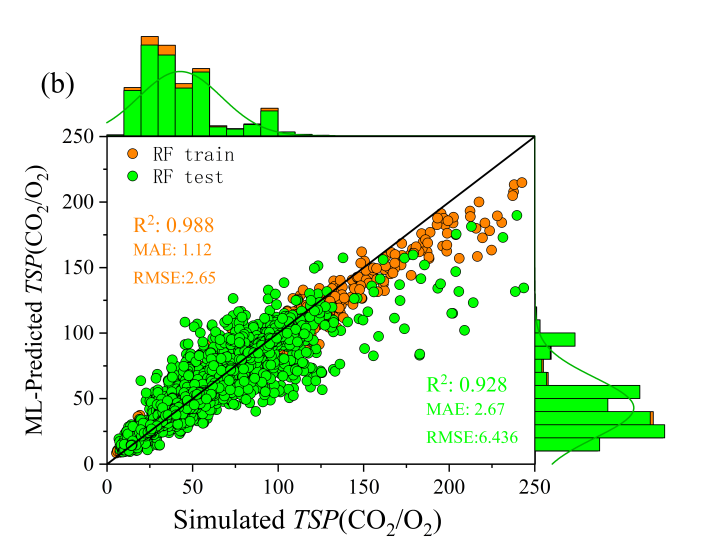
**

**
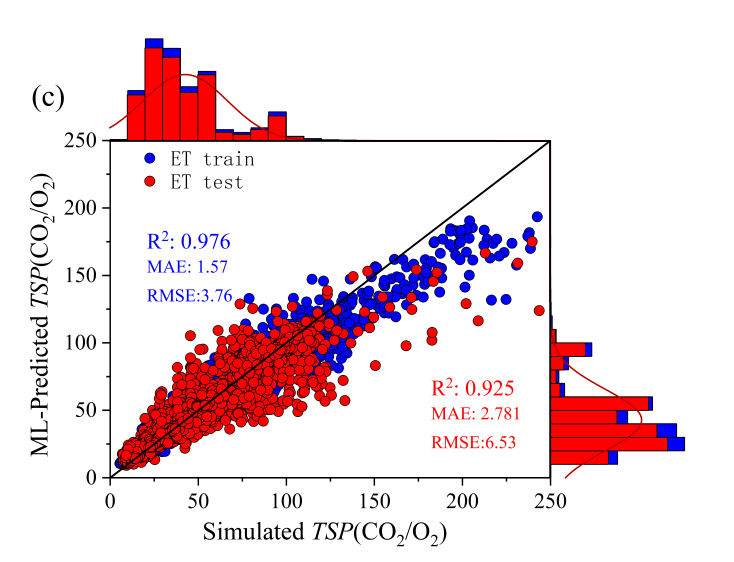

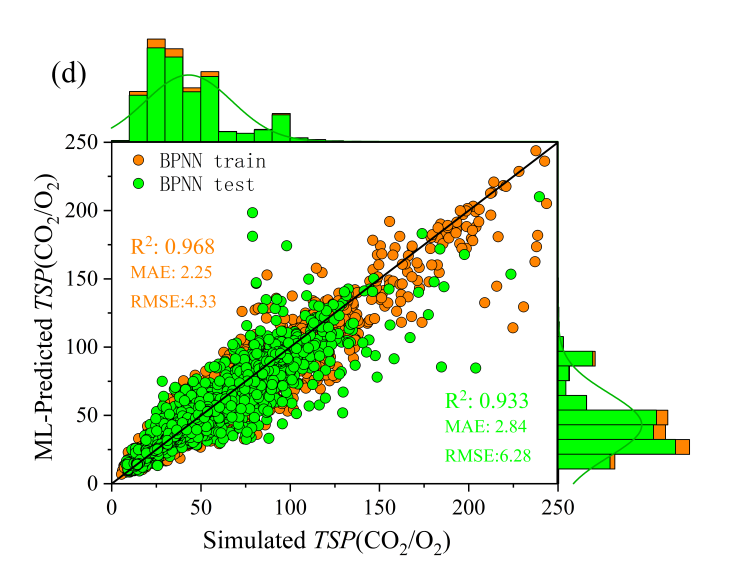

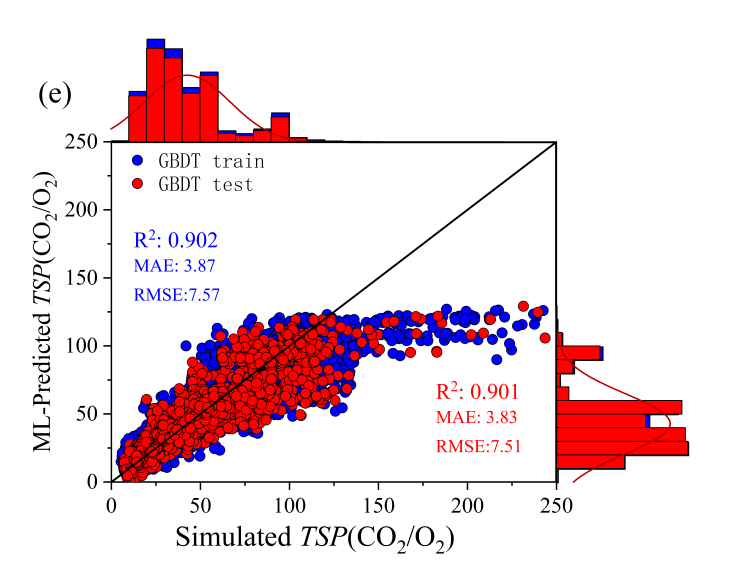

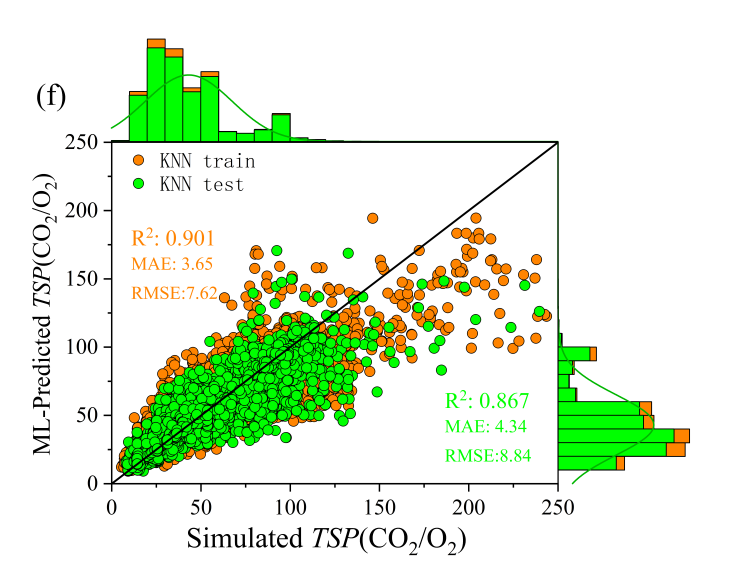
**

**Figure S17.** The prediction results of six ML algorithms for MMM's ***TSP*(CO_2_/O_2_)**. (a) XGB, (b) RF, (c) ET, (d) BPNN, (e) GBDT, and (f) KNN. The color of the dots represents different dataset. Blue (orange) symbols represent training data, and red (green) symbols represent testing data.

^
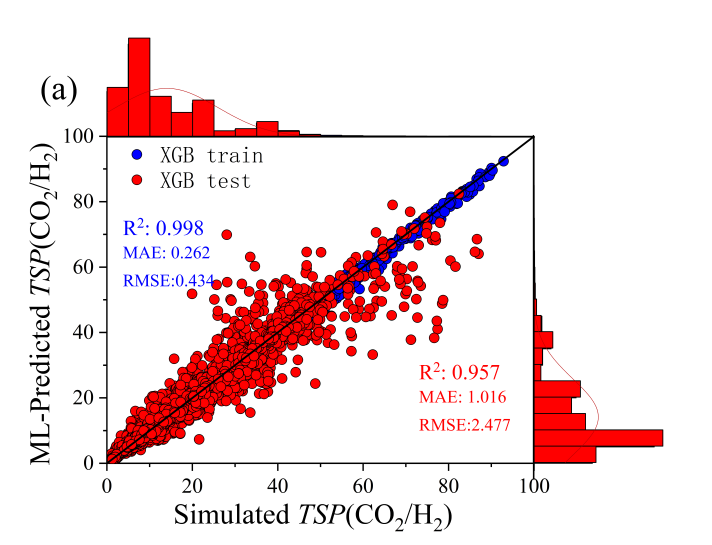

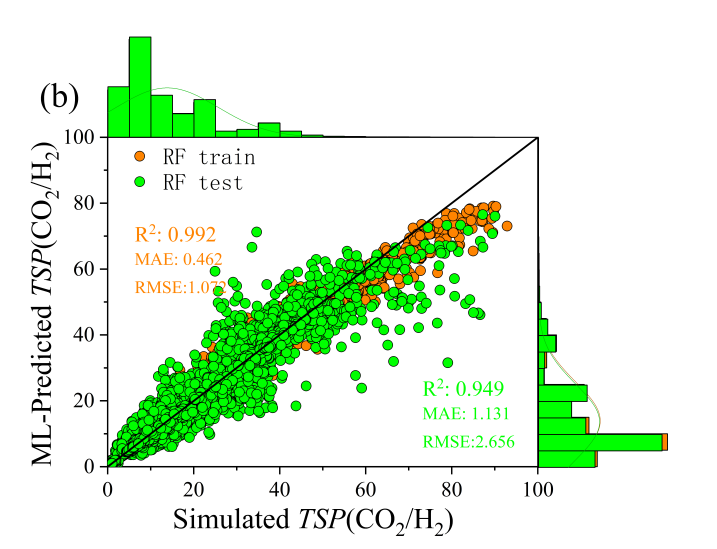
^
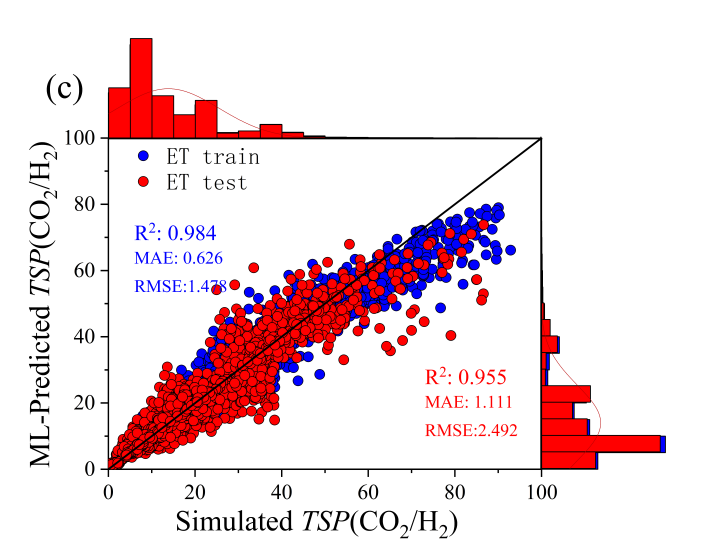
**
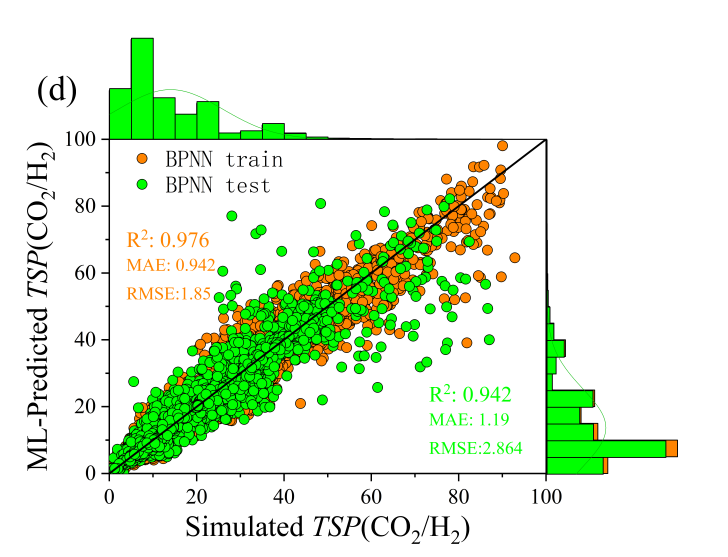
**^
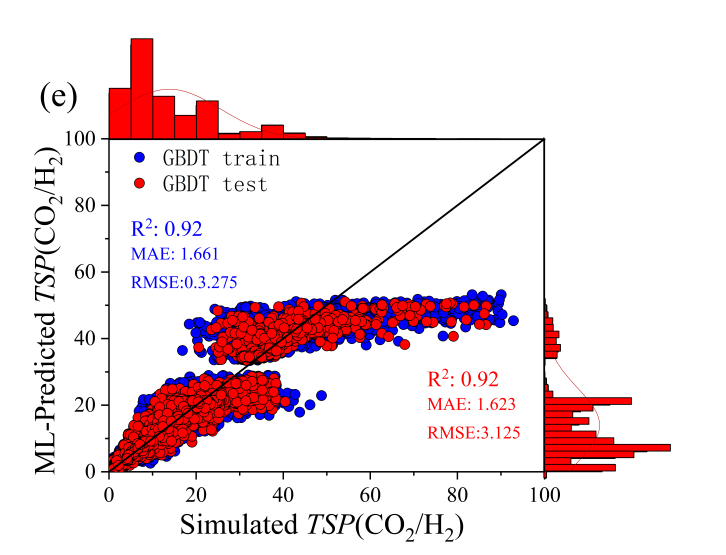

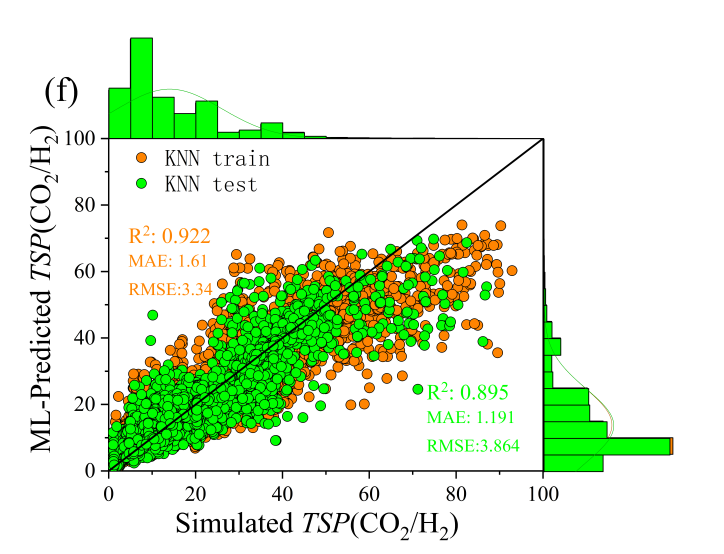
^

**Figure S18.** The prediction results of six ML algorithms for MMM's ***TSP*(CO_2_/H_2_).** (a) XGB, (b) RF, (c) ET, (d) BPNN, (e) GBDT, and (f) KNN. The color of the dots represents different dataset. Blue (orange) symbols represent training data, and red (green) symbols represent testing data.

^
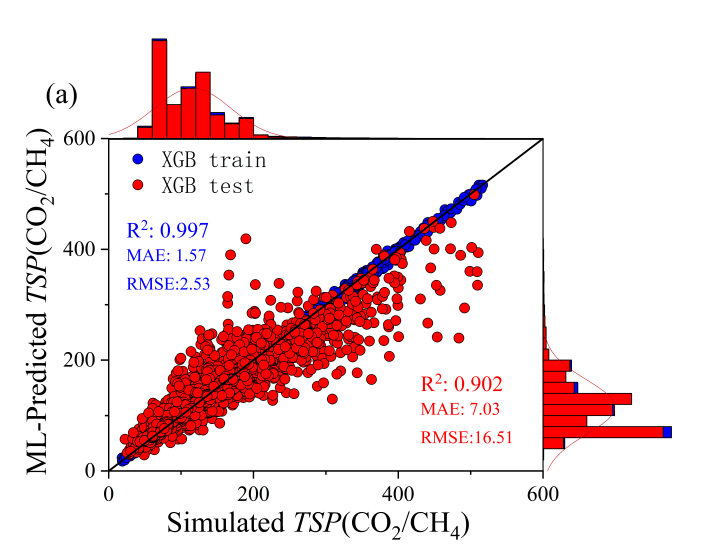

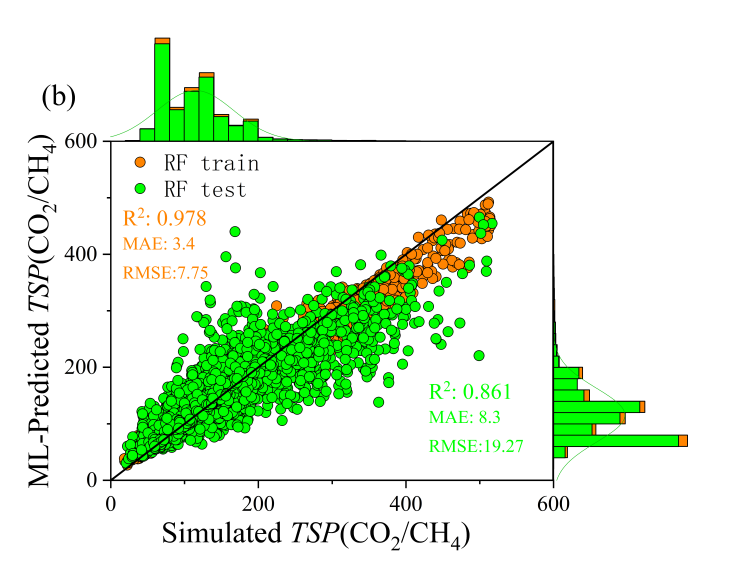
^


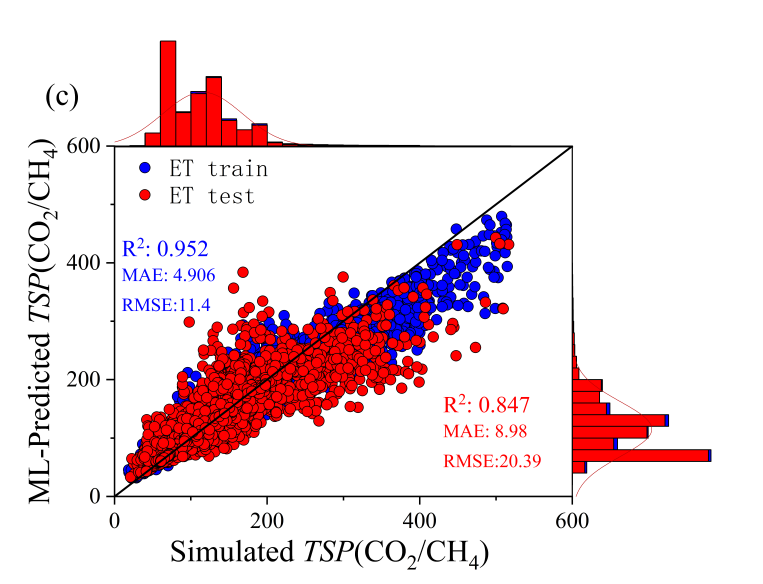
^
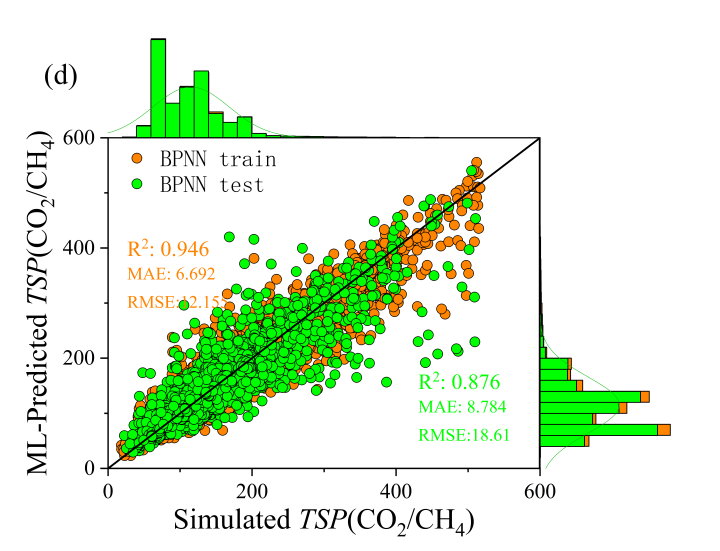
^

^
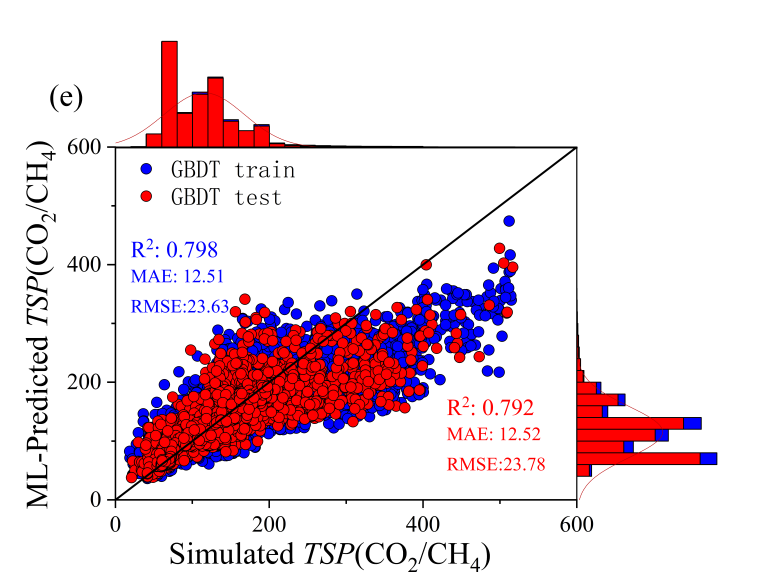

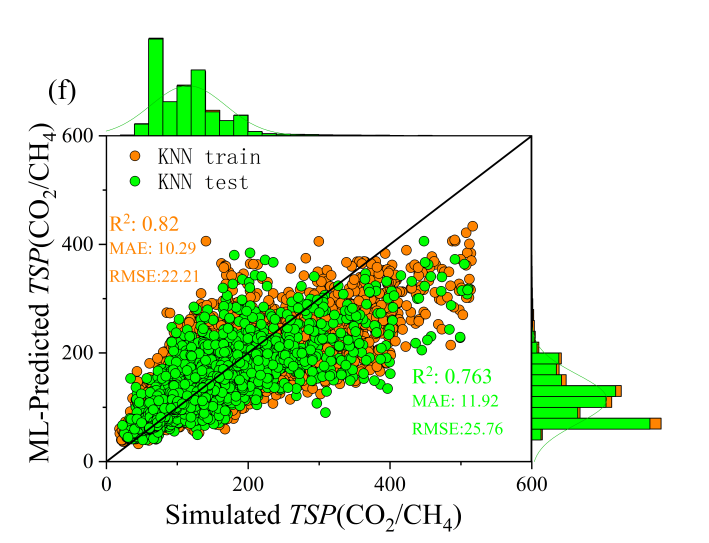
^

**Figure S19.** The prediction results of six ML algorithms for MMM's ***TSP*(CO_2_/CH_4_).** (a) XGB, (b) RF, (c) ET ,(d) BPNN, (e) GBDT, and (f) KNN. The color of the dots represents different dataset. Blue (orange) symbols represent training data, and red (green) symbols represent testing data.

**
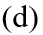
**^
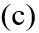
^**
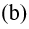

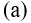
**^
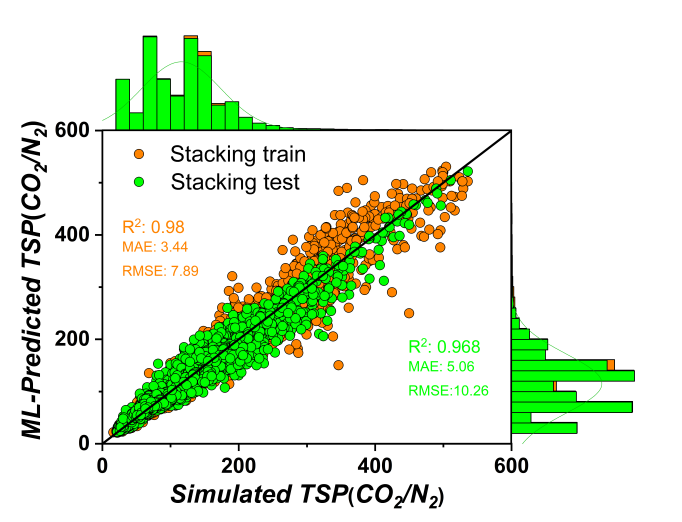

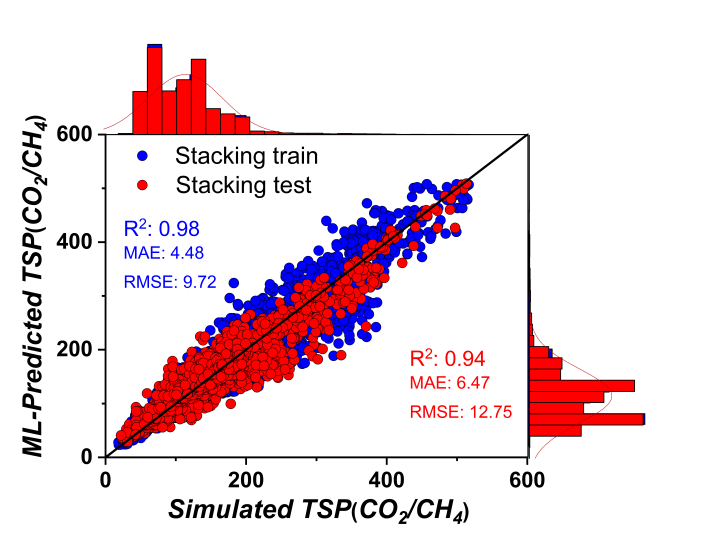

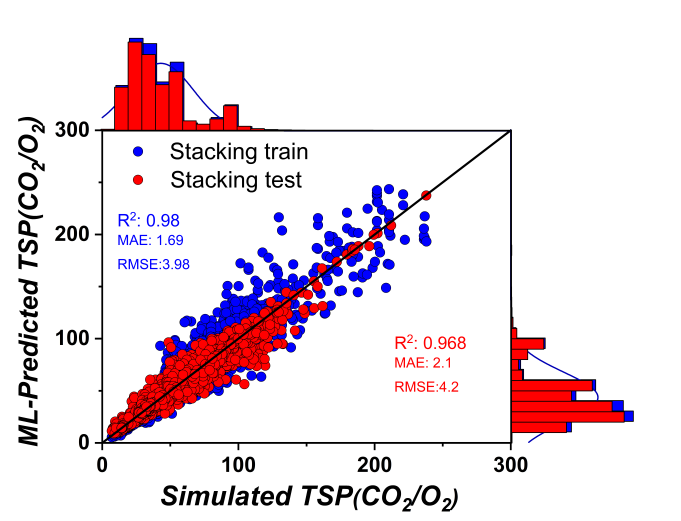

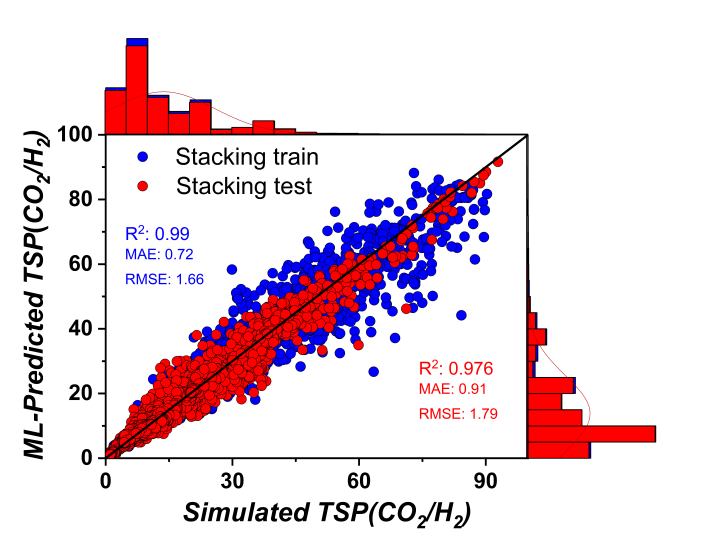
^

**
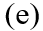
**^
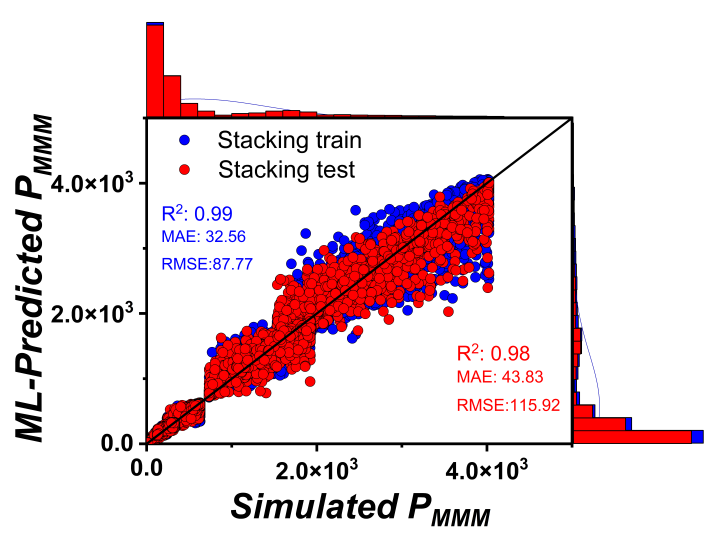
^

**Figure S20.** Predictive results of the stacking model for the performance of MMM in terms of (a) *TSP*(CO_2_/N_2_) ,(b)*TSP*(CO_2_/CH_4_), (c) *TSP*(CO_2_/O_2_), (d) *TSP*(CO_2_/H_2_), (e)*PCO_2_*. The color of the points represents different dataset, with blue symbols indicating training data and red symbols indicating testing data.

**^Section S8.^** **^Analysis of the relative importance of features^**

In this work, predictions from an ML model were interpreted using Tree Explainer in SHAP (Shapley Additive explanation). The RF model and SHAP values are combined to evaluate each feature's relative importance (degree of influence on model output). The mean of absolute SHAP value per feature across the dataset represents the importance of feature influence. The results are shown in Tables S7- S10.

**Table S7.** Importance ranking of features Based on *TSP (CO_2/_N_2_)*

| No. | 1 | 2 | 3 | 4 | 5 | 6 | 7 | 8 | 9 | 10 | 11 |
| --- | --- | --- | --- | --- | --- | --- | --- | --- | --- | --- | --- |
| Feature | LCD | HVF | VSA | PLD | LCD/PLD | PSD% | Q_st_ | *ρ* | *ρ*(MOF/poly） | *ρ*_poly_ | FFV |
| Importance (%) | 2.2 | 2.9 | 1.1 | 2.8 | 1.1 | 0.9 | 3.1 | 1.1 | 1 | 53.8 | 30 |

**Table S8.** Importance ranking of features Based on *TSP (CO_2/_CH_4_)*

| No. | 1 | 2 | 3 | 4 | 5 | 6 | 7 | 8 | 9 | 10 | 11 |
| --- | --- | --- | --- | --- | --- | --- | --- | --- | --- | --- | --- |
| Feature | LCD | HVF | VSA | PLD | LCD/PLD | PSD% | Q_st_ | *ρ* | *ρ*(MOF/poly） | *ρ*_poly_ | FFV |
| Importance (%) | 3.5 | 3.3 | 2.6 | 6.5 | 1.4 | 1.2 | 3.1 | 1.3 | 1.3 | 11.4 | 64.4 |

**Table S9.** Importance ranking of features Based on *TSP (CO_2/_O_2_)*

| No. | 1 | 2 | 3 | 4 | 5 | 6 | 7 | 8 | 9 | 10 | 11 |
| --- | --- | --- | --- | --- | --- | --- | --- | --- | --- | --- | --- |
| Feature | LCD | HVF | VSA | PLD | LCD/PLD | PSD% | Q_st_ | *ρ* | *ρ*(MOF/poly） | *ρ*_poly_ | FFV |
| Importance (%) | 13.9 | 5.9 | 2.9 | 6.6 | 2.8 | 2.2 | 6.5 | 2.4 | 2.8 | 21 | 33 |

**Table S10.** Importance ranking of features Based on *TSP (CO_2/_H_2_)*

| No. | 1 | 2 | 3 | 4 | 5 | 6 | 7 | 8 | 9 | 10 | 11 |
| --- | --- | --- | --- | --- | --- | --- | --- | --- | --- | --- | --- |
| Feature | LCD | HVF | VSA | PLD | LCD/PLD | PSD% | Q_st_ | *ρ* | *ρ*(MOF/poly） | *ρ*_poly_ | FFV |
| Importance (%) | 2 | 1.6 | 2.2 | 2 | 1.3 | 0.7 | 1.4 | 0.6 | 0.6 | 53.9 | 33.7 |

**Shapley additive explanation**

In this work, SHAP (Shapley Additive explanations) is used to explain the importance and role of different predictors in the analysis. As a game theory approach, SHAP interprets the predicted values of the model as the sum of the imputed values for each input feature, and when approximating the original model f for a particular input x, the explanation’s attribution values $\varphi_{i}$ for each feature i should sum up to the output f(x), represented by equation (11):

$f\left( x \right)=\varphi_{0}\left( f \right)+\sum_{i=0}^{n} \varphi_{i}(f,x) (11)$
 Where the sum of the feature attributes *φ*_i_ (f,x) matches the output f(x) of the original model, M is the total number of input features, $\varphi_{0}$represents the expected value when all inputs are missing, and $\varphi_{i}$ is a measure of the contribution of a given feature i to the prediction. According to game theory, the Shapley value is the only criterion that satisfies local accuracy, missing, and consistency. They are also very intuitive because they use the same units as the model output (TSP in this work). SHAP value is the Shapley value of a conditional expectation function f(x), which can be derived from equation (12):

$$\varphi_{i}=\sum_{R\inＲ} \frac{1}{M!}\left[ f_{x}\left( P_{i}^{R}\cup i \right)-f_{x}\left( P_{i}^{R} \right) \right] (12)$$

where R is the set of all feature orderings, $P_{i}^{R}$ is the set of all features that come before feature i in ordering R, and M is the number of input features for the model. For tree-based models, the study's algorithm, Tree Explainer, built by Lundberg et al., **^[55]^** effectively computes this value. The Tree Explainer SHAP value is predicted individually for each sample in the dataset, and then the results of all predictions are plotted to show the global interpretation.

**Feature importance based on different datasets.**

In this study, we performed dataset partitioning based on sorted TSP value thresholds. The different dataset partitions were labeled as ALL MMM, TOP 30000, TOP 20000, TOP 10000, TOP 8000, and TOP 6000. Subsequently, feature importance analysis was conducted using the RF model. The results of this analysis are presented in Tables S11-S15.

**Table S11.** Benchmark of *TSP _i/j_* for data sets division

| Data set | *TSP* CO_2/_N_2_ | *TSP* CO_2/_O_2_ | *TSP* CO_2/_H_2_ | *TSP* CO_2/_CH_4_ |
| --- | --- | --- | --- | --- |
| Top 6000 | 182.21 | 80.71 | 31.06 | 177.41 |
| Top 8000 | 169.70 | 62.48 | 23.32 | 160.97 |
| Top 10000 | 154.00 | 54.84 | 20.67 | 149.46 |
| Top 20000 | 131.53 | 45.60 | 13.82 | 130.48 |
| Top 30000 | 107.17 | 34.79 | 8.45 | 102.48 |
| All MMM | — | — | — | — |

| Features | LCD | HVF | VSA | PLD | LCD/PLD | PSD% | Q_st_ | *ρ* | *ρ*(MOF/poly） | *ρ*_poly_ | FFV |
| --- | --- | --- | --- | --- | --- | --- | --- | --- | --- | --- | --- |
| ALLMMM | 3.50% | 3.30% | 2.60% | 6.50% | 1.40% | 1.20% | 3.10% | 1.30% | 1.30% | 11.40% | 64.40% |
| TOP30000 | 10.00% | 8.80% | 7.40% | 21.90% | 3.60% | 2.70% | 9.00% | 3.40% | 3.10% | 2.60% | 27.30% |
| TOP20000 | 9.40% | 10.70% | 8.40% | 21.30% | 4.60% | 4.00% | 11.20% | 3.70% | 3.60% | 2.90% | 20.30% |
| TOP10000 | 9.00% | 15.00% | 6.50% | 21.90% | 7.20% | 6.10% | 14.40% | 5.10% | 5.50% | 1.40% | 7.60% |
| TOP8000 | 8.40% | 15.80% | 5.80% | 21.00% | 7.50% | 6.00% | 14.90% | 4.70% | 6.50% | 2.20% | 7.10% |
| TOP6000 | 7.70% | 14.40% | 6.00% | 20.20% | 6.90% | 6.20% | 16.30% | 5.30% | 8.40% | 2.80% | 5.80% |

**Table S12.** Importance different datasets of features (Based on *TSP* CO_2/_N_2_)

| Features | LCD | HVF | VSA | PLD | LCD/PLD | PSD% | Q_st_ | *ρ* | *ρ*(MOF/poly） | *ρ*_poly_ | FFV |
| --- | --- | --- | --- | --- | --- | --- | --- | --- | --- | --- | --- |
| ALLMMM | 2.20% | 2.90% | 1.10% | 2.80% | 1.10% | 0.90% | 3.10% | 1.10% | 1.00% | 53.80% | 30.00% |
| TOP30000 | 3.50% | 5.00% | 2.20% | 4.70% | 1.90% | 1.70% | 6.00% | 1.90% | 1.80% | 43.90% | 27.40% |
| TOP20000 | 4.00% | 6.00% | 2.00% | 5.00% | 2.00% | 2.00% | 8.00% | 3.00% | 3.00% | 11.00% | 54.00% |
| TOP10000 | 6.60% | 10.50% | 3.50% | 9.50% | 4.10% | 3.60% | 13.00% | 4.60% | 4.70% | 12.30% | 27.60% |
| TOP8000 | 9.40% | 13.40% | 4.60% | 13.70% | 5.50% | 5.80% | 15.90% | 5.20% | 9.20% | 8.30% | 9.00% |
| TOP6000 | 12.30% | 12.60% | 5.60% | 17.90% | 6.30% | 5.00% | 10.90% | 5.20% | 10.20% | 6.60% | 7.40% |

**Table S13.** Importance different datasets of features (Based on *TSP* CO_2/_CH_4_)

**Table S14.** Importance different datasets of features (Based on *TSP* CO_2/_O_2_)

| Features | LCD | HVF | VSA | PLD | LCD/PLD | PSD% | Q_st_ | *ρ* | *ρ*(MOF/poly） | *ρ*_poly_ | FFV |
| --- | --- | --- | --- | --- | --- | --- | --- | --- | --- | --- | --- |
| ALLMMM | 13.90% | 5.90% | 2.90% | 6.60% | 2.80% | 2.20% | 6.50% | 2.40% | 2.80% | 21.00% | 33.00% |
| TOP30000 | 21.30% | 9.00% | 4.90% | 8.30% | 4.90% | 4.20% | 10.10% | 3.80% | 6.20% | 21.60% | 5.70% |
| TOP20000 | 19.60% | 10.00% | 5.40% | 8.30% | 5.00% | 4.40% | 10.50% | 5.00% | 11.30% | 14.30% | 6.20% |
| TOP10000 | 23.00% | 10.90% | 6.30% | 8.80% | 5.50% | 4.70% | 11.40% | 7.10% | 9.50% | 6.20% | 6.60% |
| TOP8000 | 24.20% | 10.70% | 6.60% | 8.30% | 5.50% | 4.60% | 11.40% | 6.00% | 11.20% | 6.40% | 5.10% |
| TOP6000 | 22.50% | 7.30% | 16.40% | 6.00% | 4.50% | 4.40% | 12.70% | 4.80% | 12.30% | 5.10% | 4.00% |

**Table S15.** Importance different datasets of features (Based on *TSP* CO_2/_H_2_)

| Features | LCD | HVF | VSA | PLD | LCD/PLD | PSD% | Q_st_ | *ρ* | *ρ*(MOF/poly） | *ρ*_poly_ | FFV |
| --- | --- | --- | --- | --- | --- | --- | --- | --- | --- | --- | --- |
| ALLMMM | 2.00% | 1.60% | 2.20% | 2.00% | 1.30% | 0.70% | 1.40% | 0.60% | 0.60% | 53.90% | 33.70% |
| TOP30000 | 3.50% | 2.60% | 3.60% | 3.20% | 2.70% | 1.20% | 2.50% | 1.00% | 1.10% | 41.40% | 37.20% |
| TOP20000 | 3.70% | 3.20% | 4.80% | 5.00% | 4.00% | 1.80% | 3.20% | 1.20% | 1.20% | 19.00% | 52.90% |
| TOP10000 | 4.80% | 5.70% | 9.20% | 9.50% | 7.70% | 2.80% | 5.00% | 2.20% | 2.20% | 36.40% | 14.50% |
| TOP8000 | 7.60% | 7.50% | 14.70% | 11.70% | 11.40% | 3.90% | 6.00% | 3.00% | 3.10% | 19.30% | 11.80% |
| TOP6000 | 6.10% | 11.60% | 13.10% | 31.80% | 7.40% | 4.70% | 6.70% | 3.30% | 3.20% | 6.00% | 6.10% |

^
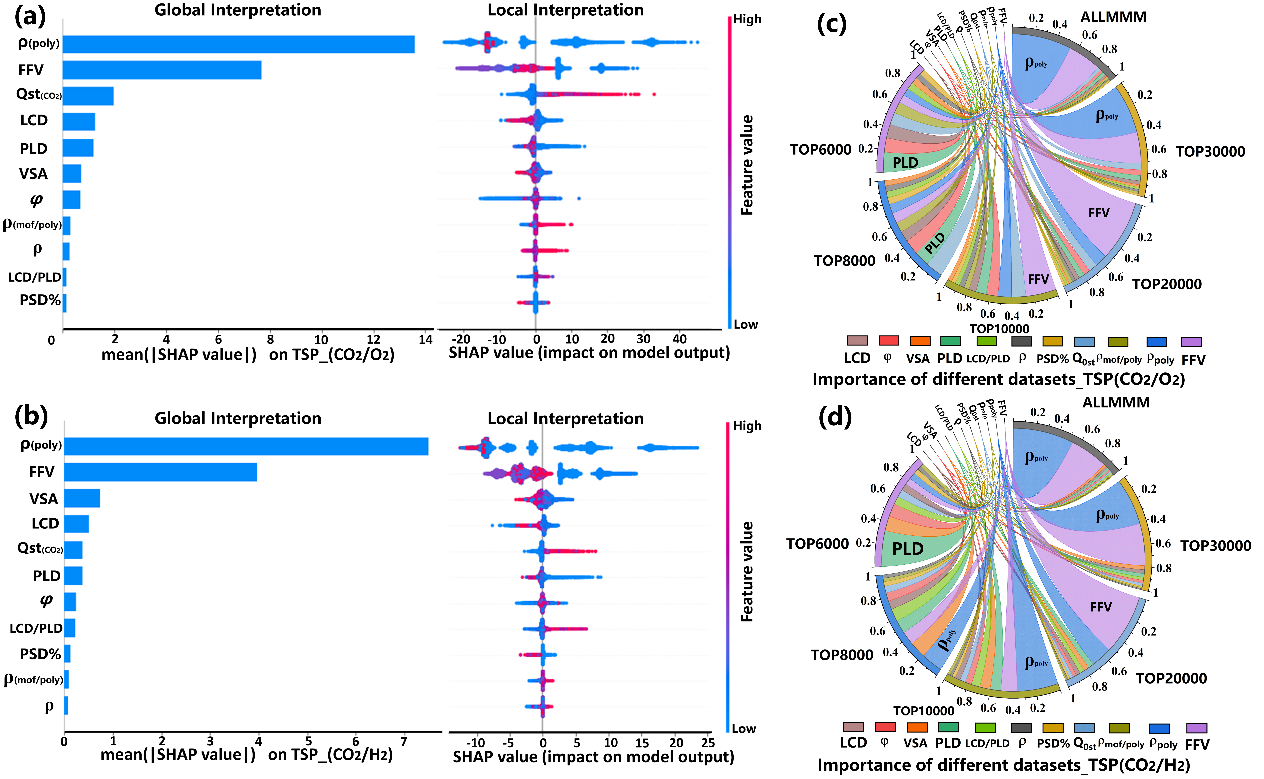
^

**Figure S21.** (a), (b) Global feature importance (left, bar chart) and local explanation plot (right, beeswarm plot) for predicting *TSP*CO_2_/O_2_ and *TSP*CO_2_/H_2_ based on the RF model, where dots represent individual data points in the study. Note: The points on the x-axis show how each data point influences the model's prediction for each feature, and when multiple points are at the same x location, they display density (colors represent feature values, where red dots indicate high, and blue dots indicate low; positive SHAP values represent positive feature impacts on prediction, negative SHAP indicates negative feature impacts on prediction). (c), (d) Relative feature importance for predicting performance *TSP*CO_2_/O_2_ and *TSP*CO_2_/H_2_ based on the RF model using different TOP datasets, with different colors representing different features. The feature importance ranking for each dataset is arranged clockwise, and the features marked in bold in the figure are the features with the highest importance proportion in the dataset.

**Section S9.** **Top-performance MMMs and MOFs.**

The top 10 MMMs with the best CO_2_ separation performance were selected, and the results are listed in Table S16.Based on the TOPMMM for each binary gas mixture separation, we further investigated the potential for gas separation of multicomponent gas mixtures in each system. Upon statistical screening of the TOPMMM for each binary gas mixture, we found that MMMs composed of PIM-1 and MOF consistently ranked among the top performers in separating CO_2_/X (N_2_, CH_4_) mixtures. Therefore, using the sum of their respective TSP values as the screening criterion, we identified the top 5 MMMs (PIM-1) for CO_2_/X (N_2_, CH_4_) mixture separation. Similarly, the top 5 MMMs(a-PEO) were selected for CO_2_/X (O_2_, H_2_) mixture separation. The results are listed in Table S16.

**Table S16.** Top-performance MMMs for the separation of different binary gas mixtures.

| Mixture of gases | Type of polymer | *P_poly_*(CO_2_) (barrer)^a^ | MOF Type code^b^ | *P*CO_2_ (barrer)^a^ | *S_perm i/j_* (barrer)^a^ |  |
| --- | --- | --- | --- | --- | --- | --- |
|  |  |  |  |  |  |  |
| CO_2_/N_2_ | PIM-1 | 2300 | MUFHUR | 4017 | 65 |  |
|  |  |  | CAHLEE | 4010 | 65 |  |
|  |  |  | NICJUG | 4013 | 64 |  |
|  |  |  | XOFTIW | 4002 | 64 |  |
|  |  |  | NORGOS | 3965 | 64 |  |
|  |  |  | WOCJII | 4024 | 64 |  |
|  |  |  | NOQPAM | 4021 | 63 |  |
|  |  |  | YUJVOQ | 4018 | 62 |  |
|  |  |  | MUFJED | 3871 | 63 |  |
|  |  |  | QAVZAQ | 3816 | 62 |  |
| CO_2_/CH_4_ | PI-5 | 190 | MUFHUR | 332 | 89 |  |
|  |  |  | MUFJED | 331 | 89 |  |
|  |  |  | QAVZAQ | 331 | 89 |  |
|  |  |  | YAFGAP | 332 | 89 |  |
|  |  |  | WENSIS | 332 | 88 |  |
|  |  |  | IDAZEU | 332 | 88 |  |
|  |  |  | ZADDAJ | 332 | 88 |  |
|  |  |  | VAZKUE | 331 | 88 |  |
|  |  |  | IJASOC | 332 | 88 |  |
|  |  |  | MUFJAZ | 330 | 88 |  |
| CO_2_/O_2_ | a-PEO | 143 | BEVQUP | 244 | 44 |  |
|  |  |  | VAZLAL | 248 | 44 |  |
|  |  |  | VAZKUE | 249 | 43 |  |
|  |  |  | MUFJED | 250 | 43 |  |
|  |  |  | MUFJAZ | 249 | 43 |  |
|  |  |  | QAVZAQ | 249 | 43 |  |
|  |  |  | LANCOU | 244 | 43 |  |
|  |  |  | MUFHUR | 250 | 42 |  |
|  |  |  | SUHHOT | 233 | 42 |  |
|  |  |  | QAVYUJ | 242 | 42 |  |
| CO_2_/H_2_ | a-PEO | 143 | MUFJAZ | 249 | 17 |  |
|  |  |  | WEWGAH | 240 | 16 |  |
|  |  |  | POWBIO | 248 | 16 |  |
|  |  |  | LANCOU | 244 | 16 |  |
|  |  |  | DUQCAU | 248 | 16 |  |
|  |  |  | QAVZAQ | 249 | 16 |  |
|  |  |  | QOVXEG02 | 241 | 16 |  |
|  |  |  | AWEDUC | 235 | 16 |  |
|  |  |  | ECUCOV | 244 | 16 |  |
|  |  |  | XEJJOM | 243 | 16 |  |

**Table S17.** Top-performance MMMs for the separation of different binary gas mixtures.

| mixture of gases | Type of polymer | *P_poly_* (CO_2_) barrer^a^ | MOF Type code^b^ | *P*CO_2_ barrer^a^ | *S*_perm_ (CO_2_/O_2_) | *S*_perm_ (CO_2_/H_2_) |
| --- | --- | --- | --- | --- | --- | --- |
| CO_2_/N_2_, CO_2_/CH_4_ | PIM-1 | 2300 | MUFHUR | 4016.9 | 64.59 | 48.2 |
|  |  |  | CAHLEE | 4009.86 | 64.59 | 48.08 |
|  |  |  | WOCJII | 4024.09 | 63.53 | 48.21 |
|  |  |  | XOFTIW | 4001.52 | 63.66 | 47.91 |
|  |  |  | NICJUG | 4012.95 | 63.79 | 47.6 |
| mixture of gases | Type of polymer | *P_poly_* (CO_2_) barrer^a^ | MOF Type code^b^ | *P*CO_2_ barrer^a^ | *S*_perm_ (CO_2_/O_2_) | *S*_perm_ (CO_2_/H_2_) |
| CO_2_/O_2_, CO_2_/H_2_ | a-PEO | 140 | MUFJAZ | 248.68 | 43.13 | 16.84 |
|  |  |  | QAVZAQ | 249.37 | 43.06 | 16.23 |
|  |  |  | LANCOU | 244.15 | 43.12 | 16.33 |
|  |  |  | QAVYUJ | 241.75 | 41.56 | 15.9 |
|  |  |  | SUHHOT | 232.8 | 42.33 | 15.03 |

*^a^* 1 barrer = 3.348 × 10^–16^ mol m (m^2^ s Pa)^-1^ = 10^−10^ cm^3^ (STP) cm (cm^2^·s·cmHg) ^-1^

*^b^* CCDC code

**Table S18**.CO_2_/CH_4_ and CO_2_/N_2_ separation data for selected MOFs based MMMs reported in literature

| **MMMs** | **Loading** | **Pressure** | **Temperature** | ***P*CO_2_** | ***S_perm_CO_2_/CH_4_*** | **Ref.** |
| --- | --- | --- | --- | --- | --- | --- |
|  | **(wt.%)** | **(bar)** | **(℃)** | **(barrer)** |  |  |
| MOF-5/Matrimid | 30 | 2 | 35 | 20.2 | 44.7 | **[56]** |
| Cu-BTC/PPO | 40 | 2 | 30 | 113.7 | 33.5 | **[57]** |
| MIL-53/Matrimid | 37.5 | 2 | 35 | 40 | 90.1 | **[58]** |
| MIL-53/Matrimid | 15 | 3 | 35 | 12.43 | 51.8 | **[59]** |
| NH2-MIL-53/6FDA-ODA | 32 | 10 | 35 | 14.6 | 78 | **[60]** |
| ZIF-8/Matrimid | 50 | 2.7 | 35 | 4.7 | 125 | **[61]** |
| ZIF-71/6FDA-Durene | 10 | 3.5 | 35 | 1606 | 20 | **[62]** |
| ZIF-8/PIM-1 | 43 | 1 | 20 | 6300 | 14.7 | **[63]** |
| IL@ZIF-8/Pebax 1657 | 15 | 1 | 25 | 104.9 | 34.8 | **[64]** |
| ZIF-90/6FDA-DAM | 15 | 2 | 25 | 800 | 26.6 | **[65]** |
| UiO-66/6FDA-ODA | 25 | 10 | 35 | 50.4 | 46.1 | **[66]** |
| ns-CuBDC/Matrimid | 8.2 | 3 | 25 | 4.09 | 78.7 | **[67]** |
| **MMMs** | **Loading** | **Pressure** | **Temperature** | ***P*CO_2_** | ***S_perm_CO_2_/N_2_*** | **Ref.** |
|  | **(wt.%)** | **(bar)** | **(℃)** | **(barrer)** |  |  |
| UiO-66(5%) /PIM-1 | ‒ | ‒ | ‒ | 5340 | 21.3 | **[68]** |
| ZIF-7/Pebax | ‒ | ‒ | ‒ | 145 | 97 | **[69]** |
| ZIF-8/SEBS | ‒ | ‒ | ‒ | 454.6 | 12 | **[70]** |
| ZIF-8/Pebax 2533 | ‒ | ‒ | ‒ | 1287 | 32.3 | **[71]** |
| [ZIF-8@GO/Pebax](mailto:ZIF-8@GO/Pebax) | ‒ | ‒ | ‒ | 249 | 47.6 | **[72]** |
| UiO-66/PSF | 20 | 3 | 35 | 16 | 26.2 | **[73]** |
| UiO-66/PIM-1 | 23.1 | 1 | 25 | 7610 | 20.7 | **[74]** |
| Azo-UiO-66/Matrimid | 10 | 4 | 37 | 10 | 37 | **[75]** |
| UiO-66/PEBA | 10 | ‒ | 25 | 139.7 | 61.1 | **[76]** |
| MOF801/PIM-1 | 5 | 4 | 35 | 9686 | 27 | **[77]** |
| HKUST-1/6FDA-Durene | 13.8 | 2 | 25 | 1101.6 | 27.1 | **[78]** |
| MIL-101(Cr)/PIM-1 | 5 | 3.04 | 25 | 2979 | 37 | **[79]** |

Table S18 presents the performance of MMMs reported in the literature. Since the separation indicators (TSP) for different binary gases are not directly comparable, we first normalized the four TSP values and then calculated their sum (*TSP_sum_*) as the screening criterion. This allowed us to identify the top 10 optimal MOFs, which are listed in Table S19. These MOFs are not only suitable for the separation of multiple gases but also enhance the separation performance of polymers.

**Table S19.** Top-performance CoRE-MOFMs for multiple gas binary mixtures

| No. | CSD code^b^ | LCD（Å） | PLD（Å） | LCD/PLD | VSA | Q_st_ | *ρ*（kg/m^3^） | *P*CO_2_ (barrer)^a^ | *S*_perm_ (CO_2_/N_2_) | *S*_perm_ (CO_2_/O_2_) | *S*_perm_ (CO_2_/H_2_) | *S*_perm_ (CO_2_/CH_4_) | *TSP*_sum_ |
| --- | --- | --- | --- | --- | --- | --- | --- | --- | --- | --- | --- | --- | --- |
| 1 | MUFHUR | 3.42 | 2.69 | 1.27 | 0.00 | 5.59 | 2054.60 | 2114888.45 | 878840.79 | 1250020.01 | 93893.51 | 316086276.30 | 2.67 |
| 2 | CAHLEE | 3.76 | 3.12 | 1.21 | 1.06 | 4.70 | 2514.92 | 1128709.69 | 538268.44 | 328310.38 | 125662.47 | 5745395.38 | 2.67 |
| 3 | WOCJII | 4.01 | 2.57 | 1.56 | 5.53 | 1.55 | 1574.83 | 18791286.33 | 3304488.62 | 3875902.85 | 2516639.58 | 49161525.98 | 2.67 |
| 4 | NOQPAM | 3.67 | 2.68 | 1.37 | 0.55 | 1.52 | 2077.01 | 3873373.47 | 565775.73 | 373815.92 | 532456.48 | 5661190.53 | 2.66 |
| 5 | XOFTIW | 3.88 | 3.29 | 1.18 | 5.89 | 6.98 | 2438.10 | 725397.16 | 168831.61 | 73080.05 | 46015.82 | 1352218.03 | 2.66 |
| 6 | NICJUG | 4.90 | 3.36 | 1.46 | 64.35 | 1.04 | 1725.42 | 1419512.31 | 321003.20 | 93777.01 | 30759.22 | 526805.57 | 2.65 |
| 7 | MUFJAZ | 3.30 | 2.45 | 1.35 | 0.00 | 1.55 | 2135.35 | 41975.39 | 11403.28 | 39758.14 | 19061.58 | 803204.50 | 2.63 |
| 8 | QAVZAQ | 3.43 | 2.66 | 1.29 | 0.00 | 3.01 | 1880.77 | 75328.28 | 200584.29 | 66399.88 | 18690.97 | 20331891.53 | 2.62 |
| 9 | MUFJED | 3.41 | 2.67 | 1.28 | 0.00 | 1.54 | 2290.09 | 104552.83 | 66392.35 | 96313.55 | 5799.90 | 13626413.68 | 2.61 |
| 10 | LANCOU | 3.29 | 2.47 | 1.33 | 0.00 | 2.34 | 2170.93 | 10470.01 | 10747.67 | 14021.57 | 3797.06 | 1386467.96 | 2.61 |

**^a^** 1 barrer = 3.348 × 10^–16^ mol·m/ (m^2^ s Pa) = 10^−10^ cm^3^(STP)cm/ (cm^2^·s·cmHg)

**^b^** CCDC code


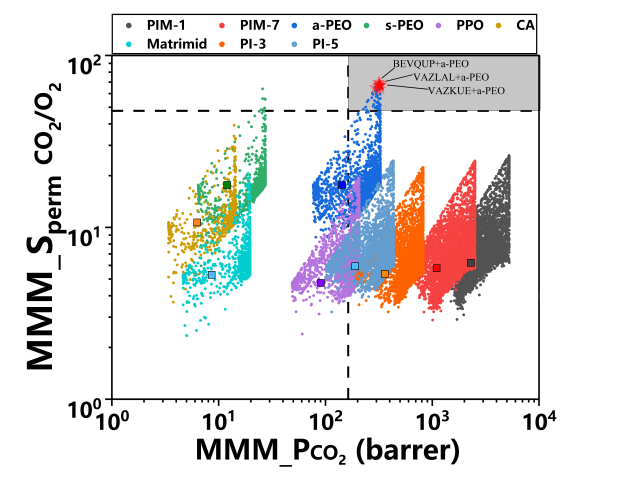

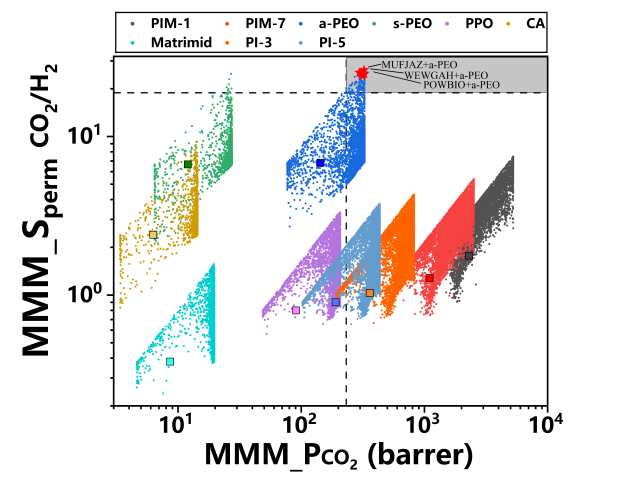


**（a）**

**（b）**

**Figure S22.** Optimal MMM for CO_2_ separation. Relationship graph between (a) *S*_perm_ CO_2_/O_2_, (b) *S*_perm_ CO_2_/H_2_, and *P*CO_2_ in MMM. The black line represents the Robeson upper bound. The properties of each neat polymer are marked with square symbols, while the predicted properties of MMMs derived from the polymer are marked with circular symbols of the same color. Red stars represent the top 10 MMMs for each gas separation.

**（b）**

**（a）**


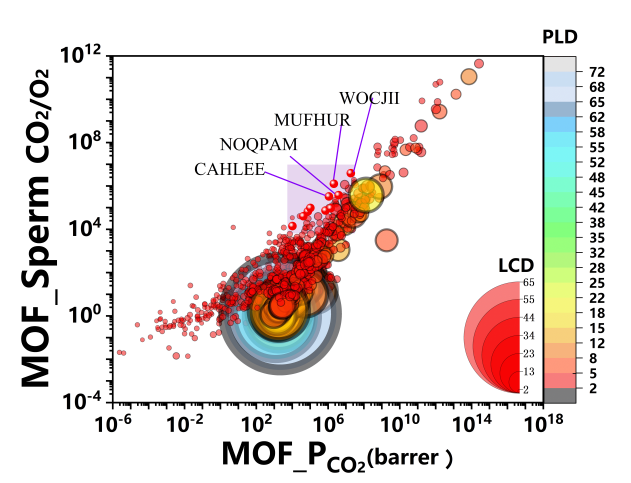

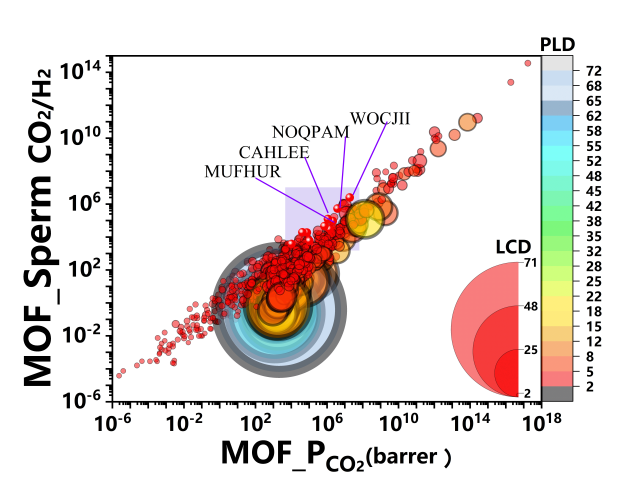


**Figure S23.** Optimal MOF for CO_2_ separation. Relationship graph between (a) *S*_perm_ CO_2_/O_2_, (b) *S*_perm_ CO_2_/H_2_, and *P*CO_2_ in MOF. The black line represents the Robeson upper bound. The purple background region indicates the distribution region of the top 10 optimal spherical MOFs.


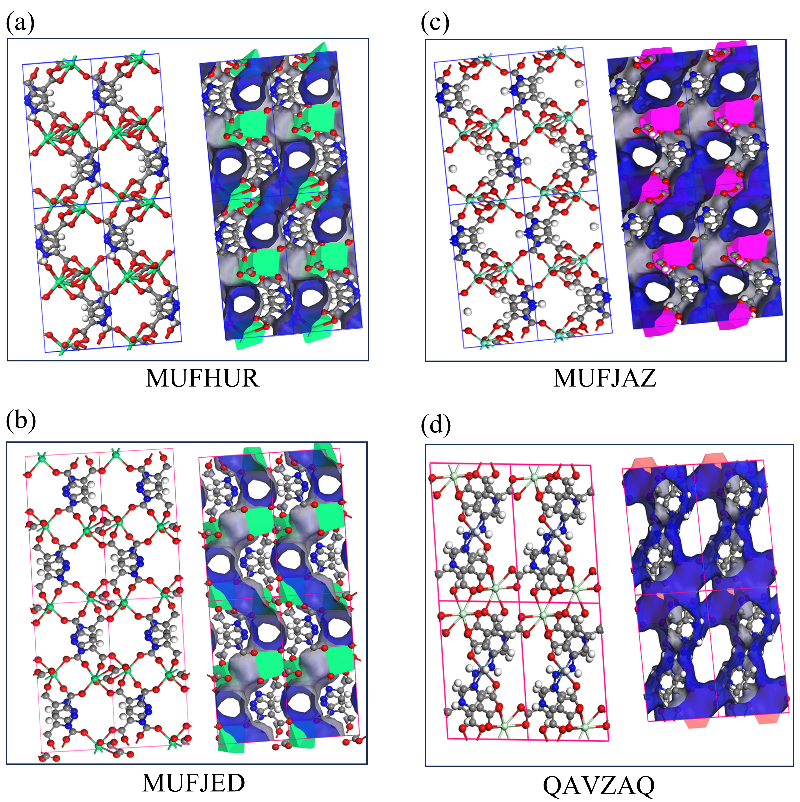

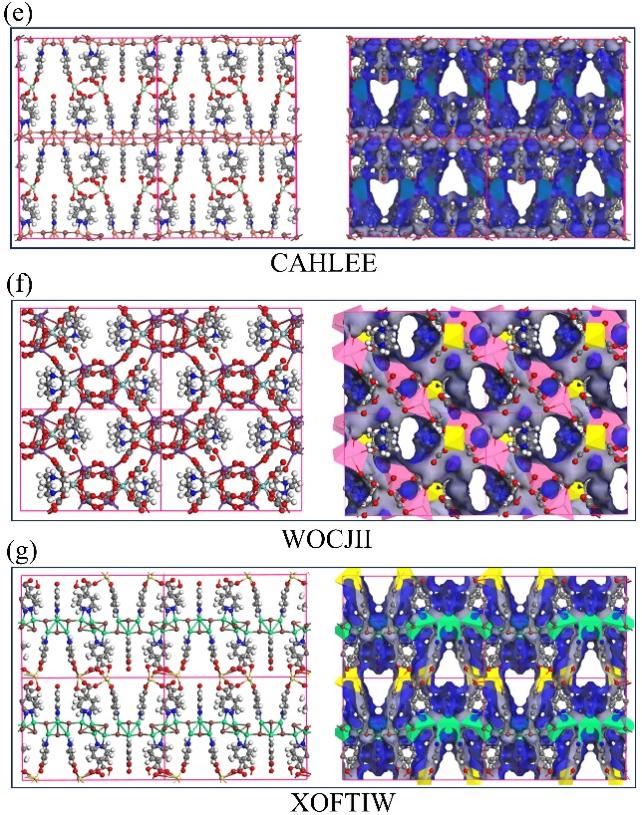


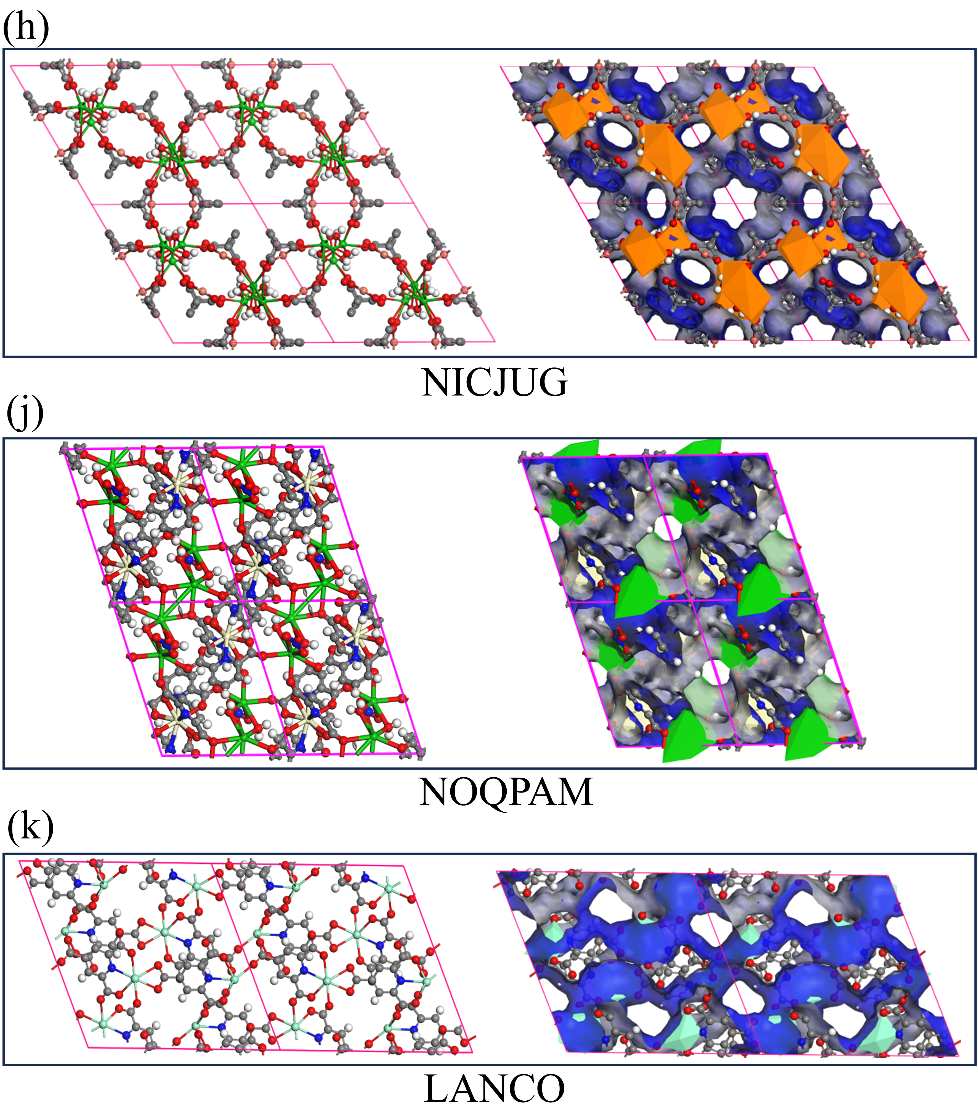


**Figure S24.** Atomistic and Pore simulation structures of 10 top-performance CoRE-MOFMs.

**Section S10.** **Relationships between MMM features and properties of gases**

The extrapolation prediction of optimal MMM features was conducted based on univariate and multivariate regression analysis.

**
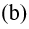

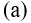

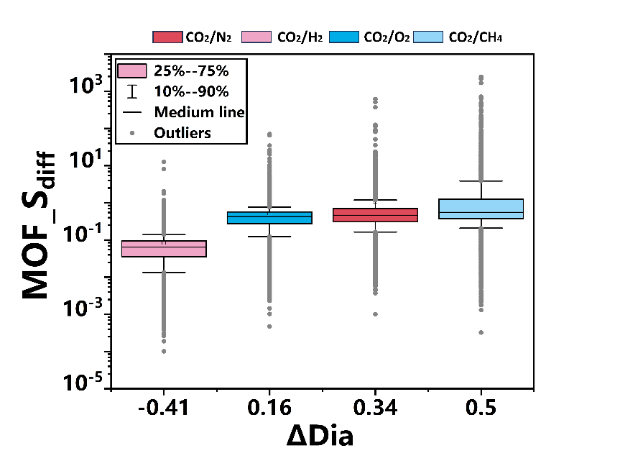

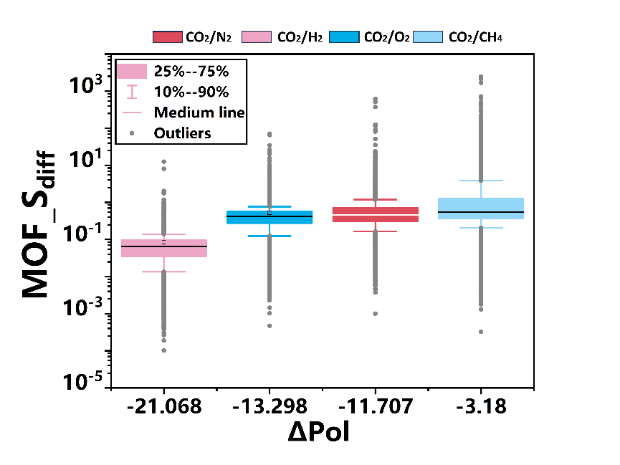
**

**Figure S25.** (a) Box and whisker plots for the gas mixture of *S*_diff_ and ∆Dia in 6013MOF (b) Box and whisker plots for the gas mixture of *S*_diff_ and ∆Pol in 6013 MOF.


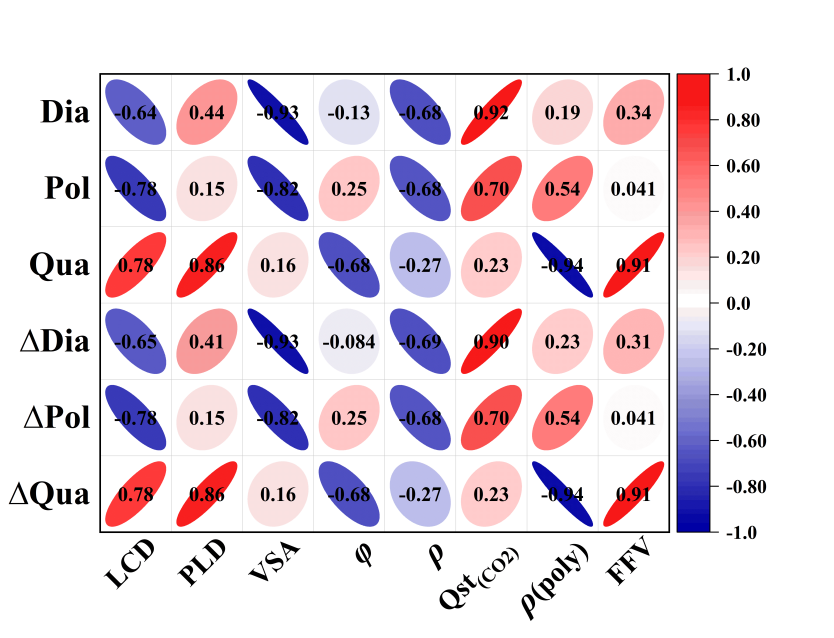


**Figure S26.** Correlation coefficient of feature and physical properties of X in CO_2_/X(X=N_2_,O_2_,H_2_,CH_4_) gas mixtures.

**
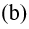

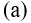
**
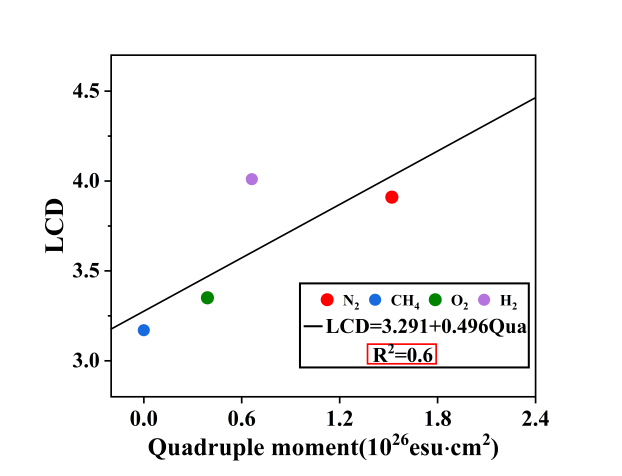

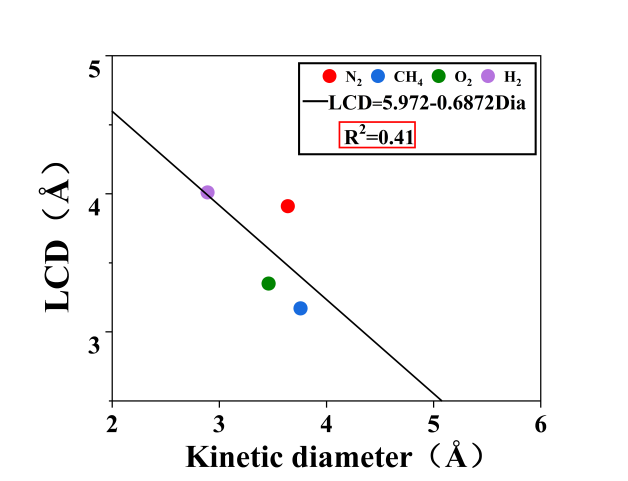


**Figure S27.** Predicted LCD by Unary regression model using (a) Qua and (b) Dia.


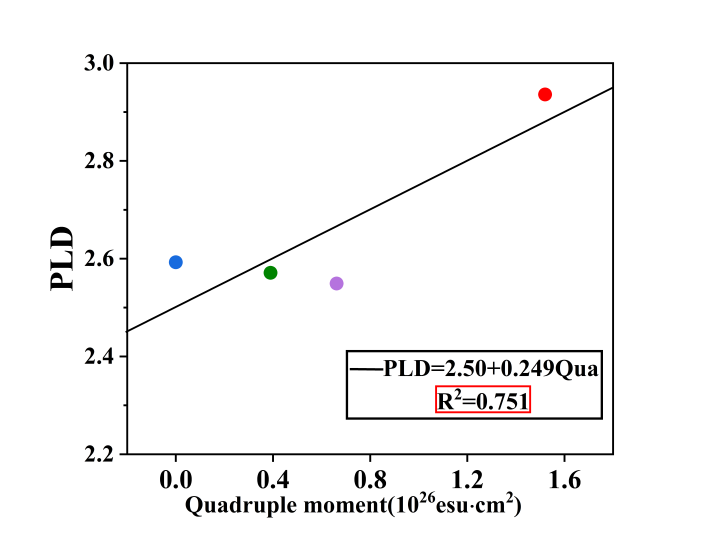


**Figure S28.** Predicted PLD by Unary regression mode using Qua.

**
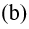

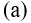
**
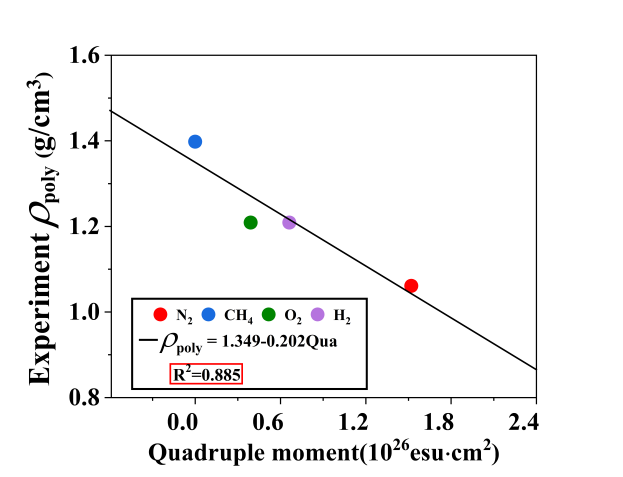

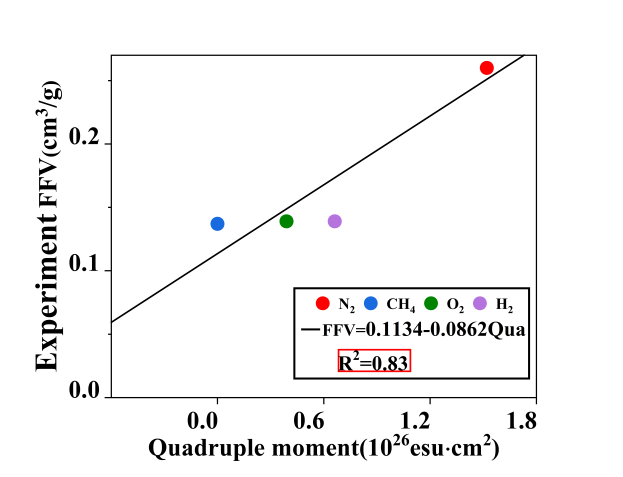


**Figure S29.** Predicted (a) *ρ*_(poly)_ and (b) FFV by Unary regression model.

**
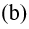

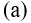
**
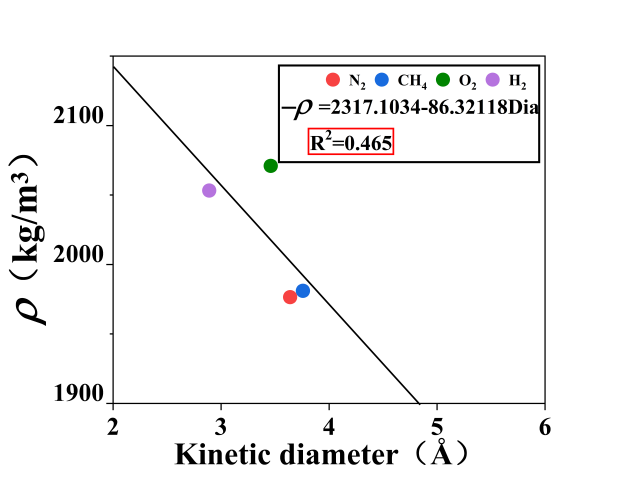

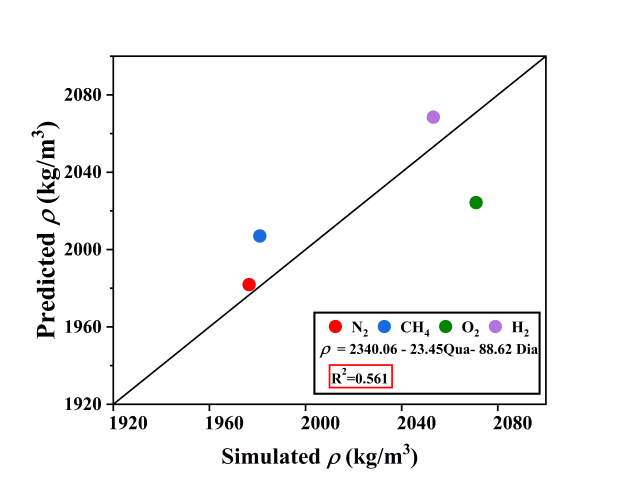


**Figure S30.** (a) Predicted *ρ* by Unary regression model. (b) Comparison between the predicted *ρ* by Multiple regression model and the simulated *ρ.*

**
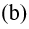

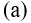

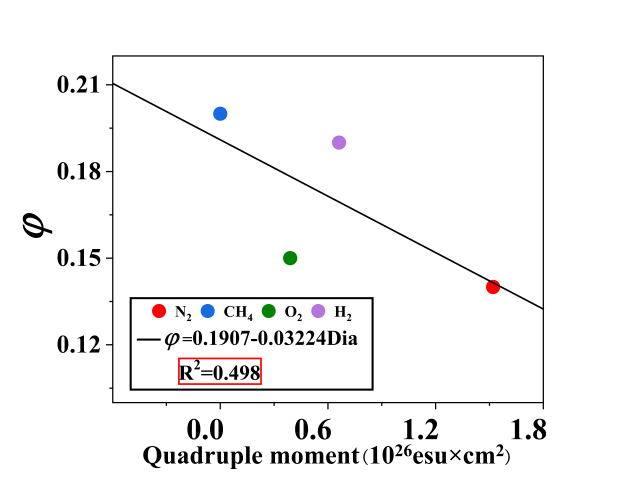

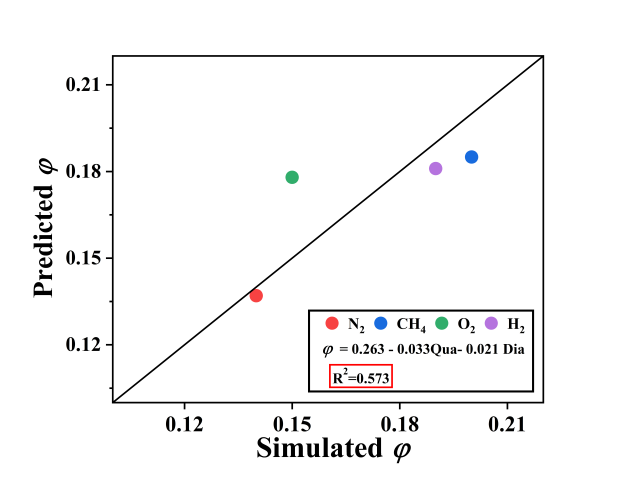
**

**Figure S31.** (a) Predicted *φ* by Unary regression model. (b) Comparison between the predicted *φ* by Multiple regression model and the simulated *φ*.

**Section S11. Transfer learning**

**
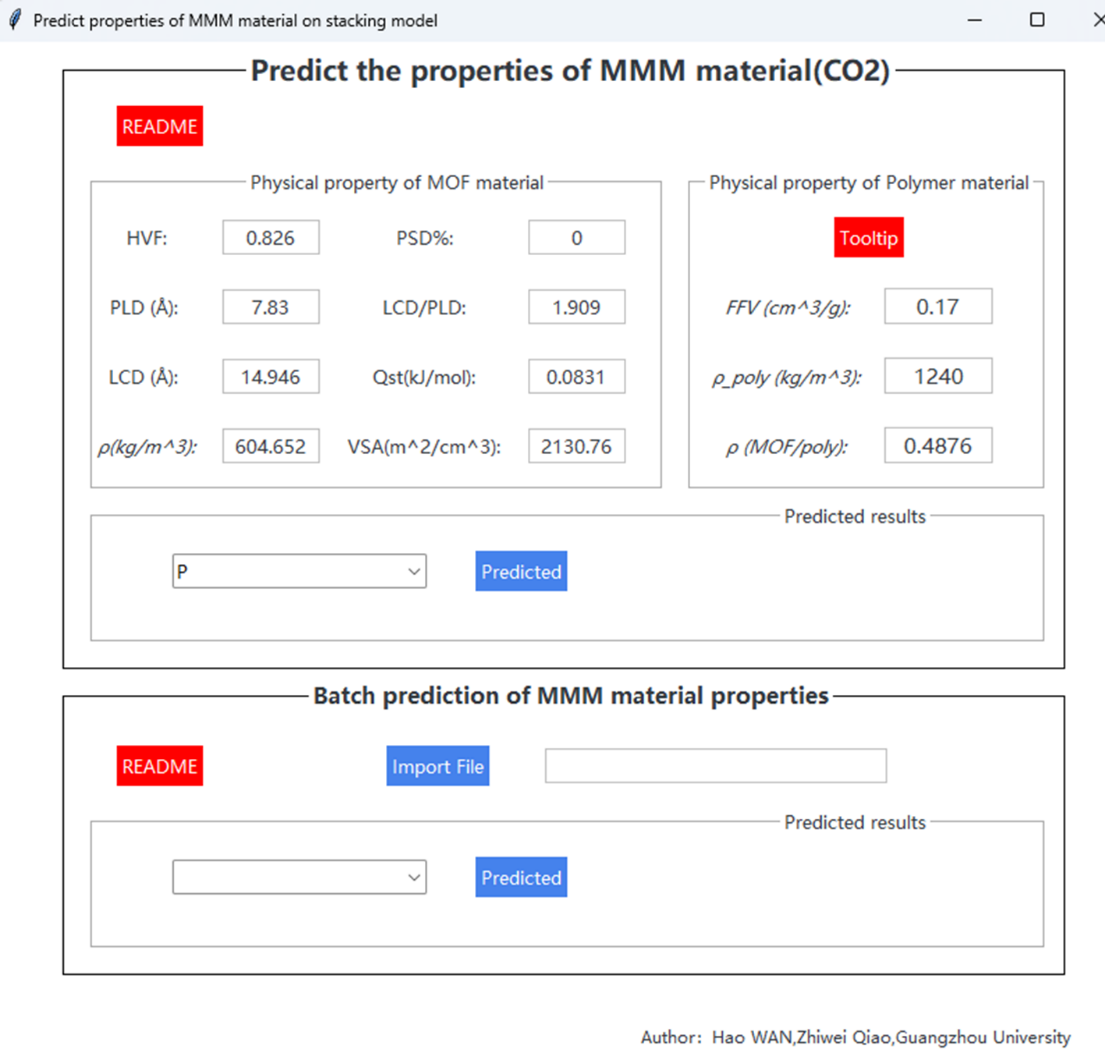
**

**Figure S32.** The main interface of the interactive desktop application for predicting the CO_2_ separation performance of MMM materials.

For the convenience of users, the Stacking model trained to predict the CO_2_ performance of mixed matrix membrane materials has been transformed into an interactive desktop application. Users do not need to install other auxiliary software, directly open main_MMM.exe to use this software.

Usage: you need to select one of the download methods to download the model folder, and then put it in the same directory as the other folders and .exe files.

This software has two functions:

1- To calculate the comprehensive separation performance (TSP) of CO_2_/X, where X = N_2_, O_2_, H_2_, CH, and the permeability (P) of CO_2_ in a single crystal material. A single prediction result is displayed on the interface.

2- Batch calculation of CO_2_ performance in MMM (Mixed Matrix Membrane) materials. The predicted result will be saved in Result/Batch_Predicted.xlsx.

Detailed instructions and a user manual are also provided on the GitHub repository at https://github.com/haowanae/Pred_MMM_CO2/tree/master.

**Table S20.** The version information of tool package for the interactive desktop application design.

|  | Package |  |  | Version |  |
| --- | --- | --- | --- | --- | --- |
|  | pandas |  |  | 1.2.4 |  |
|  | ttkbootstrap |  |  | 1.10.1 |  |
|  | joblib |  |  | 1.2.0 |  |
|  | numpy |  |  | 1.21.0 |  |
|  | pillow |  |  | 8.2.0 |  |
|  | scipy |  |  | 1.6.2 |  |
|  | xgboost |  |  | 1.6.1 |  |
|  | scikit-learn |  |  | 0.24.1 |  |
|  | pyinstaller |  |  | 6.2.0 |  |
|  | openpyxl |  |  | 3.1.2 |  |

Notes: To design the interactive desktop application, we utilize ttkbootstrap, a common Python GUI module, and pyinstaller to package exe files.

**Table S21**.The prediction results of the stacking model for the "new" MMM (polymer)

| **MMM** | | Results | *TSP* CO_2_/N_2_ | *TSP* CO_2/_O_2_ | *TSP* CO_2/_H_2_ | *TSP* CO_2/_CH_4_ |
| --- | --- | --- | --- | --- | --- | --- |
| Polymer | Data |  |  |  |  |  |
| PIM-7 | Test | R^2^ | 0.90 | 0.91 | 0.93 | 0.87 |
|  |  | MAE | 12.04 | 7.89 | 2.08 | 10.98 |
|  |  | RMSE | 17.90 | 9.08 | 3.37 | 16.59 |
|  | Train | R^2^ | 0.98 | 0.98 | 0.98 | 0.98 |
|  |  | MAE | 1.77 | 0.65 | 0.29 | 2.40 |
|  |  | RMSE | 3.83 | 1.45 | 0.60 | 5.07 |
| PIM-1 | Test | R^2^ | 0.90 | 0.72 | 0.72 | 0.90 |
|  |  | MAE | 10.97 | 22.00 | 10.99 | 11.28 |
|  |  | RMSE | 16.83 | 24.13 | 11.84 | 18.31 |
|  | Train | R^2^ | 0.98 | 0.97 | 0.97 | 0.99 |
|  |  | MAE | 1.76 | 0.63 | 0.26 | 2.32 |
|  |  | RMSE | 3.79 | 1.38 | 0.57 | 4.86 |
| Matrimind | Test | R^2^ | 0.91 | 0.77 | 0.71 | 0.92 |
|  |  | MAE | 50.60 | 10.82 | 4.69 | 66.58 |
|  |  | RMSE | 51.03 | 10.89 | 4.73 | 68.29 |
|  | Train | R^2^ | 0.98 | 0.98 | 0.97 | 0.99 |
|  |  | MAE | 1.78 | 0.82 | 2.29 | 15.64 |
|  |  | RMSE | 3.57 | 1.71 | 4.57 | 37.54 |
| CA | Test | R^2^ | 0.90 | 0.82 | 0.67 | 0.92 |
|  |  | MAE | 50.34 | 10.90 | 4.72 | 66.70 |
|  |  | RMSE | 50.53 | 10.90 | 4.78 | 67.95 |
|  | Train | R^2^ | 0.99 | 0.97 | 0.96 | 0.99 |
|  |  | MAE | 1.83 | 0.81 | 0.35 | 2.41 |
|  |  | RMSE | 5.65 | 1.69 | 0.66 | 4.74 |
| PI-5 | Test | R^2^ | 0.80 | 0.82 | 0.82 | 0.72 |
|  |  | MAE | 45.54 | 2.14 | 1.94 | 94.98 |
|  |  | RMSE | 46.77 | 3.36 | 2.12 | 101.70 |
|  | Train | R^2^ | 0.98 | 0.98 | 0.98 | 0.97 |
|  |  | MAE | 1.71 | 0.82 | 0.32 | 1.96 |
|  |  | RMSE | 3.59 | 1.75 | 0.66 | 4.04 |
| PI-3 | Test | R^2^ | 0.32 | 0.62 | 0.56 | 0.54 |
|  |  | MAE | 67.22 | 7.27 | 3.31 | 54.24 |
|  |  | RMSE | 73.67 | 9.52 | 3.82 | 63.10 |
|  | Train | R^2^ | 0.97 | 0.98 | 0.99 | 0.98 |
|  |  | MAE | 1.50 | 0.80 | 0.31 | 2.04 |
|  |  | RMSE | 3.19 | 1.75 | 0.65 | 4.37 |
| PPO | Test | R^2^ | 0.55 | 0.41 | 0.45 | 0.54 |
|  |  | MAE | 59.82 | 27.67 | 13.61 | 24.44 |
|  |  | RMSE | 62.38 | 29.18 | 13.74 | 30.36 |
|  | Train | R^2^ | 0.98 | 0.97 | 0.98 | 0.99 |
|  |  | MAE | 1.67 | 0.78 | 0.34 | 2.34 |
|  |  | RMSE | 3.44 | 1.68 | 0.68 | 4.77 |
| a-PEO | Test | R^2^ | 0.23 | 0.51 | 0.61 | 0.72 |
|  |  | MAE | 57.00 | 67.62 | 35.05 | 21.46 |
|  |  | RMSE | 57.64 | 68.87 | 36.02 | 24.11 |
|  | Train | R^2^ | 0.97 | 0.98 | 0.98 | 0.99 |
|  |  | MAE | 1.84 | 0.60 | 0.25 | 2.19 |
|  |  | RMSE | 3.74 | 1.26 | 0.49 | 4.56 |
| s-PEO | Test | R^2^ | 0.25 | 0.63 | 0.55 | 0.58 |
|  |  | MAE | 66.90 | 7.36 | 3.36 | 44.91 |
|  |  | RMSE | 73.30 | 9.61 | 3.86 | 53.50 |
|  | Train | R^2^ | 0.97 | 0.99 | 0.98 | 0.98 |
|  |  | MAE | 1.50 | 0.81 | 0.32 | 2.03 |
|  |  | RMSE | 3.15 | 1.75 | 0.65 | 0.99 |

**Table S22** The results of two training strategies (a) and (b) based on the stacking model

| **MMM** | | ***TSP* CO_2_/N_2_** | | ***TSP* CO_2_/O_2_** | | ***TSP* CO_2_/H_2_** | | ***TSP* CO_2_/CH_4_** | | ***P* CO_2_** | |
| --- | --- | --- | --- | --- | --- | --- | --- | --- | --- | --- | --- |
| Polymer | Data(test） | Self | model | Self | model | Self | model | Self | model | Self | model |
| PIM-7 | R^2^ | 0.66 | 0.95 | 0.66 | 0.72 | 0.67 | 0.76 | 0.70 | 0.95 | 0.66 | 0.70 |
|  | MAE | 25.13 | 11.54 | 6.44 | 5.73 | 2.20 | 1.97 | 18.85 | 9.77 | 192.80 | 185.70 |
|  | RMSE | 38.92 | 17.84 | 10.00 | 9.10 | 3.04 | 2.64 | 30.60 | 15.57 | 255.50 | 243.90 |
| PIM-1 | R^2^ | 0.70 | 0.95 | 0.65 | 0.80 | 0.67 | 0.68 | 0.73 | 0.95 | 0.64 | 0.72 |
|  | MAE | 25.97 | 10.93 | 7.97 | 6.48 | 3.30 | 3.30 | 20.92 | 9.70 | 401.20 | 357.10 |
|  | RMSE | 41.38 | 17.53 | 12.67 | 9.51 | 4.60 | 4.50 | 34.76 | 16.48 | 523.10 | 483.30 |
| s-PEO | R^2^ | 0.60 | 0.61 | 0.60 | 0.91 | 0.68 | 0.75 | 0.64 | 0.82 | 0.70 | 0.72 |
|  | MAE | 15.10 | 15.09 | 4.22 | 2.35 | 1.30 | 1.19 | 19.67 | 17.18 | 54.14 | 52.97 |
|  | RMSE | 25.80 | 25.73 | 7.30 | 3.80 | 1.92 | 1.71 | 33.73 | 25.92 | 75.96 | 74.22 |
| a-PEO | R^2^ | 0.68 | 0.84 | 0.43 | 0.47 | 0.58 | 0.61 | 0.64 | 0.70 | 0.72 | 0.74 |
|  | MAE | 2.98 | 2.25 | 7.96 | 8.19 | 4.28 | 4.23 | 11.92 | 11.68 | 16.62 | 17.11 |
|  | RMSE | 5.48 | 4.05 | 15.21 | 15.08 | 7.77 | 7.70 | 23.27 | 21.86 | 26.24 | 25.87 |
| PI-5 | R^2^ | 0.64 | 0.73 | 0.56 | 0.93 | 0.70 | 0.80 | 0.63 | 0.76 | 0.71 | 0.77 |
|  | MAE | 6.68 | 6.07 | 3.46 | 1.58 | 0.92 | 0.80 | 21.85 | 19.51 | 24.21 | 24.69 |
|  | RMSE | 12.28 | 10.91 | 6.59 | 2.80 | 1.42 | 1.17 | 40.48 | 33.01 | 36.70 | 34.05 |
| PI-3 | R^2^ | 0.60 | 0.62 | 0.60 | 0.91 | 0.68 | 0.76 | 0.64 | 0.82 | 0.68 | 0.71 |
|  | MAE | 15.10 | 15.73 | 4.23 | 2.36 | 1.30 | 1.16 | 19.74 | 17.22 | 54.23 | 53.56 |
|  | RMSE | 25.84 | 25.82 | 7.30 | 3.81 | 1.92 | 1.65 | 33.99 | 26.31 | 76.13 | 74.78 |
| PPO | R^2^ | 0.55 | 0.59 | 0.56 | 0.67 | 0.71 | 0.81 | 0.66 | 0.77 | 0.71 | 0.75 |
|  | MAE | 7.73 | 7.68 | 2.04 | 1.81 | 0.63 | 0.55 | 7.07 | 6.18 | 9.25 | 9.87 |
|  | RMSE | 16.14 | 15.14 | 4.26 | 3.66 | 1.06 | 0.87 | 14.76 | 12.35 | 16.17 | 15.21 |
| Matrimind | R^2^ | 0.30 | 0.63 | 0.32 | 0.50 | 0.77 | 0.82 | 0.60 | 0.67 | 0.74 | 0.76 |
|  | MAE | 4.32 | 3.79 | 0.55 | 0.48 | 0.11 | 0.10 | 7.22 | 7.55 | 0.35 | 0.44 |
|  | RMSE | 14.06 | 11.01 | 1.82 | 1.57 | 0.21 | 0.19 | 23.72 | 22.25 | 1.04 | 1.02 |
| CA | R^2^ | 0.32 | 0.55 | 0.25 | 0.54 | 0.61 | 0.68 | 0.63 | 0.86 | 0.75 | 0.80 |
|  | MAE | 2.60 | 2.86 | 0.78 | 0.66 | 0.28 | 0.30 | 3.78 | 4.15 | 0.22 | 0.28 |
|  | RMSE | 8.74 | 7.56 | 2.74 | 2.22 | 0.77 | 0.71 | 12.76 | 13.10 | 0.72 | 0.68 |


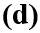

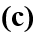
^
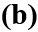
^

**Figure S33.** The transfer learning of CO_2_ separation performance for MMM (PIM-7) by the trained Stacking model. Predicted vs. Simulated (a) TSP(CO_2_/N_2_), (b) TSP(CO_2_/H_2_), (c) TSP(CO_2_/O_2_), (d) TSP(CO_2_/CH_4_).

**References**

**[1].** Dubbeldam, D.; Calero, S.; Ellis, D. E.; Snurr, R. Q., RASPA: molecular simulation software for adsorption and diffusion in flexible nanoporous materials. Molecular Simulation 2016, 42 (2), 81-101.

**[2].** Chung, Y. G.; Haldoupis, E.; Bucior, B. J.; Haranczyk, M.; Lee, S.; Zhang, H.; Vogiatzis, K. D.; Milisavljevic, M.; Ling, S.; Camp, J. S., Advances, updates, and analytics for the computation-ready, experimental metal–organic framework database: CoRE MOF 2019. Journal of Chemical & Engineering Data 2019, 64 (12), 5985-5998.

**[3].** Budd, P. M.; Msayib, K. J.; Tattershall, C. E.; Ghanem, B. S.; Reynolds, K. J.; McKeown, N. B.; Fritsch, D., Gas separation membranes from polymers of intrinsic microporosity. Journal of Membrane Science 2005, 251 (1-2), 263-269.

**[4].** Li, P.; Chung, T.; Paul, D., Gas sorption and permeation in PIM-1. Journal of Membrane Science 2013, 432, 50-57.

**[5].** Rappe, A.K., et al., UFF, a full periodic table force field for molecular mechanics and molecular dynamics simulations. Journal of the American Chemical Society, 1992. 114(25): p. 10024-10035.

**[6].** Budd, P. M.; Msayib, K. J.; Tattershall, C. E.; Ghanem, B. S.; Reynolds, K. J.; McKeown, N. B.; Fritsch, D., Gas separation membranes from polymers of intrinsic microporosity. Journal of Membrane Science 2005, 251 (1), 263-269.

**[7].** Martin, M.G. and J.I. Siepmann, Transferable Potentials for Phase Equilibria. 1. United-Atom Description of n-Alkanes. The Journal of Physical Chemistry B, 1998. 102(14): p. 2569-2577.

**[8].** Shah, M.S., M. Tsapatsis, and J.I. Siepmann, Development of the Transferable Potentials for Phase Equilibria Model for Hydrogen Sulfide. The Journal of Physical Chemistry B, 2015. 119(23): p. 7041-7052.

**[9].** Li, J. R.; Kuppler, R. J.; Zhou, H. C., Selective Gas Adsorption and Separation in Metal-Organic Frameworks. Chem. Soc. Rev. 2009, 38 (5), 1477-504.

**[10].** X.-P. Fu, Y.-L. Wang and Q.-Y. LiuDalton Transactions 2020 Vol. 49 Issue 46 Pages 16598-16607

**[11]**.Chung, Y. G.; Haldoupis, E.; Bucior, B. J.; Haranczyk, M.; Lee, S.; Zhang, H.; Vogiatzis, K. D.; Milisavljevic, M.; Ling, S.; Camp, J. S., Advances, updates, and analytics for the computation-ready, experimental metal–organic framework database: CoRE MOF 2019. *Journal of Chemical & Engineering Data* **2019,** *64* (12), 5985-5998.

**[12]**.Budd, P. M.; Msayib, K. J.; Tattershall, C. E.; Ghanem, B. S.; Reynolds, K. J.; McKeown, N. B.; Fritsch, D., Gas separation membranes from polymers of intrinsic microporosity. *Journal of Membrane Science* **2005,** *251* (1-2), 263-269.

**[13]**.Li, P.; Chung, T.; Paul, D., Gas sorption and permeation in PIM-1. *Journal of Membrane Science* **2013,** *432*, 50-57.

**[14]**.Budd, P. M.; Msayib, K. J.; Tattershall, C. E.; Ghanem, B. S.; Reynolds, K. J.; McKeown, N. B.; Fritsch, D., Gas separation membranes from polymers of intrinsic microporosity. *Journal of Membrane Science* **2005,** *251* (1), 263-269.

**[15]**.Lin, H.; Freeman, B. D., Gas solubility, diffusivity and permeability in poly(ethylene oxide). *Journal of Membrane Science* **2004,** *239* (1), 105-117.

**[16]**. Polotskaya, G. A.; Penkova, A. V.; Toikka, A. M., Fullerene-containing polyphenylene oxide membranes for pervaporation. *Desalination* **2006,** *200* (1), 400-402.

**[17]**.Xie, K.; Fu, Q.; Kim, J.; Lu, H.; He, Y.; Zhao, Q.; Scofield, J.; Webley, P. A.; Qiao, G. G., Increasing both selectivity and permeability of mixed-matrix membranes: Sealing the external surface of porous MOF nanoparticles. *Journal of Membrane Science* **2017,** *535*, 350-356.

**[18]**.Budhathoki, S.; Ajayi, O.; Steckel, J. A.; Wilmer, C. E., High-throughput computational prediction of the cost of carbon capture using mixed matrix membranes. Energy & Environmental Science 2019, 12 (4), 1255-1264.

**[19]**.Bouma, R.; Checchetti, A.; Chidichimo, G.; Drioli, E., Permeation through a heterogeneous membrane: the effect of the dispersed phase. *Journal of Membrane Science* **1997,** *128* (2), 141-149.

**[20]**.Erucar, I.; Keskin, S., Computational screening of metal organic frameworks for mixed matrix membrane applications. *Journal of membrane science* **2012,** *407*, 221-230.

**[21]**.Wijmans, J. G.; Baker, R. W., The solution-diffusion model: a review. *Journal of membrane science* **1995,** *107* (1-2), 1-21.

**[22]**.Shah, M. S.; Tsapatsis, M.; Siepmann, J. I., Identifying optimal zeolitic sorbents for sweetening of highly sour natural gas. *Angewandte Chemie International Edition* **2016,** *55* (20), 5938-5942.

**[23]**.P. M. Budd, K. J. Msayib, C. E. Tattershall, B. S. Ghanem, K. J. Reynolds, N. B. McKeown, et al.Journal of Membrane Science 2005 Vol. 251 Issue 1-2 Pages 263-269

**[24]**.W. Fang, L. Zhang and J. Jiang,Molecular Simulation 2010 Vol. 36 Issue 12 Pages 992-1003

**[25]**.H. Lin and B. D. Freeman.Journal of Membrane Science 2004 Vol. 239 Issue 1 Pages 105-117.

**[26]**.Polotskaya, G. A., Penkova, A. V., Toikka, A. M., Pientka, Z., Brozova, L., & Bleha, M.(2007). Transport of Small Molecules through Polyphenylene Oxide Membranes Modified by Fullerene. Separation Science and Technology, 42(2), 333–347.

**[27]**.B.N.Gacal,V.Filiz,S.Shishatskiy,S.Neumann,J.Wind,V.Abetz,J.Membr.Sci.467(2014)126.

**[28].** E. Esposito, I. Mazzei, M. Monteleone, A. Fuoco, M. Carta, N.B. McKeown, R. Malpass-Evans, J.C. Jansen Polymers, 11 (2019), p. 46

**[29].**M. Al-Masri, H. R. Kricheldorf and D. Fritsch.Macromolecules 1999 Vol. 32 Issue 23 Pages 7853-7858

**[30].**A. Perea-Cachero, J. Sánchez-Laínez, Á. Berenguer-Murcia, D. Cazorla-Amorós, C. Téllez and J. Coronas, Journal of Membrane Science 2017 Vol. 544 Pages 88-97

**[31].**M. Z. Ahmad, M. Navarro, M. Lhotka, B. Zornoza, C. Téllez, W. M. de Vos, et al.

Journal of membrane science 2018 Vol. 558 Pages 64-77

**[32]**.J. D. Rodriguez, A. Perez, J. A. Lozano, IEEE transactions on pattern analysis and machine intelligence 2009, 32, 569-575.

**[33].**Pradhan, B. and S. Lee, Regional landslide susceptibility analysis using back-propagation neural network model at Cameron Highland, Malaysia. Landslides, 2010. 7(1): p. 13-30.

**[34].** Breiman, L., Random Forests. Machine Learning, 2001. 45(1): p. 5-32.

**[35].** https://xgboost.readthedocs.io/en/stable/.

**[36].** Friedman, J. H. Greedy function approximation: A gradient boosting machine. Ann. Stat. 2001, 29 (5), 1189-1232.

**[37].** COVER, T. M.; HART, P. E. Nearest Neighbor Pattern Classification IEEE Trans. Inf Theory 1967, 13 (1), 21.

**[38].**Pavlyshenko, B. In Using stacking approaches for machine learning models, 2018 IEEE Second International Conference on Data Stream Mining & Processing (DSMP), IEEE: 2018; pp 255-258.

**[39]** C. Altintas, G. Avci, H. Daglar, E. Gulcay, I. Erucar, S. Keskin, Computer simulations of 4240 MOF membranes for H2/CH4 separations: insights into structure-performance relations, J. Mater. Chem. A 6(14) (2018) 5836-5847.

**[40]** G. Avci, S. Velioglu, S. Keskin, High-throughput screening of MOF adsorbents and membranes for H2 purification and CO_2_ capture, ACS Appl. Mater. Interfaces 10(39) (2018) 33693-33706.

**[41]** H. Daglar, S. Keskin, Computational screening of metal-organic frameworks for membrane-based CO_2_/N_2_/H_2_O separations: best materials for flue gas separation, J. Phys. Chem. C 122(30) (2018) 17347-17357.

**[42]** C. Altintas, S. Keskin, Molecular simulations of MOF membranes and performance predictions of MOF/polymer mixed matrix membranes for CO_2_/CH_4_ separations, ACS Sustain. Chem. Eng. 7(2) (2019) 2739-2750.

**[43]** H. Daglar, S. Keskin, Recent advances, opportunities, and challenges in high-throughput computational screening of MOFs for gas separations, Coord. Chem. Rev. 422 (2020) 213470.

**[44]** H. Daglar, I. Erucar, S. Keskin, Exploring the performance limits of MOF/polymer MMMs for O_2_/N_2_ separation using computational screening, J. Membr. Sci. 618 (2021) 118555.

**[45]** J. Glover, E. Besley, A high-throughput screening of metal-organic framework based membranes for biogas upgrading, Faraday Discuss. 231(0) (2021) 235-257.

**[46]** M.S. Shah, M. Tsapatsis, J.I. Siepmann, Identifying optimal zeolitic sorbents for sweetening of highly sour natural gas, Angew. Chem. Int. Ed. 128(20) (2016) 6042-6046.

**[47]** N. Hara, M. Yoshimune, H. Negishi, K. Haraya, S. Hara, T. Yamaguchi, Metal–organic framework membranes with layered structure prepared within the porous support, RSC Adv. 3(34) (2013) 14233-14236.

**[48]** Y. Mao, L. shi, H. Huang, W. Cao, J. Li, L. Sun, X. Jin, X. Peng, Room temperature synthesis of free-standing HKUST-1 membranes from copper hydroxide nanostrands for gas separation, Chem. Commun. 49(50) (2013) 5666-8.

**[49]** W. Wu, Z. Li, Y. Chen, W. Li, Polydopamine-modified metal-organic framework membrane with enhanced selectivity for carbon capture, Environ Sci Technol 53(7) (2019) 3764-3772.

**[50]** Y. Yoo, Z. Lai, H.-K. Jeong, Fabrication of MOF-5 membranes using microwave-induced rapid seeding and solvothermal secondary growth, Microporous Mesoporous Mater. 123(1-3) (2009) 100-106.

**[51]** A. Huang, H. Bux, F. Steinbach, J. Caro, Molecular-sieve membrane with hydrogen permselectivity: ZIF-22 in LTA topology prepared with 3-aminopropyltriethoxysilane as covalent linker, Angew. Chem. Int. Ed. Engl. 49(29) (2010) 4958-4961.

**[52]** E. Gulcay-Ozcan, I. Erucar, Biocompatible MOFs for Storage and Separation of O2: A Molecular Simulation Study, Ind. Eng. Chem. Res. 58(8) (2019) 3225-3237.

**[53]** I. Erucar, S. Keskin, Computational Modeling of bio-MOFs for CO_2_/CH_4_ separations, Chem. Eng. Sci. 130 (2015) 120-128.

**[54]** T. Watanabe, D.S. Sholl, Accelerating applications of metal-organic frameworks for gas adsorption and separation by computational screening of materials, Langmuir 28(40) (2012) 14114-14128.

**[55].** Lundberg, S. M.; Erion, G.; Chen, H.; DeGrave, A.; Prutkin, J. M.; Nair, B.; Katz, R.; Himmelfarb, J.; Bansal, N.; Lee, S. I., From Local Explanations to Global Understanding with Explainable AI for Trees. Nat. Mach. Intell. 2020, 2 (1), 56-67.

**[56]**. Perez, E. V., Balkus Jr, K. J., Ferraris, J. P., Musselman, I. H. Mixed-Matrix Membranes Containing MOF-5 for Gas Separations. J. Membr. Sci. 2009, 328, 165-173.

**[57]**. Ge, L., Zhou, W., Rudolph, V., Zhu, Z. H. Mixed Matrix Membranes Incorporated with Size-Reduced Cu-BTC for Improved Gas Separation. J. Mater. Chem. A 2013, 1, 6350- 6358

**[58]**. Hsieh, J. O., Balkus, K. J., Ferraris, J. P., Musselman, I. H. MIL-53 Frameworks in Mixed-Matrix Membranes. Microporous Mesoporous Mater. 2014, 196, 165-174.

**[59]**. Dorosti, F., Omidkhah, M., Abedini, R. Fabrication and Characterization of Matrimid /MIL-53 Mixed Matrix Membrane for CO_2_/CH_4_ Separation. Chem. Eng. Res. Des. 2014, 92, 2439-2448.

**[60]**. Chen, X. Y., Vinh-Thang, H., Rodrigue, D., Kaliaguine, S. Amine-Functionalized MIL53 Metal-Organic Framework in Polyimide Mixed Matrix Membranes for CO_2_/CH_4_ Separation. Ind. Eng. Chem. Res. 2012, 51, 6895-6906.

**[61]**. Ordonez, M. J. C., Balkus Jr, K. J., Ferraris, J. P., Musselman, I. H. Molecular Sieving Realized with ZIF-8/Matrimid Mixed-Matrix Membranes. J. Membr. Sci. 2010, 361, 28- 37.

**[62]**. Japip, S., Wang, H., Xiao, Y., Shung Chung, T. Highly Permeable Zeolitic Imidazolate Framework (ZIF)-71 Nano-Particles Enhanced Polyimide Membranes for Gas Separation. J. Membr. Sci. 2014, 467, 162-174.

**[63]**. Bushell, A. F., Attfield, M. P., Mason, C. R., Budd, P. M., Yampolskii, Y., Starannikova, L., Rebrov, A., Bazzarelli, F., Bernardo, P., Carolus Jansen, J., Lanč, M., Friess, K., Shantarovich, V., Gustov, V., Isaeva, V. Gas Permeation Parameters of Mixed Matrix Membranes based on the Polymer of Intrinsic Microporosity PIM-1 and the Zeolitic Imidazolate Framework ZIF-8. J. Membr. Sci. 2013, 427, 48-62.

**[64]**. Li, H., Tuo, L., Yang, K., Jeong, H.-K., Dai, Y., He, G., Zhao, W. Simultaneous Enhancement of Mechanical Properties and CO_2_ Selectivity of ZIF-8 Mixed Matrix Membranes: Interfacial Toughening Effect of Ionic Liquid. J. Membr. Sci. 2016, 511, 130-142.

**[65].** Bae, T.-H., Lee, J. S., Qiu, W., Koros, W. J., Jones, C. W., Nair, S. A High-Performance Gas-Separation Membrane Containing Submicrometer-Sized Metal–Organic Framework Crystals. Angew. Chem. Int. Ed. 2010, 49, 9863-9866. S-11

**[66]**. Nik, O. G., Chen, X. Y., Kaliaguine, S. Functionalized Metal Organic FrameworkPolyimide Mixed Matrix Membranes for CO_2_/CH_4_ Separation. J. Membr. Sci. 2012, 413- 414, 48-61.

**[67]**. Rodenas, T., Luz, I., Prieto, G., Seoane, B., Miro, H., Corma, A., Kapteijn, F., Llabrés i Xamena, F. X., Gascon, J. Metal–Organic Framework Nanosheets in Polymer Composite Materials for Gas Separation. Nat. Mater. 2015, 14, 48-55.

**[68]**. S. J. Smith, B. P. Ladewig, A. J. Hill, C. H. Lau, M. R. Hill, Sci. Rep. 2015, 5, 7823.

**[69]**. T. Li, Y. Pan, K.-V. Peinemann, Z. Lai, J. Membr. Sci. 2013, 425, 235–242.

**[70]**. W. S. Chi, S. Hwang, S.-J. Lee, S. Park, Y.-S. Bae, D. Y. Ryu, J. H. Kim, J. Kim, J. Membr. Sci. 2015, 495, 479–488.

**[71]**. V. Nafisi, M.-B. Hägg, J. Membr. Sci. 2014, 459, 244–255.

**[72]**. L. Dong, M. Chen, J. Li, D. Shi, W. Dong, X. Li, Y. Bai, J. Membr. Sci. 2016, 520, 801– 811.

**[73]**. Su, N.C.; Sun, D.T.; Beavers, C.M.; Britt, D.K.; Queen, W.L.; Urban, J.J. Enhanced permeation arising from dual transport pathways in hybrid polymer–MOF membranes. Energy Environ. Sci. 2016, 9, 922–931.

**[74].** Khdhayyer, M.R.; Esposito, E.; Fuoco, A.; Monteleone, M.; Giorno, L.; Jansen, J.C.; Attfield, M.P.; Budd, P.M. Mixed matrix membranes based on UiO-66 MOFs in the polymer of intrinsic microporosity PIM-1. Sep. Purif. Technol. 2017, 173, 304–313.

**[75]**. Prasetya, N.; Donose, B.C.; Ladewig, B.P. A new and highly robust light-responsive Azo-UiO-66 for highly selective and low energy post-combustion CO_2_ capture and its application in a mixed matrix membrane for CO_2_/N_2_ separation. *J. Mater. Chem. A* **2018**, *6*, 16390–16402.

**[76]**. Shen, J.; Liu, G.; Huang, K.; Li, Q.; Guan, K.; Li, Y.; Jin, W. UiO-66-polyether block amide mixed matrix membranes for CO_2_ separation. J. Membr. Sci. 2016, 513, 155–165.

**[77].** W. Chen, Z. Zhang, L. Hou, C. Yang, H. Shen, K. Yang, Z. Wang, Metal-organic framework MOF-801/PIM-1 mixed-matrix membranes for enhanced CO_2_/N_2_ separation performance Sep. Purif. Technol. (2020), Article 117198, 10.1016/j.seppur.2020.117198.

**[78]**. R. Lin, L. Ge, H. Diao, V. Rudolph, Z. Zhu, Ionic liquids as the MOFs/Polymer interfacial binder for efficient membrane separation, ACS Appl. Mater. Interfaces (2016), pp. 32041-32049, 10.1021/acsami.6b11074

**[79]**. J. Ma, Y. Ying, X. Guo, H. Huang, D. Liu, C. Zhong, Fabrication of mixed-matrix membrane containing metal–organic framework composite with task-specific ionic liquid for efficient CO_2_ separation, J. Mater. Chem. A. (2016), pp. 7281-7288, 10.1039/C6TA02611G
